# Antimicrobial Peptide–Peptoid Hybrids with and without Membrane Disruption

**DOI:** 10.1021/acsinfecdis.3c00421

**Published:** 2023-11-21

**Authors:** Etienne Bonvin, Hippolyte Personne, Thierry Paschoud, Jérémie Reusser, Bee-Ha Gan, Alexandre Luscher, Thilo Köhler, Christian van Delden, Jean-Louis Reymond

**Affiliations:** 1 Department of Chemistry, Biochemistry and Pharmaceutical Sciences, University of Bern, Freiestrasse 3, CH-3012 Bern, Switzerland; 2 Department of Microbiology and Molecular Medicine, University of Geneva, CH-1211 Geneva, Switzerland; 3 Service of Infectious Diseases, University Hospital of Geneva, CH-1211 Geneva, Switzerland

**Keywords:** Antimicrobial peptides, peptoids, membrane disruption, secondary structure

## Abstract

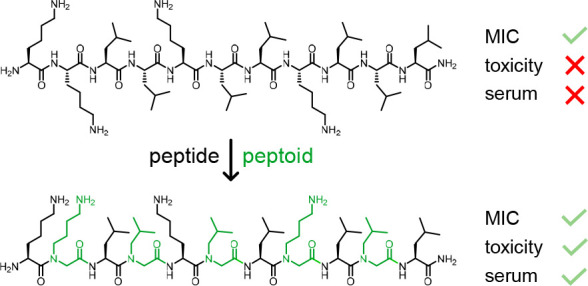

Among synthetic analogues of antimicrobial peptides (AMPs) under investigation to address antimicrobial resistance, peptoids (*N*-alkylated oligoglycines) have been reported to act both by membrane disruption and on intracellular targets. Here we gradually introduced peptoid units into the membrane-disruptive undecapeptide KKLLKLLKLLL to test a possible transition toward intracellular targeting. We found that selected hybrids containing up to five peptoid units retained the parent AMP’s α-helical folding, membrane disruption, and antimicrobial effects against Gram-negative bacteria including multidrug-resistant (MDR) strains of *Pseudomonas aeruginosa* and *Klebsiella pneumoniae* while showing reduced hemolysis and cell toxicities. Furthermore, some hybrids containing as few as three peptoid units as well as the full peptoid lost folding, membrane disruption, hemolysis, and cytotoxicity but displayed strong antibacterial activity under dilute medium conditions typical for proline-rich antimicrobial peptides (PrAMPs), pointing to intracellular targeting. These findings parallel previous reports that partially helical amphiphilic peptoids are privileged oligomers for antibiotic development.

The rise of antimicrobial resistance worldwide calls for the development of new antibiotics.^1,2^ Antimicrobial peptides (AMPs), which occur in all domains of life as part of the innate immune response,^3^ offer favorable starting points to develop new antimicrobial agents. Most AMPs are cationic amphiphiles acting by disrupting the bacterial membrane.^4−8^ This type of activity is often preserved or increased in analogues with modified sequence or peptide chain topology designed to improve their pharmacokinetic and toxicity profile.^9−13^ Membrane-disruptive antibacterial activities have also been reported with various polymers,^14^ dendrimers,^15−21^ as well as with nonpeptidic oligomers,^22−25^ in particular with peptoids, in which amino acid side chains are attached to the amide nitrogen rather than to the α-carbon atom.^26−28^

Although lacking hydrogen-bonding amide NH groups and therefore unable to form canonical peptide secondary structures, peptoids designed to fold into amphiphilic polyproline-like type I helices by introducing chiral side chains have been shown to display membrane-disruptive antibacterial activities and tunable helicity and toxicity.^29−40^ Interestingly however, polycationic and amphiphilic antimicrobial peptoids were also reported which appear not to act as membrane disruptors but rather on intracellular targets.^41−45^ Such intracellular targeting is reminiscent of proline-rich antimicrobial peptides (PrAMPs) such as the nonadecapeptide oncocin (VDKPPYLPRPRPPRRIYNR), which have a reduced number of amide NH groups and act on intracellular targets including various sites on the ribosome as well as the heat shock protein DnaK (Hsp70).^46−52^ This analogy suggests that gradually substituting amino acids with their peptoid equivalents in an AMP sequence, and thereby reducing the number of backbone amide NH groups, might decrease membrane-disruptive effects and at some point enable intracellular targeting. Although introducing peptoid building blocks has been previously investigated as a method to tune AMP activity,^53−58^ a systematic study of the effect of peptoid building blocks on AMP antibacterial activity and mechanism has not been previously reported.

Here we addressed this question for the case of the undecapeptide **ln65** with sequence KKLLKLLKLLL, an α-helical membrane-disruptive lysine–leucine AMP showing strong activities against Gram-negative bacteria discovered during a virtual screening campaign aimed at bicyclic AMPs.^13^ Because this AMP appeared tolerant to the introduction of d-residues at various positions,^59^ e.g., **ln69** (kkLLkLLkLLL) bearing four d-lysines and showing full α-helicity and activity but strongly reduced hemolysis and full stability against proteolysis in serum, we wondered whether **ln65** might also tolerate the presence of peptoid building blocks, and whether these might favorably affect toxicity and stability.

As detailed below, we found that α-helical folding and the associated membrane-disruptive antimicrobial effects could be preserved upon introducing up to five peptoid units in the sequence, resulting in peptide–peptoid hybrids such as **EB5** with an activity/toxicity profile comparable to the mixed chirality AMP **ln69**. Furthermore, we discovered that analogues containing as few as three peptoid units such as **EB2** and **EB3**, **EB9** with alternating peptoid and peptide units, or the full peptoid **EB11**, lacked membrane-disruptive effects and displayed strong antibacterial effects when tested in dilute growth medium (12.5% MH) typical for testing PrAMPs^46,47,49,50^ and which better reproduces the physiological conditions,^60^ while showing no hemolysis and very low toxicity against eukaryotic cells (Table 1).

**Table 1:** Antimicrobial Activities of Peptide–Peptoid Hybrids

		MIC^b^ (μg/mL)											CD
		*E. coli* W3110		*P. aeruginosa* PAO1		*A. baumannii* ATCC19606		*K. pneumoniae* NCTC418		*S. aureus* COL (MRSA)		MHC^c^ (μg/mL)	%α^d^
**No.**	Sequence^a^	Full	12.5%	Full	12.5%	Full	12.5%	Full	12.5%	Full	12.5%		
**PMB**^e^		0.25	0.5	0.5	0.5	0.2	0.5	0.25	0.5	>32	8	>2000	n.d.
**Onc**^f^		4	1	>32	32	32	4	4	1	>32	32	>1000	20/23
**ln65**	KKLLKLLKLLL	4	2	2–4	4	2–4	4	4	2–4	4	2	125	73/64
**ln69**	kkLLkLLkLLL	4	2	8	4	2–4	2	8	4	16	2	1000	61/34
**EB1**	*k*KLLKLLKLL*l*	2	2	4	2	2	2	8	2	8	2	1000	30/26
**EB2**	*k*KLLKLL*k*LL*l*	32	4	32	16	>32	16	>32	>32	>32	32	>2000	11/15
**EB3**	KKLLKLLK*lll*	>32	4	>32	16	>32	32	>32	>32	>32	32	>2000	13/15
**EB4**	KKLLK*llklll*	>32	8	>32	8–16	>32	32	>32	>32	>32	32	>2000	9/11
**EB5**	*kkllk*LLKLLL	2	2	4	4	2	2	>32	16	8	4	1000	24/25
**EB6**	*kk*LL*k*LLKLLL	2	2	4	2	2	2	8	4	8	2	250	41/35
**EB7**	*kk*LL*k*LL*k*LLL	16	4	32	16	>32	16	>32	32	>32	16	>2000	12/13
**EB8**	KK*ll*K*ll*K*lll*	>32	8	>32	>32	>32	32	>32	>32	>32	32	>2000	11/15
**EB9**	K*k*L*l*K*l*L*k*L*l*L	16	2	32	4	>32	16	>32	>32	>32	32	>2000	11/13
**EB10**	*k*K*l*L*k*L*l*K*l*L*l*	>32	2–4	>32	4	>32	8–16	>32	32	>32	8	>2000	11/11
**EB11**	*kkllkllklll*	>32	8	>32	8	>32	16	>32	32	>32	>32	>2000	7/8

a One letter code for amino acids. The d-amino acids are denoted with the small letters, and *N*-substituted residues are indicated in italics; k = d-lysine, *k* = *N*Lys (lysine-like residue), *l* = *N*Leu (leucine-like residue).

b Minimum inhibitory concentration (MIC), in μg/mL, was determined on bacteria in Mueller–Hinton broth (MH) at pH 7.4 (full MH, pH 7.4) or in diluted MH at pH 8.5 (12.5% MH, pH 8.5) after incubation for 16–20 h at 37 °C. Values represent two different duplicate MIC determinations.

c Minimum Hemolytic Concentration (MHC) measured on human red blood cells (hRBC) in 10 mM phosphate buffer, 150 mM NaCl, pH 7.4, 25 °C, 4 h.

d CD spectra were recorded at 0.1 mg/mL in aqueous 10 mM phosphate buffer at pH 7.4 with the addition of 5 mM DPC or with 20% TFE. The primary CD spectra were analyzed using DichroWeb, and the percentages of α-helical signals were extracted. Every building block (peptoid or amino acid) was taken into account for the calculations. The Contin-LL method and reference set 4 were used.^63^ The data represent α-helicity percentage with 5 mM DPC/20% TFE.

e Polymyxin B.

f Oncocin, sequence VDKPPYLPRPRPPRRIYNR. “n.d.” = not determined.

## Results

### Design and Synthesis

Peptoid residues are *N*-alkylated glycines lacking the amide NH group and therefore acting as α-helix breakers. Accordingly, we first selected a few sequences displaying a continuous stretch of 5 to 9 amino acids susceptible to preserving partial α-helicity, expected to be necessary for antimicrobial activity. These included sequences with one peptoid each at the *C*- and *N*-terminus (**EB1**), optionally with an additional lysine peptoid unit at position 8 (**EB2**), or contiguous peptoid stretches at the *C*- (**EB3**: 3 residues, **EB4**: 6 residues) or *N*-terminus (**EB5**: 4 residues). Alternatively, we exchanged three (**EB6**) or four (**EB7**) of the lysine residues for peptoids in analogy to AMP **ln69** bearing four d-lysines, or on the contrary exchanged all leucines for peptoids (**EB8**). Finally, we prepared **EB9** and **EB10** with alternating peptide and peptoid building blocks and full peptoid **EB11** (Table 1).

**Scheme 1:**
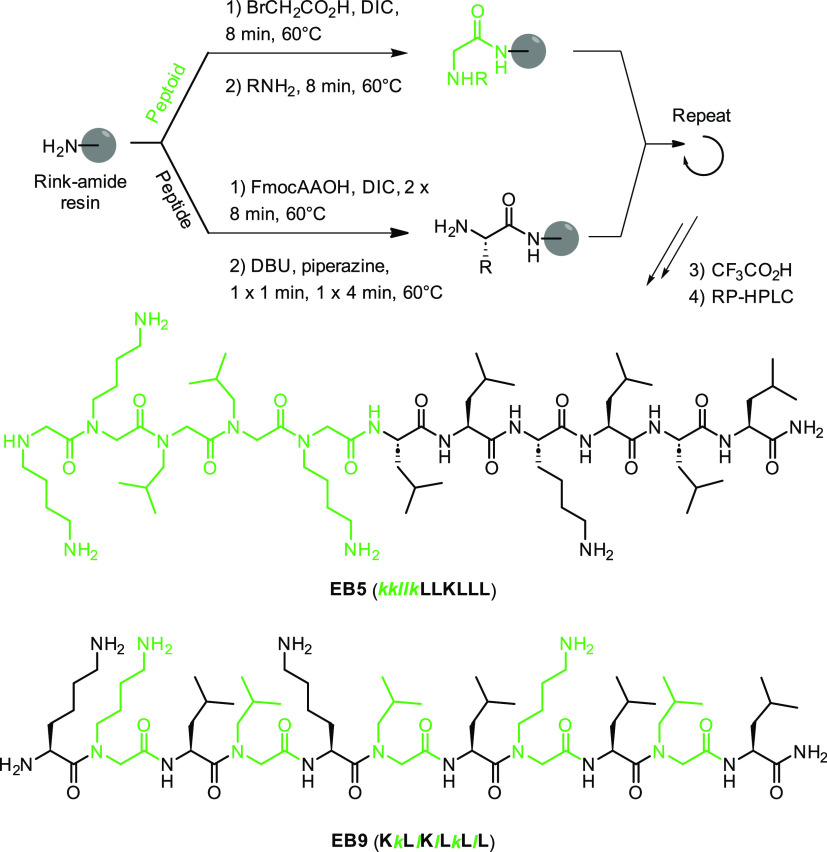
SPPS of Peptide–Peptoid Hybrids and Structural Formula of **EB5** and **EB9**

Peptoid units can be introduced during standard Fmoc solid-phase peptide synthesis (Fmoc-SPPS) using the so-called submonomer strategy,^26^ which consists in bromoacetylation of the *N*-terminus of the growing chain followed by substitution of the bromide using an excess of a primary amine, here isobutylamine for a leucine peptoid unit (*N*Leu) or *tert*-butyl (4-aminobutyl)carbamate for a lysine peptoid unit (*N*Lys). We prepared the eleven selected peptide–peptoid hybrids together with undecapeptides **ln65** and **ln69**, as well as the PrAMP oncocin, to be used as positive controls,^46^ using high-temperature (60 °C) semiautomated Fmoc-SPPS on Rink amide resin in DMF with di-isopropyl carbodiimide (DIC) and OxymaPure^61^ as coupling reagents as described previously.^21,59^ Addition of amino acids was repeated twice, while acylation with bromoacetic acid and displacement with the primary amine were performed only once. All products were obtained in pure form by acidic cleavage and deprotection followed by preparative reversed-phase HPLC (Scheme 1).

All compounds were evaluated by measuring minimal inhibitory concentrations (MIC) in a standard 2-fold serial dilution protocol against *Escherichia coli*, *Pseudomonas aeruginosa*, *Acinetobacter baumannii*, *Klebsiella pneumoniae*, and methicillin-resistant *Staphylococcus aureus*, together with polymyxin B (**PMB**), oncocin (**Onc**), and the parent AMPs **ln65** and **ln69** as references. We tested activities in full Muller–Hinton (MH) medium, as well as in diluted medium (12.5% MH), which are conditions under which **Onc** shows its activity, an effect attributed to the induction of nutrient transporters favoring cellular uptake.^62^ Indeed, while the references **PMB**, **ln65**, and **ln69** were not affected by medium dilution, **Onc** showed the expected activity increase in dilute medium (4-fold against *E. coli*, *A. baumannii*, and *K. pneumoniae* and switch from inactive to slightly active against *P. aeruginosa* and MRSA, Table 1 and S2). We also measured minimal hemolysis concentrations (MHC) on human red blood cells by serial 2-fold dilution as an indication of toxicity against eukaryotic cells (Table 1).

### Antimicrobial Activity, Toxicity, and Serum Stability

**Figure 1:**
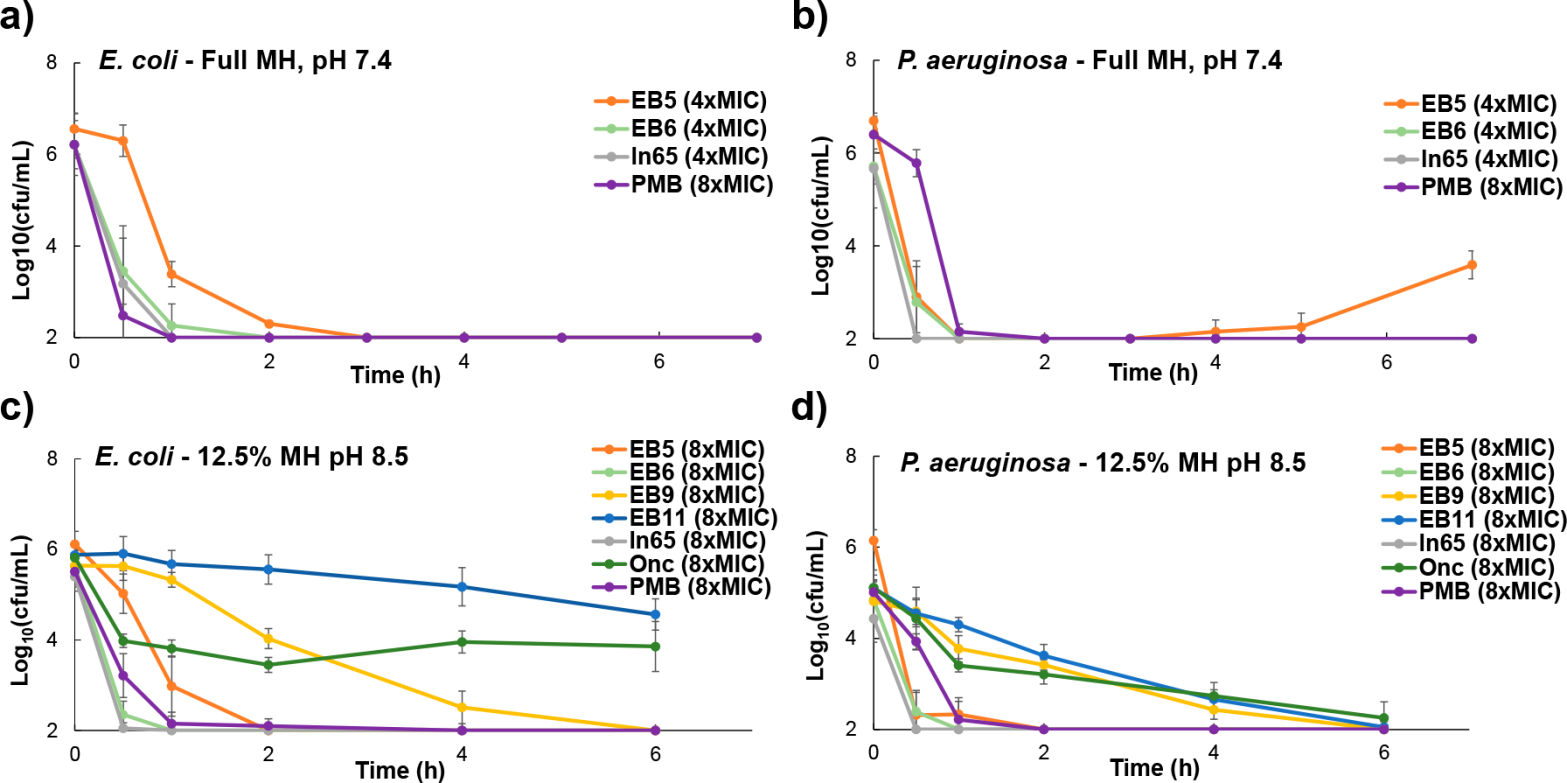
Killing profile of selected compounds on bacteria a) against *E. coli* W3110 measured in full MH at pH 7.4, b) against *P. aeruginosa* PAO1 in full MH at pH 7.4, c) against *E. coli* W3110 in diluted MH at pH 8.5, and d) against *P. aeruginosa* PAO1 in dilute MH at pH 8.5. The assay was repeated twice in triplicate, and the data represent the mean ± SD, *n* = 6.

When tested in full MH, three of the peptide–peptoid hybrids (**EB1**, **EB5**, and **EB6**) were consistently active against the five bacterial species tested to levels comparable to the parent AMPs **ln65** and **ln69** (MIC = 2–8 μg/mL) and showed similar hemolysis (MHC = 250–1000 μg/mL). In time–kill assays, **EB6** completely killed both *E. coli* and *P. aeruginosa* in full MH within few hours, similarly to **ln65** and **ln69**, while **EB5** showed a rebound with PAO1 after 3 h, suggesting incomplete killing in that case (Figure 1a/b).

The three peptide*–*peptoid hybrids above were similarly active in a dilute medium (12.5% MH, pH 7.4). Strikingly, however, all peptide–peptoid hybrids that were inactive in full MH (**EB2**, **EB3**, **EB4**, **EB7**, **EB8**, **EB9**, **EB10**, and **EB11**) showed very significant activities against at least two different bacteria in 12.5% MH at pH 7.4, while none of them showed any measurable hemolysis (Table 1 and S2). The effect was further increased when raising the pH to 8.5, which we have found previously to increase the activity of **PMB** and peptide dendrimers.^21,64,65^ Under these dilute, slightly alkaline conditions, all peptide–peptoid hybrids showed strong antibacterial effects. The two most striking examples were **EB9** with alternating peptide and peptoid units and full peptoid **EB11**. Time–kill assays with *E. coli* W3110 and *P. aeruginosa* PAO1 showed that both compounds killed *P. aeruginosa*, however at a rather slow rate comparable to that of **Onc**. Bacteria could still be detected after 6 h in the case of *E. coli* (Figure 1c/d).

**Figure 2:**
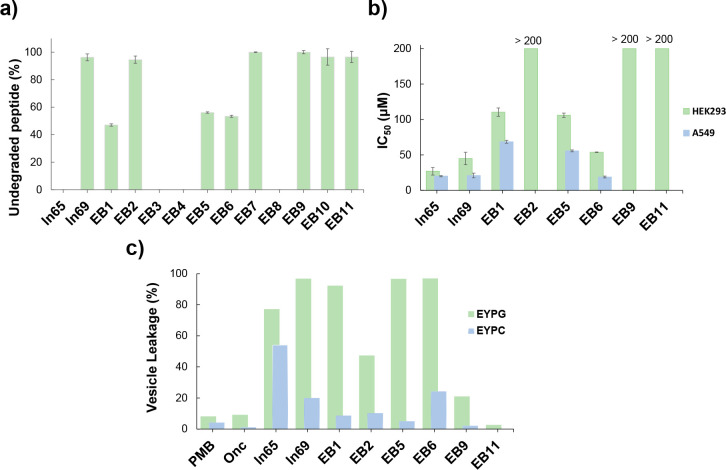
(a) Stability of the peptides (200 μM) against proteolysis in human serum (12.5% in TRIS buffer, 0.1 M, pH 7.4), after 24 h at 37 °C. Undegraded peptide values determined by RP-HPLC analysis using 4-hydroxybenzoic acid as internal standard. **ln65**, **EB3**, **EB4**, and **EB8** are completely degraded. (b) Toxicity evaluation for selected compounds on HEK293 cells and, for the most active compounds, on cancer cells A549. All data are represented as the IC_50_ value measured by Alamar blue assay after 24 h treatments with concentrations from 0 to 200 μM. The data are represented as the mean value ± SD, *n* = 3. The toxicity of **EB2**, **EB9**, and **EB11** on A549 cells was not determined. (c) Vesicle leakage assay for a selection of compounds. Lipid vesicles made of EYPG or EYPC were suspended in a buffer (10 mM TRIS, 107 mM NaCl, pH 7.4). After 45 s, the indicated compound was added to reach the desired concentration. After 240 s, 30 μL of 1.2% TritonX-100 was added for full fluorescein release. The percentage leakage observed with the 10 μg/mL compound at 220 s is given. See Supporting Information for the full curves, all of the data, and procedures.

As could be anticipated from their composition, most peptide–peptoid hybrids were entirely stable because peptoids cannot be cleaved by proteases (Figure 2a). Notable exceptions were **EB3**, **EB4**, and **EB8**, suggesting that their lack of antibacterial activity in full medium might be related to degradation under these conditions. Further evaluation of the most active peptide–peptoid hybrids (**EB1**, **EB5, EB6**, **EB9**, and **EB11**) showed that antibacterial effects were preserved against several multidrug-resistant strains of *P. aeruginosa* and clinically relevant pathogens, whereby activities were generally stronger in 12.5% MH pH 8.5 compared to full MH pH 7.4 (Table 2 and S3). All of these compounds showed acceptable toxicities against HEK293 cells, in particular **EB9** and **EB11** for which no effect was detected up to 200 μM (Figure 2b). Taken together, these data showed that several peptide–peptoid hybrids could reach activities comparable to the parent AMPs **ln65** and **ln69** either in full MH or in dilute MH while maintaining low hemolysis and toxicity as well as excellent stability in human serum.

**Table 2:** Activities against an Extended Panel of MDR Bacteria

	**MIC**^a^											
Cpd	*P. aeruginosa* PA14	PA14 4.13 (phoQ)	PA14 4.18 (pmrB)	PA14 2P4 (pmrB)	ZEM-1A	ZEM9A	*E. cloacae*	*K. pneumoniae* OXA-48	*S. maltophilia*	*B. cenocepacia*	*S. aureus* Newman	*S. epidermis*
**PMB**	<0.13	0.25	1	1	<0.13	2	0.5–1	1–2	0.5	>16	>16	>16
**ln65**	2–4	4	32	32	2	4	2	2	2	>32	2	2
**ln69**	2	4	16	32	2	4–8	4	4	2	>32	8	2–4
**EB1**	4	8	32	32	2–4	16	8	8–16	2–4	>32	32	4–8
**EB5**	4–8	8	32	>32	2	8	8–16	32–64	8	>32	>32	4–8
**EB6**	2–4	4	16	32	2	4	4	8	2–4	>32	8	2–4
**Onc**^b^	2	2	2	2	2	16	2	1	>32	32	1	4
**EB9**^b^	2	2	4–8	4	2–4	2	16	32	8	>32	4	2
**EB11**^b^	2–4	4–8	8–16	8–16	2–4	4	16	32	4	>32	4	1

a Minimum inhibitory concentrations (in μg/mL) were determined in Mueller–Hinton broth (MH) at pH 7.4 after incubation for 16–20 h at 37 °C. Values represent two different duplicate MIC determinations.

b MIC values were determined in diluted MH (12.5% MH) at pH 8.5 for **Onc**, **EB9**, and **EB11**. ZEM-1A and ZEM9A are two clinical MDR *P. aeruginosa* isolates.

### α-Helical Folding and Membrane Interactions

The peptide–peptoid hybrids **EB1**–**EB11** have the same molecular mass as the parent AMPs **ln65** and **ln69** and the same sequence and therefore relative arrangement of charged and hydrophobic groups. Analytical HPLC data also showed that they have very similar hydrophobicity (Supporting Information, Table S1). Their very different biological activities must therefore reflect other differences, presumably in their conformation.

**Figure 3:**
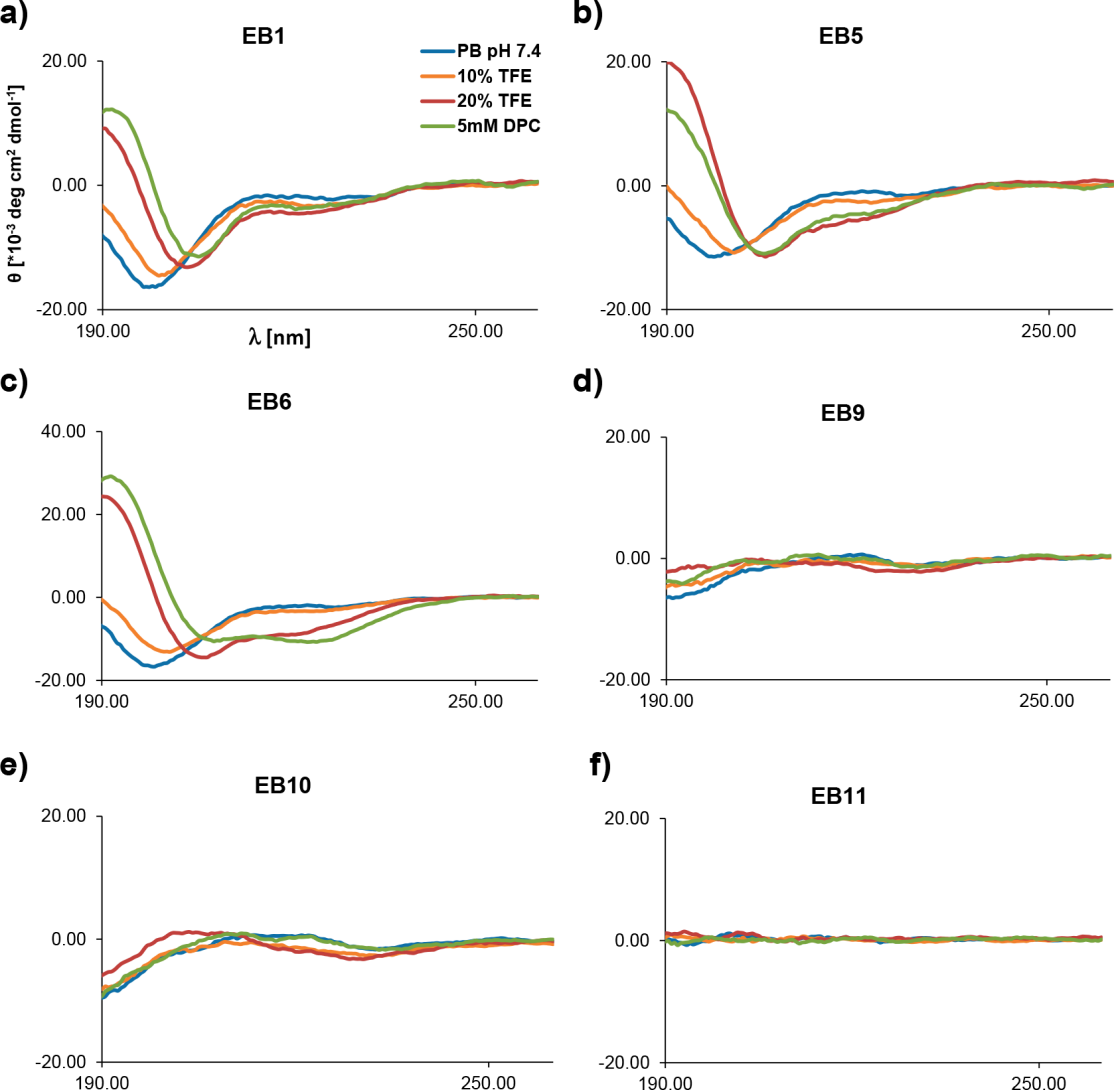
Circular Dichroism (CD) spectra measured with 0.1 mg/mL of selected peptides, in 7 mM phosphate buffer at pH 7.4 (blue line), in the presence of different amounts of TFE (10 or 20%, orange and red line, respectively), and 5 mM DPC (green line).

**Figure 4:**
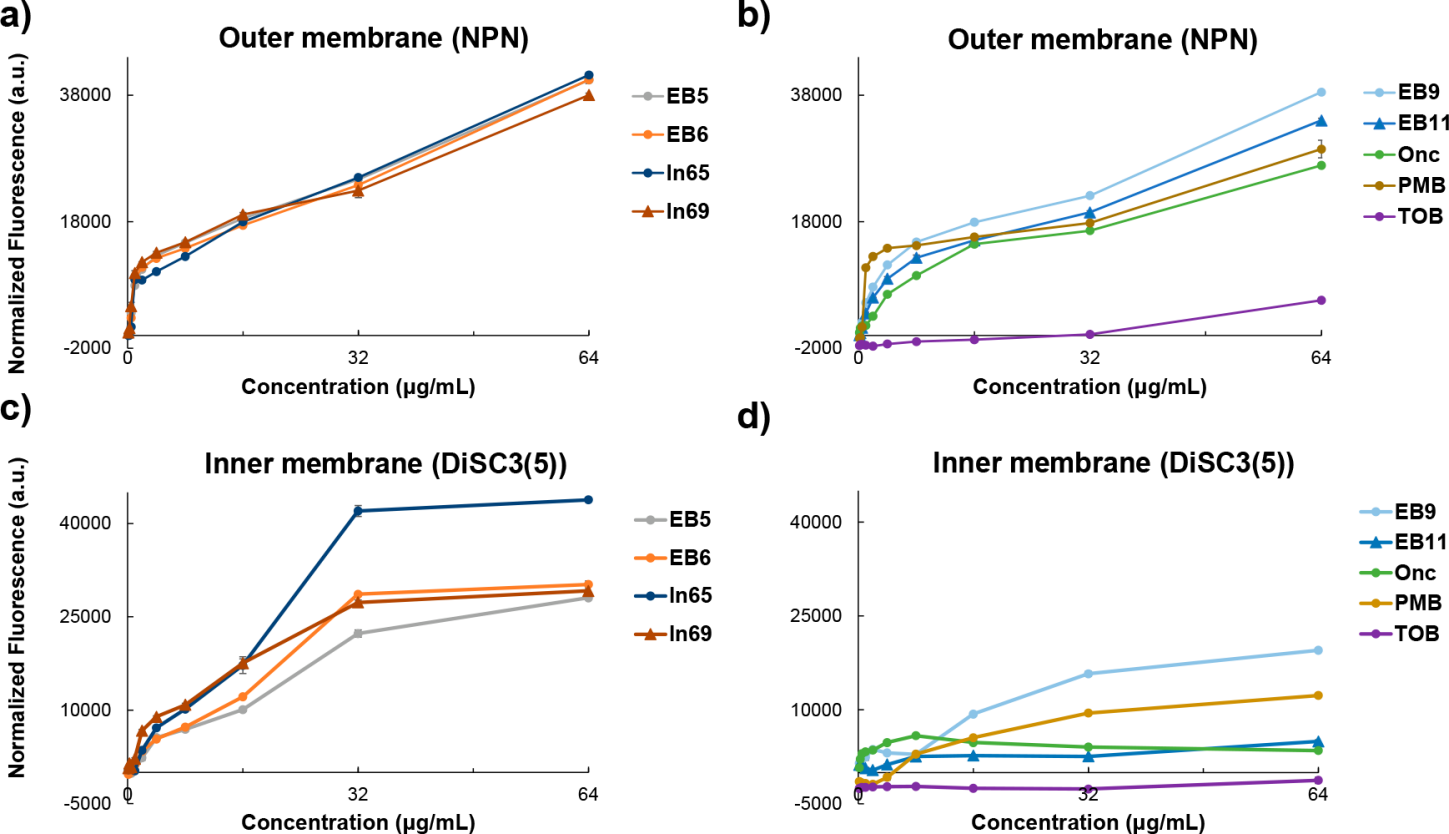
Interactions of peptide–peptoid hybrids with bacterial outer and inner membranes of *E. coli*. (a, b) NPN outer membrane permeability assay. *E. coli* W3110 bacteria were treated with increasing compound concentrations in the presence of 10 μM NPN. The fluorescence intensity (λ_ex_ = 340 nm, λ_em_ = 415 nm) was measured within 5 min. Data represent mean ± SD, *n* = 3. (c, d) DiSC3(5) inner membrane depolarization assay. *E. coli* W3110 bacteria were treated with increasing compound concentrations in the presence of 2 μM DiSC3(5). The fluorescence intensity (λ_ex_ = 610 nm, λ_em_ = 660 nm) was measured within 5 min after treatment, and the data represent mean ± SD, *n* = 3.

**Figure 5:**
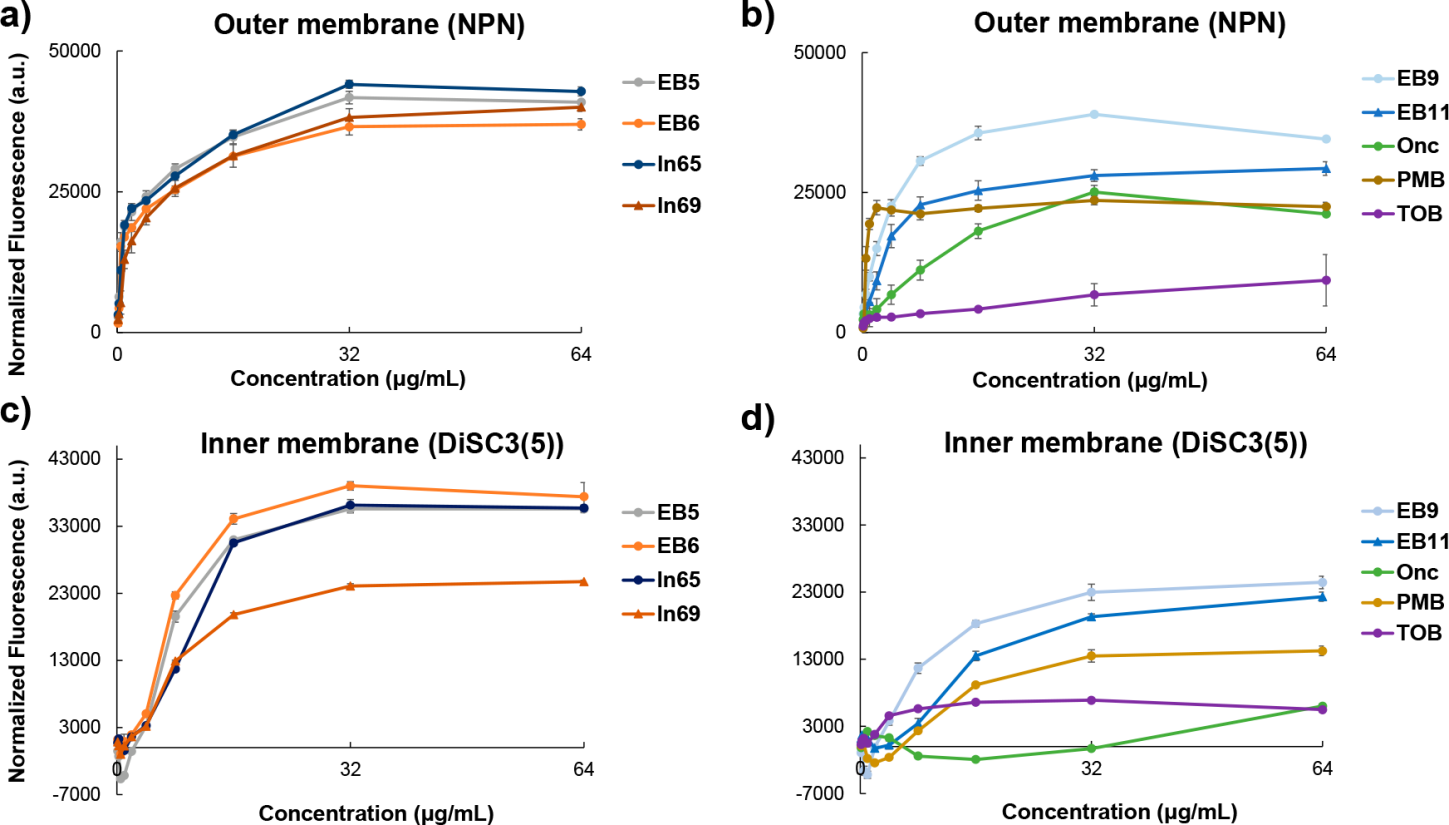
Interactions of peptide–peptoid hybrids with bacterial outer and inner membranes of *P. aeruginosa*. (a, b) NPN outer membrane permeability assay. *P. aeruginosa* PAO1 bacteria were treated with increasing compound concentrations in the presence of 10 μM NPN. The fluorescence intensity (λ_ex_ = 340 nm, λ_em_ = 415 nm) was measured within 5 min. Data represent the mean ± SD, *n* = 3. (c, d) DiSC3(5) inner membrane depolarization assay. *P. aeruginosa* PAO1 cells were treated with increasing compound concentrations in the presence of 2 μM DiSC3(5). The fluorescence intensity (λ_ex_ = 610 nm, λ_em_ = 660 nm) was measured within 5 min after treatment, and the data represent mean ± SD, *n* = 3.

Circular dichroism (CD) spectra of **EB1**, **EB5**, and **EB6**, which were antibacterial in full MH, indicated a significant α-helix content in the presence of 5 mM dodecyl phosphocholine (DPC) or 20% trifluoroethanol (TFE) as folding inducers as typically observed with α-helical AMPs, showing that these compounds were able to form α-helical and presumably amphiphilic and membrane-disruptive conformations (Figure 3a–c). Indeed, vesicle leakage assays showed very strong activities on fluorescein-loaded vesicles made of the anionic lipid egg yolk phosphatidyl glycerol (EYPG) mimicking the bacterial membrane (Figure 2c and S2). These compounds also showed measurable although very low levels of activity on vesicles made of egg yolk phosphatidyl choline (EYPG) mimicking eukaryotic membranes, in line with their weak hemolytic properties. These data suggested that they might act by a membrane-disruptive mechanism like the parent AMPs **ln65** and **ln69**. Indeed, a fluorescence assay with *N*-phenylnaphthylamine (NPN)^66^ showed that **EB5** and **EB6** permeabilized the outer membrane of *E. coli* and *P. aeruginosa* cells to a similar extent as **ln65** and **ln69** (Figure 4a and 5a). Furthermore, fluorescence assay with (3,3′-dipropylthiadicarbocyanine (DiSC3(5))^67^ showed that the compounds also strongly depolarized the inner membrane similarly to the parent AMPs (Figure 4c and 5c).

**Figure 6:**
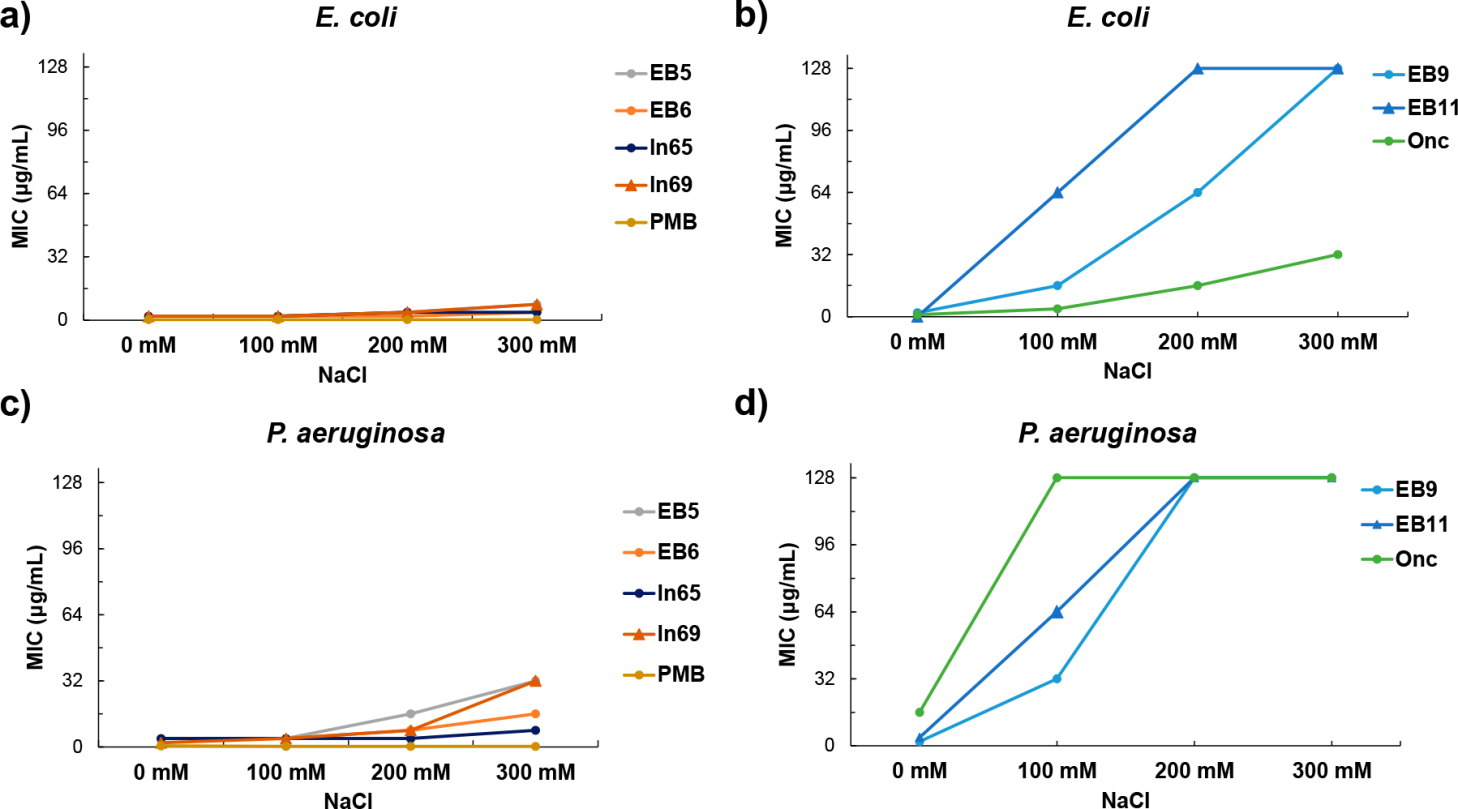
Antibacterial effects of peptide–peptoid hybrids as a function of NaCl concentration in diluted Mueller–Hinton broth (12.5% MH) at pH 8.5 against (a, b) *E. coli* W3110 and (c, d) *P. aeruginosa* PAO1. Values represent two different duplicates MIC determinations.

By contrast, CD spectra of the remaining peptoids such as **EB9**, **EB10**, and **EB11**, which were inactive in full MH, nonhemolytic, but were antibacterial in dilute MH, only had very low or no α-helix content, suggesting a disordered conformation (Figure 3d–f). These compounds displayed weak or no vesicle leakage activity on EYPG vesicles and no activity on EYPC vesicles, showing that they did not have membrane-disruptive activity (Figure 2c and S2). Nevertheless, the NPN assay showed significant outer membrane permeabilization for **EB9** and **EB11**, and both compounds also partly depolarized the inner membrane as indicated by the DiSC3(5) assay, although to a lesser extent (Figures 4b, d and 5b, d) but substantially higher than the aminoglycoside antibiotic tobramycin (**TOB**) used as a negative control. As it is known that monovalent cations can interfere in the binding of AMPs with bacterial membrane and NaCl is the most abundant salt *in vivo*,^64,68−71^ we address the activity of our compound under various NaCl concentrations. The activities of **EB9** and **EB11** as well as of the PrAMP **Onc** were strongly reduced in the presence of high salt concentration (up to 300 mM NaCl), while the membrane-disruptive compounds and **PMB** were less affected (Figure 6).

### Transmission Electron Microscopy

**Figure 7:**
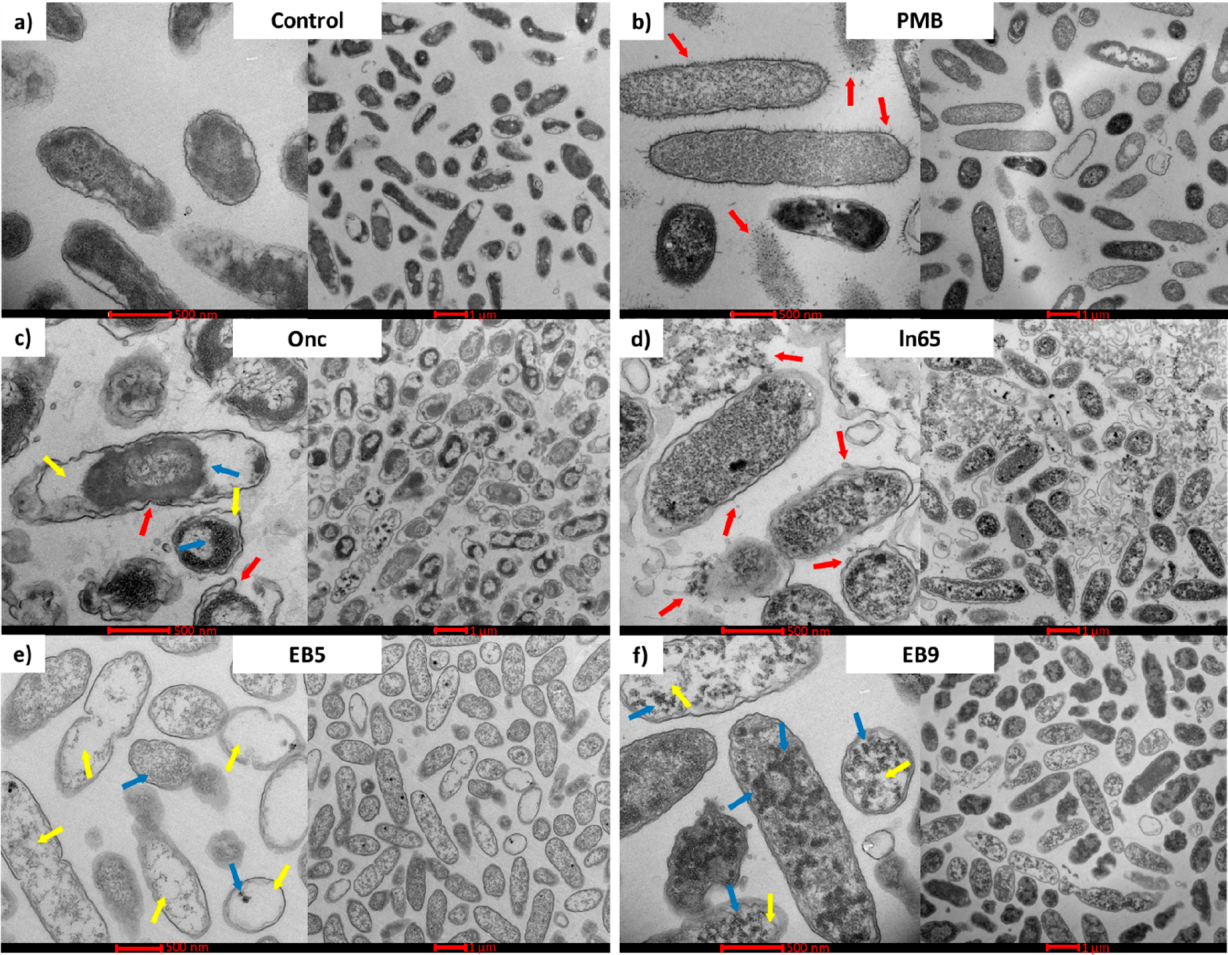
TEM micrographs of *P. aeruginosa* PAO1 (OD_600_ = 0.5), taken after treatment (10 × MIC) in 12.5% MH at pH 8.5 and incubation (2 h at 37 °C) for (a) untreated control, (b) the cyclic peptide antibiotic polymyxin B, (c) the PrAMP oncocin, (d) AMP **ln65**, (e) peptide–peptoid hybrid **EB5**, (f) peptide–peptoid hybrid **EB9**. Scale bars are 500 nm and 1 μm. Red arrows: membrane perturbations. Blue arrows: intracellular aggregation. Yellow arrows: empty area within the cell.

To look for intracellular changes in *P. aeruginosa* PAO1, we compared the modifications induced by our mixed peptide–peptoids **EB5** and **EB9** (inactive on EYPG vesicles) with the cyclic peptide antibiotic **PMB**, the PrAMP **Onc**, and our reference AMP **ln65** by transmission electron microscopy (TEM). Images of the control, nontreated bacteria showed very distinct bacterial membranes and well dispersed intracellular components visible as darker, more electron-dense zones (Figure 7a). By contrast, bacteria exposed to **PMB** showed blebbing and a disrupted outer membrane, which is typical for this membrane-targeting antibiotic (red arrows, Figure 7b), and those treated with the ribosome-targeting **Onc** showed aggregation of intracellular components, with large empty spaces within the cells, as well as some partial disruption of their outer membrane (blue, yellow, and red arrows, Figure 7c). Furthermore, the micrographs of cells treated with our reference AMP **ln65** were consistent with strong membrane-disruptive activity, as indicated by empty cells and cytosolic material floating around the sample, with widespread peeling of the outer membrane and many fragmented bacteria with blebs (red arrows, Figure 7d).

TEM images of cells treated with peptide–peptoid hybrid **EB5**, which was active on EYPG vesicles (Figure 2c), showed much milder disruptions compared to cells treated with the parent AMP **ln65**. Indeed, the outer bacterial membrane of cells treated with **EB5** was not broken, but the bacteria were emptied of intracellular components with few aggregated cellular contents. These images suggest a membrane permeabilization effect of **EB5** that might be mediated by pore formation (yellow and blue arrows, Figure 7e). On the other hand, cells treated with **EB9**, which was inactive on EYPG vesicles (Figure 2c), showed aggregation of intracellular components as the major effect, with asymmetric repartitions inside the cells (blue and yellow arrows, Figure 7f), an effect very similar to that previously reported for peptoids.^43^

## Discussion

We originally discovered the amphiphilic, α-helical undecapeptide **ln65** in a combinatorial library. Its sequence, composed only of leucines and lysines, did not occur, even as partial sequence, in databases of known peptides and proteins.^13^ Surprisingly, the α-helical conformation of **ln65** and associated membrane-disruptive antibacterial activities were preserved in several diastereomers such as **ln69** containing four d-lysines.^13,59^ Inspired by many reports on antimicrobial peptoids with tunable helicity and toxicity,^29−40^ here we investigated if **ln65** might similarly tolerate the presence of peptoid units in its sequence. We investigated antibacterial effects in full MH medium, as well as in dilute (12.5% MH) medium, conditions believed to be closer to actual infections,^60^ and under which conditions the proline-rich AMP oncocin shows its activity.^46,47,49,50^

In full MH medium, three of the eleven peptide–peptoid hybrids investigated (**EB1**, **EB5**, and **EB6**) exhibited strong antibacterial effects against almost all bacterial species including multidrug-resistant clinical isolates as well as rapid time–kill kinetics, to an extent comparable to the parent AMP **ln65**. These hybrids showed significant α-helical content in their CD spectra and strong leakage activities on anionic EYPG vesicles, which suggest that their antibacterial activities are mediated by membrane disruption induced by amphiphilic and partly α-helical conformers. Indeed, NPN and DiSC3(5) assays showed that these compounds permeabilized the bacterial outer membrane and depolarized the bacterial inner membrane of *E. coli* and *P. aeruginosa* cells to a similar extent as **ln65** and **ln69**. These membrane-disruptive peptide–peptoid hybrids showed somewhat lower hemolysis and HEK293 cell toxicity and much better serum stability compared with the parent l-peptide **ln65**. The effects on PAO1 cells treated with **EB5** as observed by TEM were milder than those induced by **ln65**, also indicating a reduced or at least different interaction of **EB5** with membranes compared to **ln65**.

In dilute medium (12.5% MH), we found that most peptide–peptoid hybrids, including **EB2** and **EB3** containing as few as three peptoid units, the peptoid–peptide alternating sequence **EB9**, and the full peptoid **EB11**, showed significant antibacterial effects against at least two bacterial species, an effect that was enhanced under slightly alkaline conditions (pH 8.5). Time–kill kinetics were slower than for **ln65** and comparable to those of the PrAMP oncocin. Strikingly, those peptide–peptoid hybrids which were only active in dilute MH did not show any α-helical content by CD, or membrane-disruptive activities in vesicle leakage assays, and were entirely nonhemolytic and nontoxic to HEK293 cells. Detailed investigations with **EB9** and **EB11** indicated significant interactions with the bacterial outer and inner membrane as indicated by NPN and DiSC3(5) assays, suggesting that the compounds can internalize into bacteria. Indeed, TEM micrographs of bacterial cells exposed to **EB9** showed the aggregation of intracellular components as the major effect, without visible membrane disruption. This intracellular action is comparable to that reported for several nonmembrane-disruptive peptoids.^42−45^

The experimental evidence of α-helical content provided by CD spectra and associated with membrane-disruptive effects suggests the existence of folded, amphiphilic conformers in peptide–peptoid hybrids **EB1**, **EB5**, and **EB6**. By contrast, the antibacterial but nonmembrane-disruptive peptide–peptoid hybrids do not show any evidence for α-helical folding by CD and are probably conformationally disordered.

**Figure 8:**
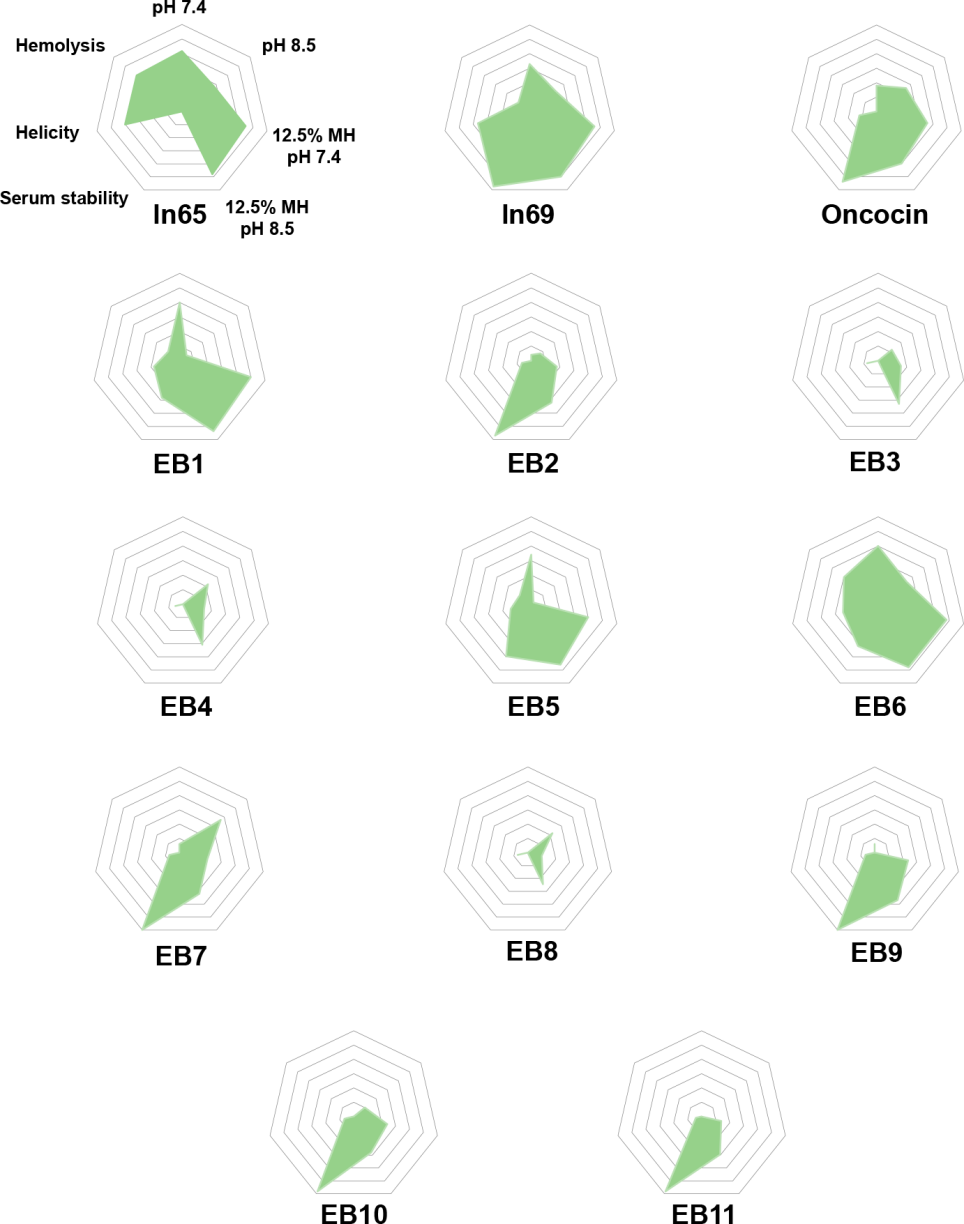
Overview of the observed activities of the compounds. A more distant point to the center describes a higher value, classified from 0 to 6. “pH 7.4” and “pH 8.5” represent the MIC values, ranging from > 32 μg/mL (= 0) to < 0.123 (= 6) that were measured in full MH at the respective pH. “12.5% MH pH 7.4” and “12.5% MH pH 8.5” represent the MIC values, ranging from > 32 μg/mL to < 0.123 that were measured in diluted MH at the respective pH. “Serum stability” and “Helicity” both express percentages, of undegraded peptide in human serum after 24 h and α-helicity in presence of 5 mM DPC, respectively (0% = 0 and 100% = 6). “Hemolysis” ranges from > 2000 μg/mL to 31.25 μg/mL (with > 2000 μg/mL = 0 and 31.25 μg/mL = 6).

A comparative overview of the observed activities can be obtained in the form of radar plots displaying average antibacterial effects under the different conditions, serum stability, α-helicity, and hemolysis activities (Figure 8). The activity patterns of the membrane-disruptive hybrids **EB1**, **EB5**, and **EB6** resemble diastereomer **ln69**; however, **ln69** remains the best compound in terms of high antibacterial effects and serum stability combined with low hemolysis and toxicity. On the other hand, the nonmembrane-disruptive hybrids such as **EB2**, **EB9**, **EB10**, and **EB11** resemble the PrAMP oncocin. Although the activities observed with these non-membrane-disruptive hybrids must be considered rather weak since they only appear in dilute MH, the absence of hemolysis and toxicity is appealing and provides further evidence that peptoids represent a privileged structural class for antibacterial development.

## Conclusion

The experiments above with peptide–peptoid hybrids derived from the α-helical membrane-disruptive undecapeptide **ln65** show that α-helicity and membrane-disruptive effects can be preserved upon substitution of up to five residues for peptoid units, as in **EB5**. On the other hand, hybrids containing as few as three peptoid units such as **EB2**, as well as the alternating sequence **EB9**, or the full peptoid **EB11**, lack α-helicity and membrane-disruptive effects but surprisingly display potent antibacterial effects against multiple bacteria when tested under dilute medium conditions under which PrAMPs such as oncocin show their activity. These nonmembrane-disruptive peptide–peptoid hybrids seem to act on intracellular targets, as supported by TEM imaging. The possibility to abolish membrane-disruptive effects and enable intracellular targeting by introducing only a few peptoid units comes with the added benefit of enhanced serum stability and lower cell toxicity and might be generally applicable to design nontoxic antimicrobial peptides.

## Methods

### Peptide Synthesis

Reagents and LC/MS analytical procedures for our standard peptide synthesis have been detailed in earlier publications.^21,64^

#### Synthesis of Peptide–Peptoid Hybrids

Linear mixed peptide–peptoids were synthesized manually following the standard 9-fluorenylmethoxycarbonyl (Fmoc) solid-phase peptide synthesis procedures as well as the submonomer method for the insertion of the corresponding *N*-substituted glycines building blocks.^26^ The syntheses were performed at 60 °C under nitrogen bubbling. All peptide syntheses were carried out by using Rink Amide LL resin (100–200 mesh), unloaded (0.29 mmol/g), and on a 0.116 mmol scale (400 mg of resin). The resin was swollen in DMF for 30 min at 60 °C and deprotected twice, 1 and 4 min, respectively, using a deprotection cocktail containing piperazine (5%), DBU (2%), and butanol (10%) in DMF, at 60 °C.

#### Coupling of Fmoc-Amino Acids

5 equiv. of Fmoc-protected amino acid with a concentration of 0.2 M together with 5 equiv. of OxymaPure and 6 equiv. of DIC, both with a concentration of 0.2 M, were used as coupling reagents in 4.5 mL of DMF. The reaction was stirred for 8 min at 60 °C, washed 2 times with 6 mL of DMF, and a second coupling step was performed under the same conditions. The resin was then washed 3 times with 6 mL of DMF. After each standard amino acids coupling, the Fmoc protecting group was removed with 2 sets of 6 mL of a deprotection cocktail containing piperazine (5%), DBU (2%), and butanol (10%) in DMF, respectively, for 1 and 4 min at 60 °C. The resin was then washed 4 times with DMF (6 mL).

#### Coupling of Peptoid Residues

13 equiv. of bromoacetic acid, with a concentration of 0.5 M together with 6 equiv. of DIC, with a concentration of 0.2 M were stirred at 60 °C for 8 min in 5 mL of DMF. After washing the resin 2 times with 6 mL of DMF, 5 equiv. of the corresponding primary amine, with a concentration of 0.2 M, was stirred for 8 min at 60 °C. The resin was then washed 3 times with 6 mL of DMF. The final cleavage was carried out by treating the resin with 7 mL of a TFA/TIS/H_2_O (94:5:1, v/v/v) solution for 3 h at room temperature. The peptide solution was then precipitated with 25 mL of *tert-*butyl methyl ester (TBME), centrifuged for 10 min at 4000 rpm (twice), evaporated, and dried with argon. The dried crude product was dissolved in a water/acetonitrile mixture, filtered (pore size 0.45 μm), and purified by preparative RP-HPLC with gradients of 15 min. The pure fraction was analyzed by LC-MS with a 5 min gradient. Pure products were obtained as white foamy solids after lyophilization. The yields were calculated for the TFA salts.

### Antimicrobial Activity (MIC)

Antimicrobial activity was assayed for all the peptide–peptoids against *P. aeruginosa* PAO1, *K. pneumoniae* NCTC418, *E. coli* W3110, *A. baumannii* ATCC 19606, methicillin-resistant *S. aureus* COL (MRSA), and for selected peptides on *P. aeruginosa* PA14 and the polymyxin-B-resistant derivatives PA14 4.13, PA14 4.18, PA14 2P4, as well as the clinical isolates ZEM-1A and ZEM9A, *K. pneumoniae* OXA-48, *S. aureus* Newman, *Enterobacter cloacae*, *Stenotrophomonas maltophilia*, *Burkholderia cenocepacia*, and *Staphylococus epidermidis*. To determine the Minimal Inhibitory Concentration (MIC), the Broth Microdilution method was used.^72^ A colony of bacteria was picked and grown in LB medium overnight at 37 °C. The next day, the culture was then regrown in LB medium to log phase (OD_600_ = 0.6 to 0.8), which lasted approximately 4 h, and diluted to an OD_600_ of 0.022 in the desired medium (full MH or 12.5% MH).

The compounds were prepared as stock solutions of 2 mg/mL in sterilized Milli-Q deionized water and then diluted in the desired media (full MH or 12.5% MH) to reach the first concentration tested (32 μg/mL). The compounds were added to the first well of 96-well sterile, round-bottom microtiter plates in polypropylene (Costar, untreated) and diluted serially by 1/2 in the desired media (full MH or 12.5% MH), to a final volume of 150 μL/well. To each well was finally added 4 μL of the diluted bacterial suspension (see above), corresponding to approximately 5 × 10^5^ CFU. For each test, two columns of the plate were kept for sterility control (medium only), growth control (medium with bacterial inoculum, no compound). The positive control, Polymyxin B (starting with a concentration of 16 μg/mL) in MH medium with bacterial inocula, was introduced in the two lines of the plate. The plates were incubated at 37 °C for ca. 18 h under static conditions. Next, 15 μL of 3-(4,5-dimethylthiazol-2-yl)-2,5-diphenyltetrazolium bromide (MTT)^73^ (1 mg/mL in sterilized MilliQ deionized water) were added to each well, and the plates were incubated for 20–30 min at room temperature. The minimal inhibitory concentration (MIC) was defined as the lowest concentration of the compound that inhibits the visible growth of the tested bacteria (yellow) with the unaided eye.

### Bacteria Growth Curves

A single colony of *P. aeruginosa* PAO1, *K. pneumoniae* NCTC418, *E. coli* W3110, *A. baumannii* ATCC 19606, and *S. aureus* COL (MRSA strain) was picked and grown overnight with shaking (180 rpm) in 5 mL of LB (Sigma Aldrich, Buchs, Switzerland) medium overnight at 37 °C. The overnight bacterial culture was diluted to OD_600_ of 0.002 (2 × 10^6^ CFU/mL) in fresh, diluted, or full MH (Sigma Aldrich, Buchs, Switzerland, full media at pH 7.4 and 12.5% at pH 8.5) medium. 100 μL of the prepared bacteria solution in MH and 100 μL of the corresponding MH (full or diluted) were mixed in 96-well microtiter plates (TPP, untreated, Corning Incorporated, Kennebunk, USA). The 96-well microtiter plates were incubated at 37 °C with shaking (180 rpm). Bacteria were quantified at 0, 1, 2, 3, 4, 5, and 7 h by plating 10-fold dilutions of the sample in sterilized normal saline (NaCl 0.9%) on LB agar plates. LB agar plates were incubated at 37 °C for 14–16 h, and the number of individual colonies was counted at each time-point. The assay was performed twice in triplicate.

### Further Assays

Time–killing assay, hemolysis activity assays (MHC), lipid vesicle leakage assays, serum stability assays, and circular dichroism spectra recording were carried out as described in earlier publications.^21,64^

### Cell Viability Assay

#### Cell Culture Conditions

HEK293 cells were cultured and maintained in DMEM high glucose (Dulbecco’s modified Eagle medium, Sigma Aldrich) medium supplemented with 10% FBS (Sigma Aldrich) and 1% penicillin/streptomycin. A549 cells were cultured and maintained in RPMI-1640 medium (Sigma Aldrich), supplemented with 10% FBS (Sigma Aldrich) and 1% penicillin/streptomycin. The cells were handled and subcultured according to the manufacturer instructions. Cells were incubated in a humidified incubator at 37 °C in the presence of 5% CO_2_.

#### Cell Viability Assay by Alamar Blue

Assays on HEK293, A549 cells were performed as described earlier.^74^

### DiSC3(5) Inner-Membrane Depolarization Assay

A single colony of *P. aeruginosa* PAO1 and *E. coli* W3110 was grown overnight with shaking (150 rpm) in LB broth (5 mL) at 37 °C. A 100 μL portion of the overnight culture was regrown in 10 mL of LB broth with shaking (200 rpm) to the exponential phase (OD_600_ = 1, corresponding to 10^9^ CFU/mL). Bacteria were washed once with HEPES buffer (5 mM HEPES, 5 mM glucose, pH 7.4) and diluted to an OD_600_ = 0.5. Stock solution of 10 mM of 3,3′-dipropylthiadicarbocyanine iodide (DiSC3(5), purchased from Sigma) was prepared in DMSO. Stock solutions of 2 mg/mL of the compounds were prepared in sterilized milli-Q water and diluted to the beginning concentration of 128 μg/mL in 200 μL of HEPES buffer containing 4 μM of the fluorescent probe DiSC3(5). The diluted samples were added to the first well of 96-well plates (black wells, flat bottom, BRAND GmbH Wertheim, Germany) and diluted serially by 1/2. 100 μL of the bacterial suspension in HEPES buffer (without fluorophore) was added to each well. In this case, the final OD of bacteria was 0.25, the final concentration of peptide in the first column was 64 μg/mL, and DiSC3(5) was 2 μM. The control wells are buffer containing DiSC3(5) and bacterial suspension containing DiSC3(5) in HEPES buffer. The plate was measured with a Tecan instrument Infinite M1000 within 5 min. The plate was enabled to shake for 5 s before measurement. The excitation wavelength used was 610 ± 5 nm, and the emission wavelength was 660 ± 5 nm. The assay was measured in triplicate and repeated at least two times.^65,75^

### NPN Outer-Membrane Permeabilization Assay

A single colony of *P. aeruginosa* PAO1 and *E. coli* W3110 was grown overnight while being shaken (150 rpm) in LB-broth (5 mL) at 37 °C. 100 μL of the overnight culture was regrown in 10 mL of LB broth with shaking (200 rpm) to the exponential phase (OD_600_ = 1, corresponding to 10^9^ CFU/mL). Bacteria were washed three times with HEPES buffer (5 mM HEPES, 5 mM glucose, pH 7.4), but not diluted yet to their final concentration. 200 μL of peptide samples was added to the first well of 96-well plates (black wells, flat bottom, BRAND GmbH Wertheim, Germany), 100 μL of HEPES/glucose buffer with 10 μM *N*-phenyl-1-naphthylamine (NPN, purchased from Acros Organics) was added in all the wells, and the compounds were diluted serially by 1/2 (add a control with a final volume of 200 μL with 10 μM NPN in buffer only). Ca. 10 min before the measurement and the final dilution of the bacteria, the plates and the 10 μM NPN in HEPES/glucose buffer (for bacteria dilution) were incubated at 37 °C (prior to the dilution of the bacteria with buffer containing NPN 10 μM). Later, the bacteria were diluted to OD_600_ = 0.5 with the NPN in HEPES/glucose buffer. 100 μL of the bacterial suspension was added to each well. In this case, the final OD of bacteria was 0.25, the final concentration of the desired compound 64 μg/mL, and NPN 10 μM. The control wells are buffer containing NPN (10 μM, 200 μL) and bacterial suspension containing NPN in HEPES buffer. The plate was measured with a Tecan instrument Infinite M1000 within 5 min. The plate was enabled to shake for 5 s before measurement. The excitation wavelength used was 340 ± 5 nm, and emission wavelength was 415 ± 5 nm. The assay was repeated at least three times.

### Transmission Electron Microscopy (TEM)

Transmission electron microscopy was evaluated at a high cell density (10^8^ CFU) and a concentration (10× MIC) that killed the bacteria but not all of them. An overnight culture of *P. aeruginosa* PAO1 was regrown until exponential phase (1 mL, OD_600_ = 0.5) in 12.5% MH medium pH 8.5, treated with **PMB**, **Onc**, **ln65**, **EB5**, and **EB9**, in diluted MH medium (at pH 8.5) and incubated for 2 h at 37 °C. Just before the samples were further prepared for the microscopy, surviving bacteria were quantified by plating 10-fold dilutions of samples in sterilized normal saline (NaCl 0.9%) on LB agar plates. LB agar plates were incubated at 37 °C for 14–16 h, and the number of individual colonies was counted at each time-point (bacterial count, Table S4), to demonstrate partial bacteria killing. The assay was performed in triplicate. Each bacteria sample was centrifuged at 12,000 rpm for 3 min, and the supernatant was discarded. The samples were then further processed and imaged using our previously described protocol.^64^

## Supporting Information Available

Peptide yields, MS and analytical data, CD spectra and analysis, vesicle leakage fluorescence curves, serum stability curves, full cytotoxicity data, bacteria growth curves and HPLC-MS chromatograms and HRMS spectra for all compounds (PDF)
SMILES and activities of all tested compounds (CSV)
