# Supplementary material for: Antimicrobial Peptide–Peptoid Hybrids with and without Membrane Disruption
Source: ACS Infect Dis. 2023 Nov 21;9(12):2593–606. doi: 10.1021/acsinfecdis.3c00421 (PMC10714400; doi:10.1021/acsinfecdis.3c00421)
Supplement: Supplementary file 1 — id3c00421_si_001.pdf [file id3c00421_si_001.pdf]

Supporting Information for:

Antimicrobial peptide-peptoid hybrids with and without membrane disruption

Etienne Bonvin,[1] Hippolyte Personne,[1] Thierry Paschoud,[1] Jérémie Reusser,[1] Bee Ha Gan,[1] Alexandre Luscher,[2,3] Thilo Köhler,[2,3] Christian van Delden[2,3] and Jean-Louis Reymond[1,*]

[1]*Department of Chemistry, Biochemistry and Pharmaceutical Sciences, University of Bern, Freiestrasse 3, CH-3012 Bern, Switzerland, e-mail: jean-louis.reymond@unibe.ch*

[2]*Department of Microbiology and Molecular Medicine, University of Geneva, CH-1211 Geneva, Switzerland*

[3]*Service of Infectious Diseases, University Hospital of Geneva, CH-1211 Geneva, Switzerland*

Contents

1. Peptide Synthesis ... 2
2. Activities ... 3
3. Bacteria Growth ... 5
4. Lipid Vesicle Leakage Assay ... 8
5. Serum Stability Assay ... 11
6. Circular Dichroism Spectroscopy ... 12
7. Cell Viability Assay ... 14
8. Transmission electron microscopy (TEM) ... 17
9. HPLC and MS data ... 18

1. Peptide Synthesis

Table S1: Synthesis and Analytical data for peptides and peptide-peptoids.

Cpd	Sequence [a]	SPPS Yield [b] mg (%)	MS Analysis [c] calc./obs. (g/mol)	Analytical HPLC [d] t_R (min)
**Oncocin**	VDKPPYLPRPRPPRRIYNR	111.0 (34)	2389.3767/2389.3857	2.36
**In65**	KKLLKLLKLLL	84.0 (58)	1321.9948/1322.0021	3.63
**In69**	kkLLkLLkLLL	102.6 (71)	1321.9948/1322.0027	3.23
**EB1**	*k*KLLKLLKLL*l*	77.4 (38)	1321.9948/1321.9937	3.50
**EB2**	*k*KLLKLL*k*LL*l*	69.0 (34)	1321.9948/1321.9995	3.40
**EB3**	KKLLKLLK*lll*	54.1 (26)	1321.9948/1322.0023	3.23
**EB4**	KKLLK*llklll*	38.0 (18)	1321.9948/1322.0014	3.30
**EB5**	*kkllk*LLKLLL	61.0 (30)	1321.9948/1322.0019	3.50
**EB6**	*kk*LL*k*LLKLLL	77.5 (38)	1321.9948/1321.9995	3.58
**EB7**	*kk*LL*k*LL*k*LLL	18.4 (9)	1321.9948/1322.0017	3.16
**EB8**	KK*ll*K*ll*K*lll*	36.9 (18)	1321.9948/1322.0028	3.44
**EB9**	K*k*L*l*K*l*L*k*L*l*L	35.6 (17)	1321.9948/1322.0020	3.31
**EB10**	*k*K*l*L*k*L*l*K*l*L*l*	55.6 (27)	1320.9948/1320.9960	3.49
**EB11**	*kkllkllklll*	22.8 (11)	1321.9948/1322.0032	3.44

[a] One letter code for amino acids, k = D-lysine, *k* = *N*Lys (lysine-like residue), *l* = *N*Leu (leucine-like residue). [b] Yield given for RP-HPLC purified product. [c] Electrospray ionization mass spectrometry (positive mode), the calculated monoisotopic masses, and the observed masses for $[M+H]^+$ are reported. [d] Retention time in analytical RP-HPLC (A/D = 100/0 to 0/100 in 7.0 min, λ = 214 nm).

2. Activities

Table S2: Antimicrobial activities of peptide-peptoid hybrids.

No.	Sequence[a]	MIC[b] (µg/mL)																				MHC[c] (µg/mL)	CD
		E. coli W3110				*P. aeruginosa* PAO1				*A. baumannii* ATCC19606				*K. pneumoniae* NCTC418				*S. aureus* COL (MRSA)					%α[d]
		Full MH		12.5%		Full MH		12.5%		Full MH		12.5%		Full MH		12.5%		Full MH		12.5%			
		7.4	8.5	7.4	8.5	7.4	8.5	7.4	8.5	7.4	8.5	7.4	8.5	7.4	8.5	7.4	8.5	7.4	8.5	7.4	8.5		
**PMB[e]**		0.25	<0.1	0.5	0.25	0.5	0.25	0.5	0.5	0.25	0.25	0.5	0.25	0.25	0.25	0.5	0.5	>64	2	8	1	>2000	n.d.
**Onc[f]**		4	1	1	1	>32	>32	32	16	32	8	4	4	4	4	1	1	>32	>32	32	16	>1000	20/23
**ln65**	KKLLKLLKLLL	4	8	2	2	2-4	16	4	4	2-4	8	4	2	4	8	2-4	2	4	8	2	2	125	73/64
**ln69**	kkLLkLLkLLL	4	16	2	2	8	8	4	2	2-4	8-16	2	2	8	16	4	2	16	16	2	2	1000	61/34
**EB1**	*k*KLLKLLKLL*l*	2	32	2	1	4	16	2	2	2	>32	2	1-2	8	>32	2	1-2	8/32	32	2	2	1000	30/26
**EB2**	*k*KLLKLL*k*LL*l*	32	32	4	2	32	16	16	8	>32	32	16	8	>32	>32	>32	>32	>32	32	32	2	>2000	11/15
**EB3**	KKLLKLLK*lll*	>32	16	4	2-4	>32	16	16	4	>32	>32	32	16	>32	32	>32	16	>32	32	32	8	>2000	13/15
**EB4**	KKLLK*llklll*	>32	8	8	2	>32	18	8-16	4	>32	16	32	16	>32	16	>32	32	>32	16	32	4-8	>2000	9/11
**EB5**	*kkllk*LLKLLL	2	>32	2	2	4	>32	4	4	2	>32	2	2	>32	16-32	16	4	8	>32	4	2	1000	24/25
**EB6**	*kk*LL*k*LLKLLL	2	8	2	2	4	16	2	4	2	8	2	2	8	8-16	4	2	8	16	2	2	250	41/35
**EB7**	*kk*LL*k*LL*k*LLL	16	4	4	1	32	8	16	8	>32	4	16	8	>32	4	32	32	>32	4-8	16	8	>2000	12/13
**EB8**	KK*ll*K*ll*K*lll*	>32	8	8	2-4	>32	16	>32	8	>32	16	32	16	>32	16	>32	>32	>32	16	32	4-8	>2000	11/15
**EB9**	K*k*L*l*K*l*L*k*L*l*L	16	>32	2	2	32	>32	4	2	>32	>32	16	8	>32	>32	>32	16	>32	>32	32	4-8	>2000	11/13
**EB10**	*k*K*l*L*k*L*l*K*l*L*l*	>32	32	2	2-4	>32	>32	4	4	>32	>32	16	8-16	>32	>32	>32	32	>32	32	32	8	>2000	11/11
**EB11**	*kkllkllklll*	>32	>32	8	2-4	>32	>32	8	4	>32	>32	16	16	>32	>32	32	32	>32	>32	>32	4-8	>2000	7/8

[a] One letter code for amino acids. The D-amino acids are denoted with the small letters and *N*-substituted residues are indicated in italics; k = D-lysine, *k* = *N*Lys (lysine-like residue), *l* = *N*Leu (leucine-like residue). [b] Minimum inhibitory concentration (MIC), in µg/mL, was determined on bacteria in Mueller-Hinton broth (MH) at pH 7.4 after incubation for 16-20 hours at 37 °C. Values represent two different triplicate MIC determinations. [c] Minimum Hemolytic Concentration (MHC) measured on human red blood cells (hRBC) in 10 mM phosphate buffer, 150 mM NaCl, pH 7.4, 25 °C, 4 h. [d] CD spectra were recorded at 0.1 mg/mL in aqueous 10 mM phosphate buffer at pH 7.4 with the addition of 5 mM DPC/20% TFE. The primary CD spectra were analyzed using DichroWeb, and the percentages of α-helical signals were extracted. Every building block (peptoid or amino acid) was taken in account for the calculations. The Contin-LL method and reference set 4 were used. [e] Polymyxin B. [f] Oncocin. "n.d." = not determined.

Table S3. Activities against an extended panel of MDR bacteria.

MIC[a] / Cpd	*P. aeruginosa* PA14	PA14 4.13	PA14 4.18	PA14 2P4	ZEM-1A	ZEM9A	*E. cloacae*	*K. pneumoniae* OXA-48	*S. maltophilia*	*S. aureus* Newman	*S. epidermis*
**PMB**[b]	0.25	0.5	0.25	1	1	1-2	1-2	1-2	2	2	2
**Onc**[c]	2	2	2	2	2	16	2	1	> 32	1	4
**ln65**	2	2	2-4	2-4	4	4	4	4	4	2	1
**ln69**	2-4	2-4	4	4	2	2	2	2	2	2-4	1
**EB1**	2	2	2	2	n.d	n.d	n.d	n.d	n.d	2	n.d
**EB2**	2-4	4	4-8	8	n.d	n.d	n.d	n.d	n.d	2-4	n.d
**EB3**	4	4-8	8	16	n.d	n.d	n.d	n.d	n.d	4	n.d
**EB4**	4	8	16-32	16	n.d	n.d	n.d	n.d	n.d	8	n.d
**EB5**	2	2	2	2	2	2	4	8	4-8	2	2
**EB6**	2	2	2	2	2-4	2	4	8	2-4	2	2
**EB7**	4	4	8	16	n.d	n.d	n.d	n.d	n.d	8	n.d
**EB8**	8	16-32	32	32	n.d	n.d	n.d	n.d	n.d	8	n.d
**EB9**	2	2	4-8	4	2-4	2	16	32	8	4	2
**EB10**	4	4	8	8	n.d	n.d	n.d	n.d	n.d	8	n.d
**EB11**	2 - 4	4-8	8-16	8-16	2-4	4	16	32	4	4	1

[a] Minimum inhibitory concentration (MIC), in µg/mL, was determined on bacteria in dilute Mueller-Hinton broth (12.5% MH) at pH 8.5 after incubation for 16-20 hours at 37 °C. Values represent two different triplicate MIC determinations. [b] Polymyxin B. [c] Oncocin. "n.d." = not determined.

3. Bacteria Growth

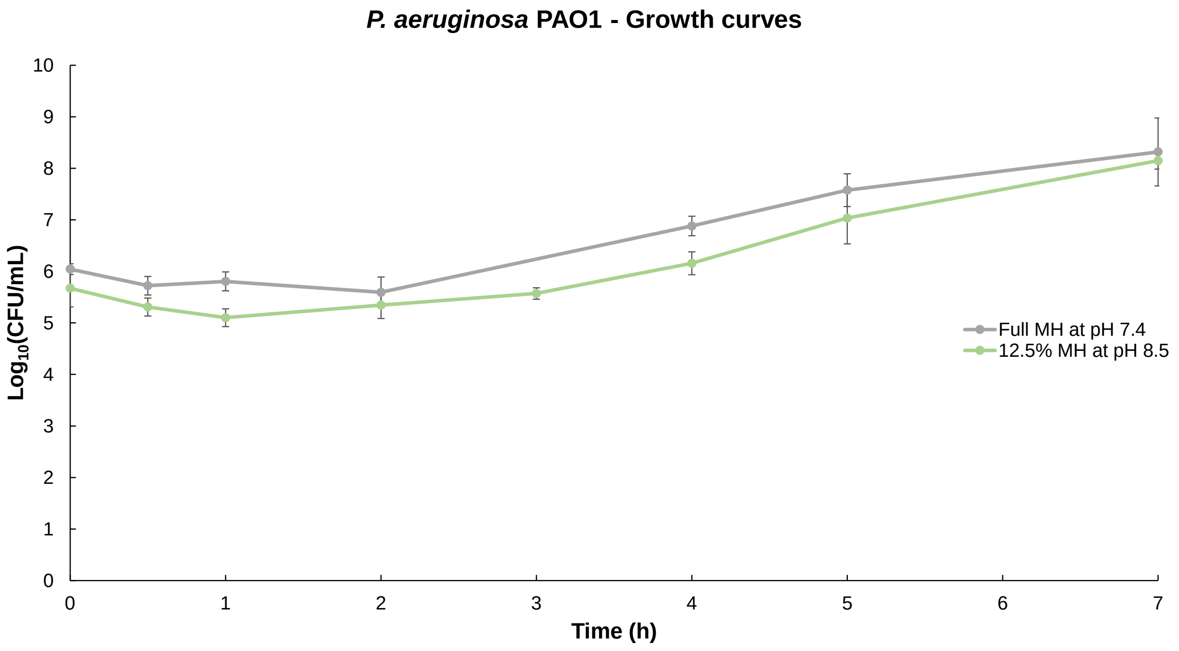

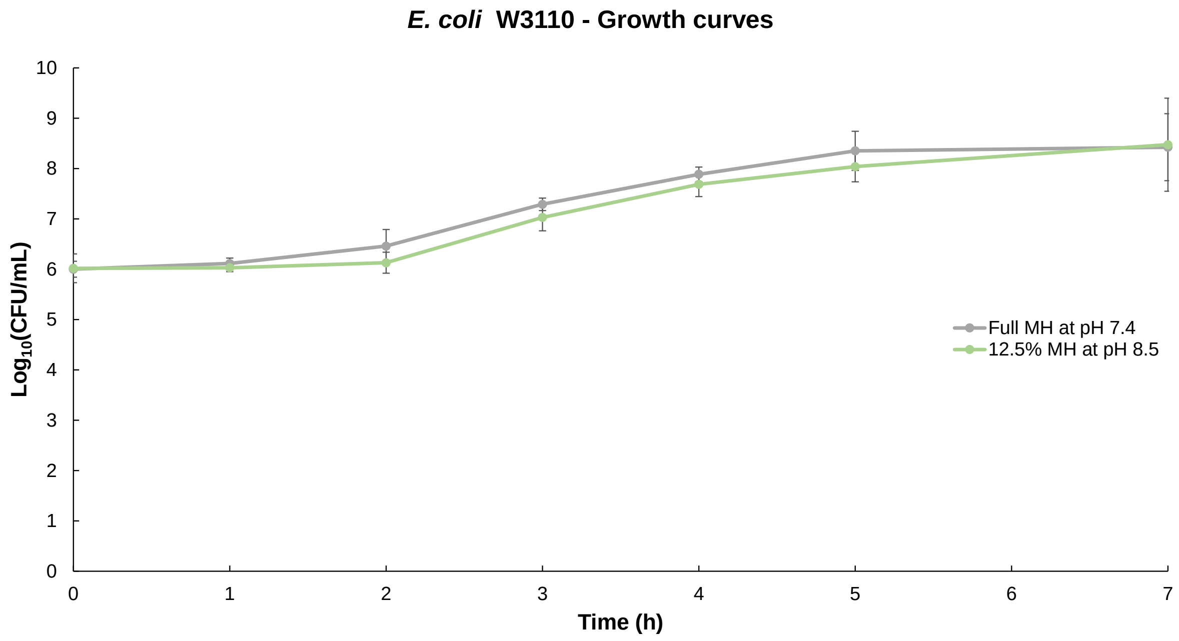

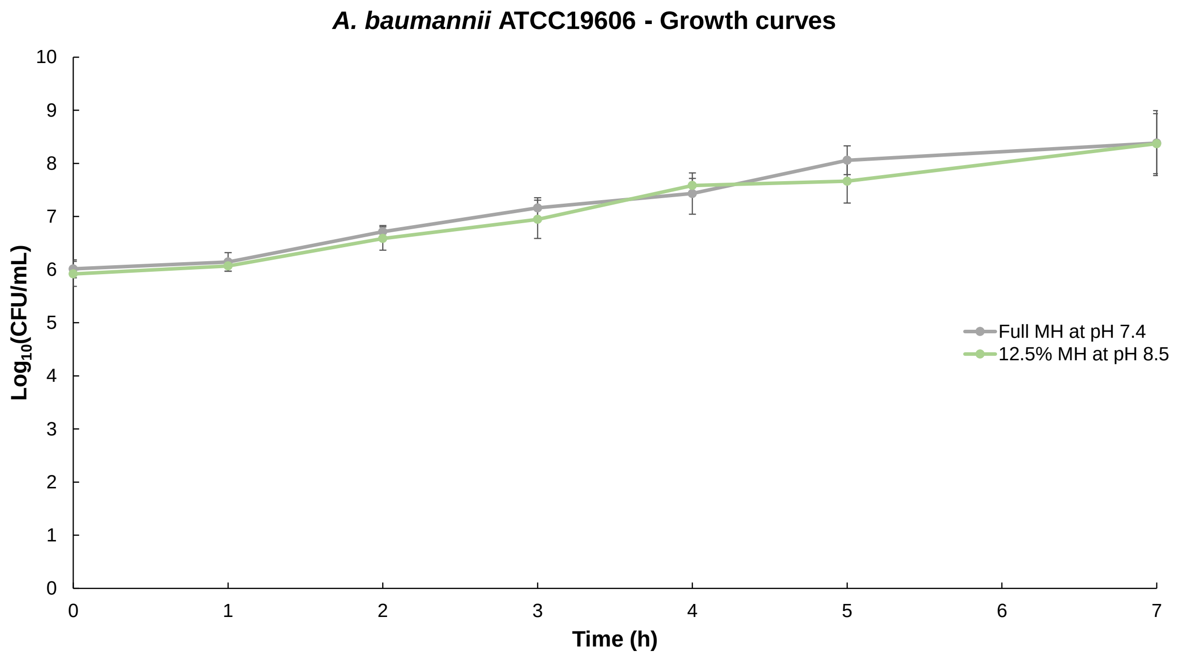
A. baumannii ATCC19606 - Growth curves
Log10(CFU/mL)
10
9
8
7
6
5
4
3
2
1
0
0
1
2
3
4
5
6
7
Time (h)
Full MH at pH 7.4
12.5% MH at pH 8.5

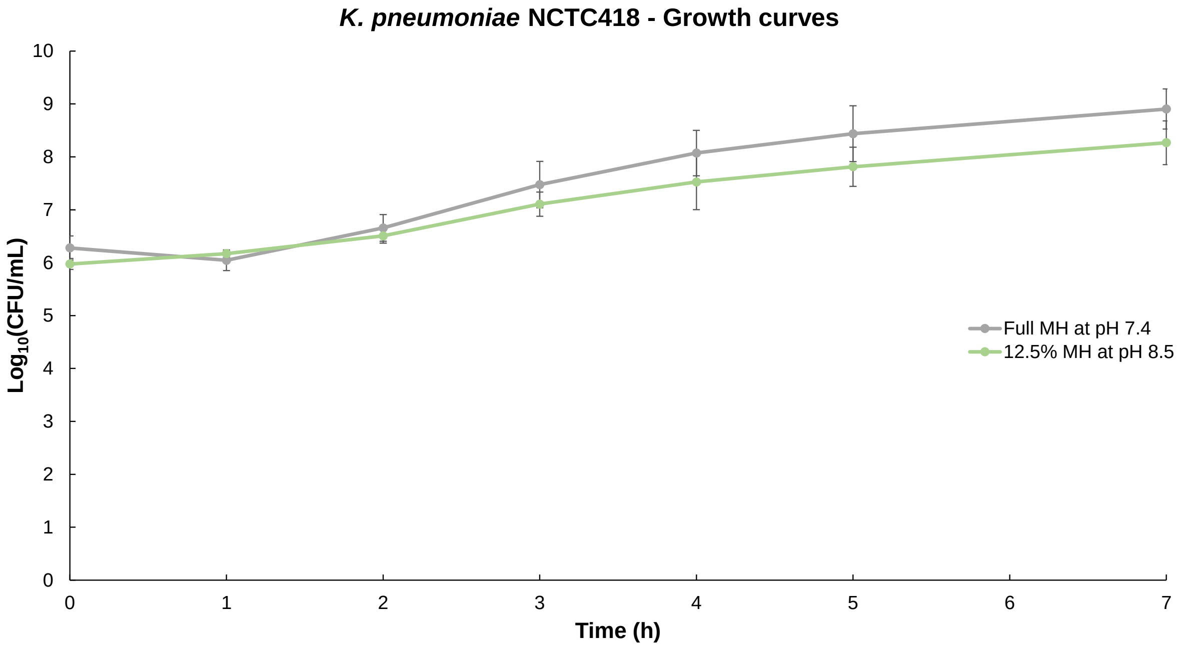
K. pneumoniae NCTC418 - Growth curves
Log10(CFU/mL)
10
9
8
7
6
5
4
3
2
1
0
0
1
2
3
4
5
6
7
Time (h)
Full MH at pH 7.4
12.5% MH at pH 8.5

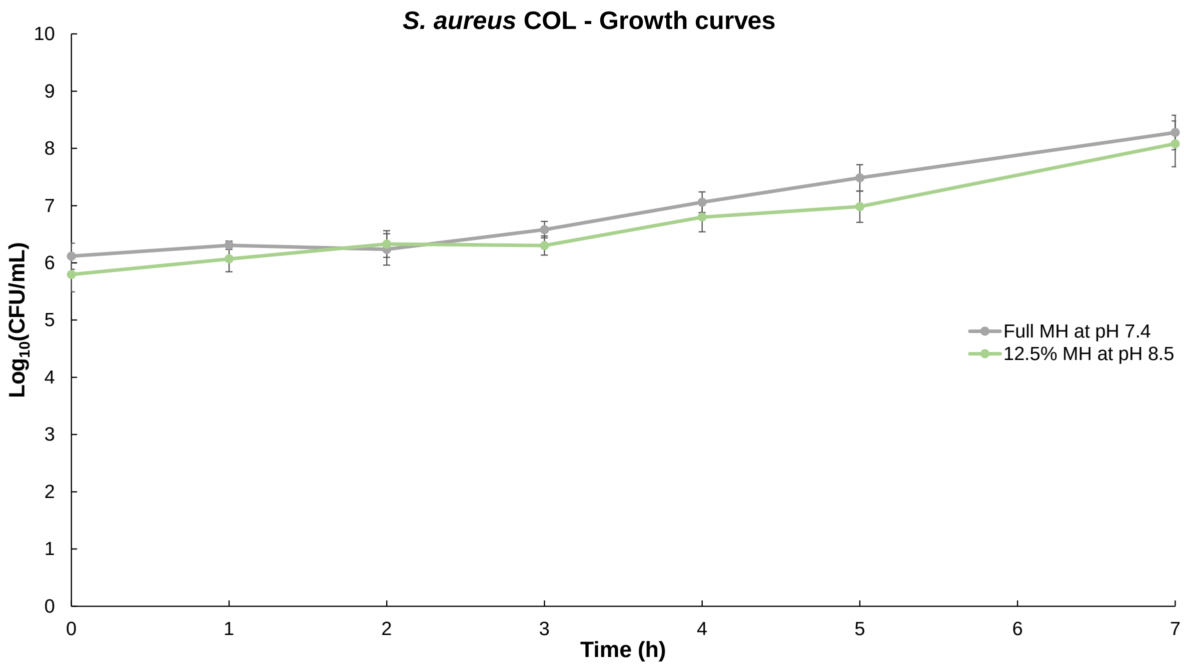

Figure S1: Comparison of the bacterial growth curves in two different conditions, for five strains.

4. Lipid Vesicle Leakage Assay

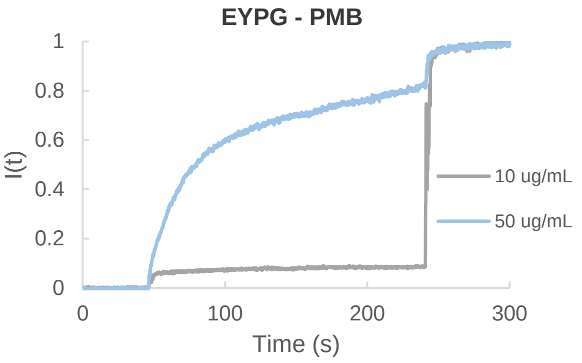

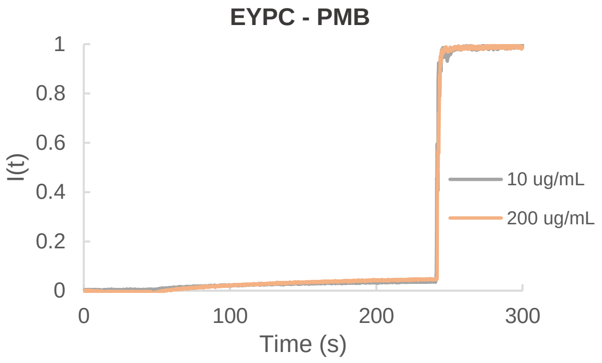

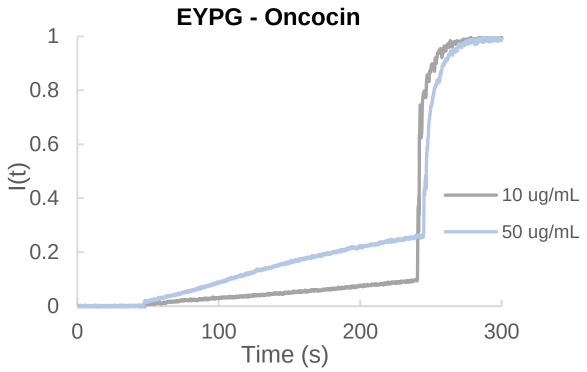

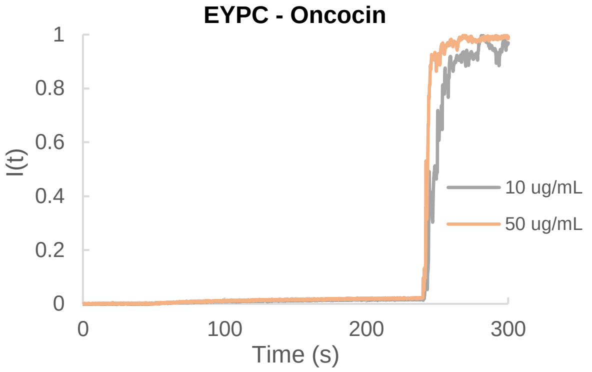

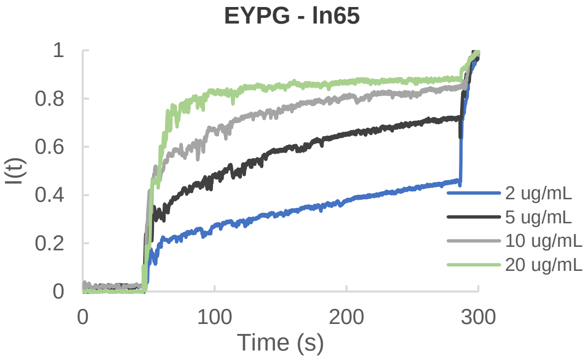

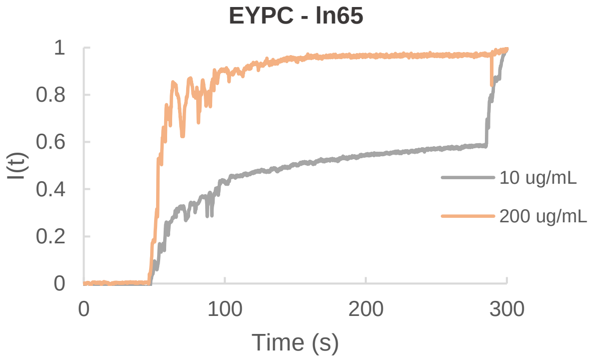

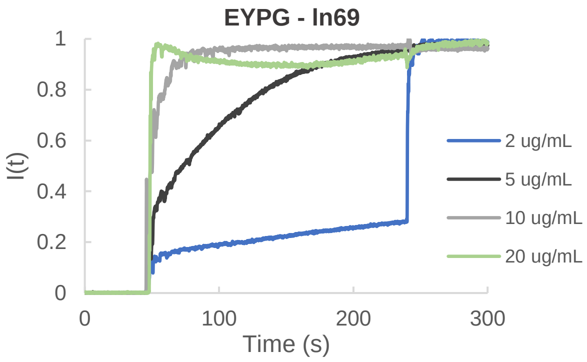

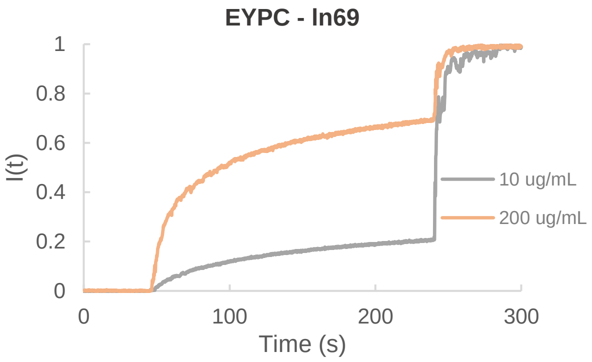

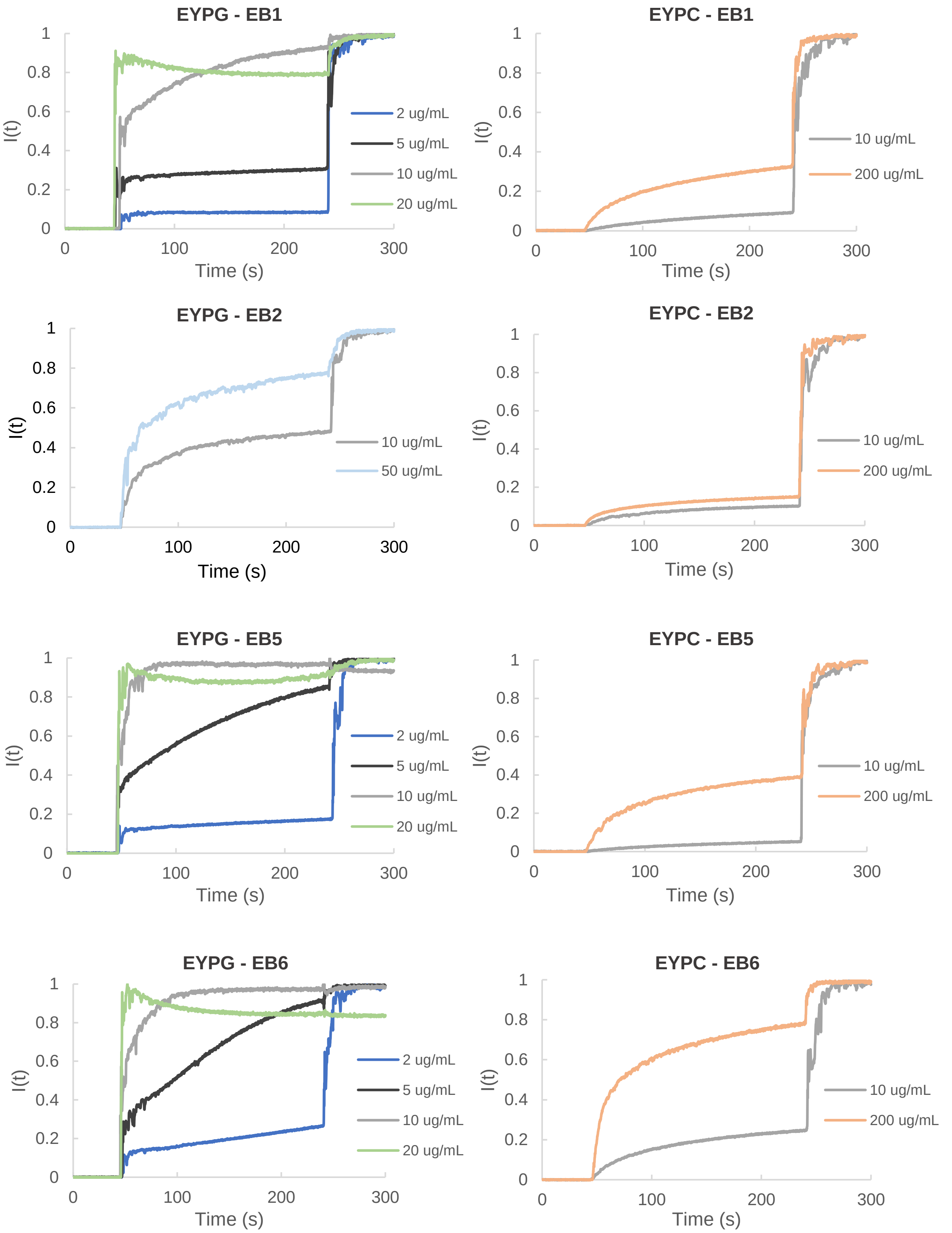
EYPG - EB1
EYPC - EB1
EYPG - EB2
EYPC - EB2
EYPG - EB5
EYPC - EB5
EYPG - EB6
EYPC - EB6
I(t)
Time (s)
2 ug/mL
5 ug/mL
10 ug/mL
20 ug/mL
50 ug/mL
200 ug/mL

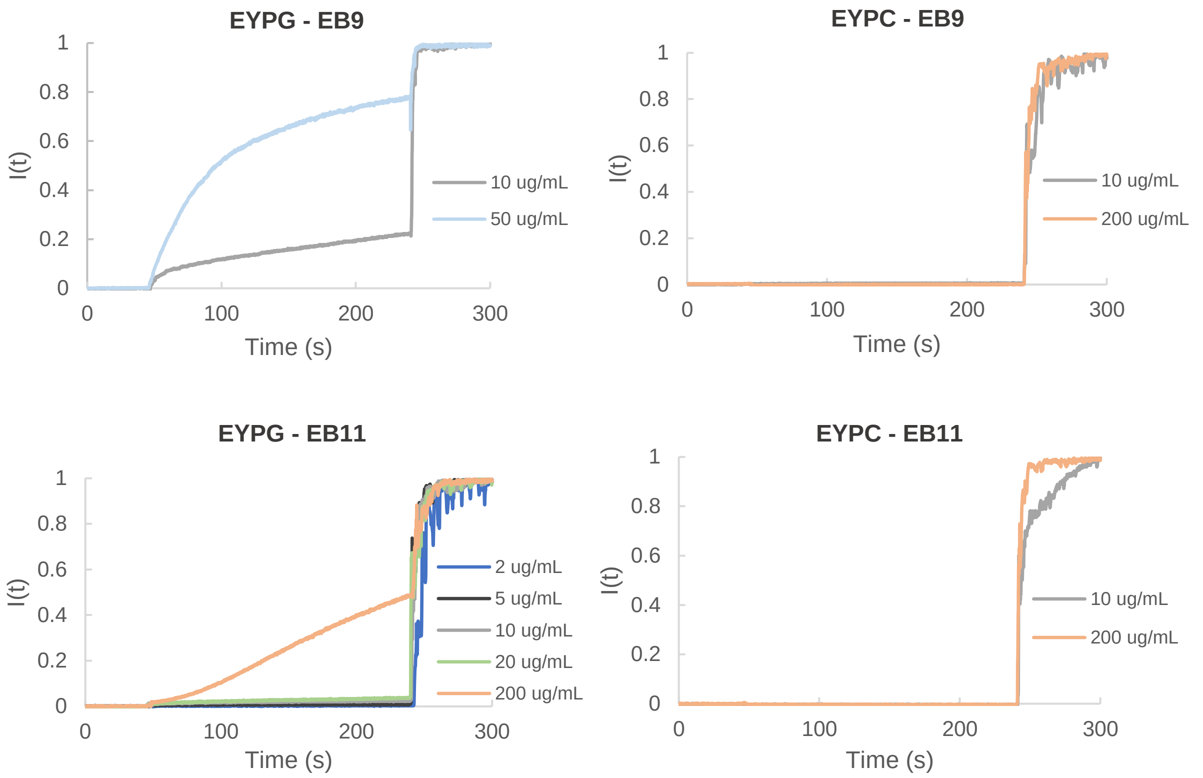

Figure S2: Vesicle leakage experiments using 5(6)-carboxyfluorescein induced by peptides. EYPG and EYPC vesicles were suspended in buffer (10 mM TRIS, 107 mM NaCl, pH 7.4) and the indicated concentration of the compound was added after 45 sec. After 240 seconds, 30 µL Triton X-100 1.2% was added for full release of fluorescein.

5. Serum Stability Assay

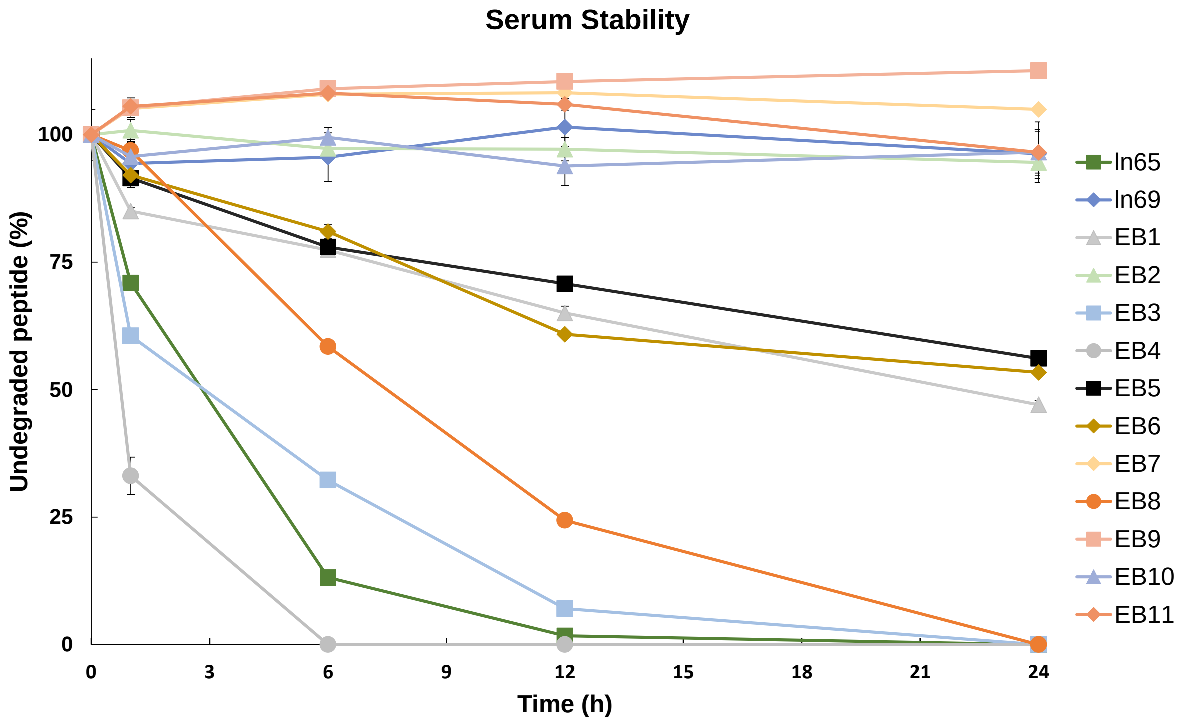

Figure S3: Serum stability assay of the respective compound (200 µM), incubated with human serum (12.5% in TRIS buffer, 0.1 M, pH 7.4) for different times, at 37 °C. Normalized undegraded peptide values determined by RP-HPLC analysis using hydroxybenzoic acid as internal standard. Measured in triplicates and the data represent mean ± SD, n = 3.

6. Circular Dichroism Spectroscopy

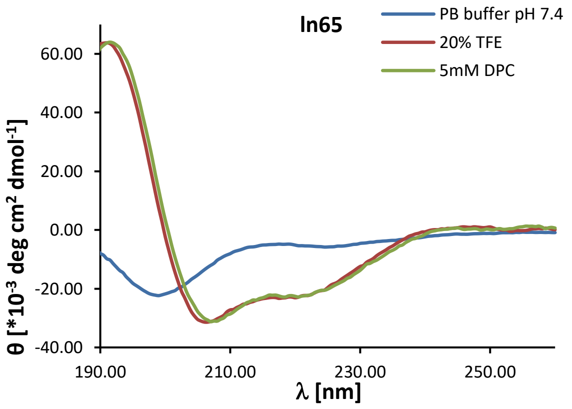

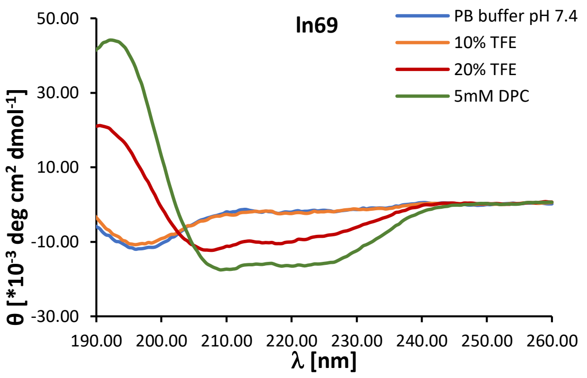

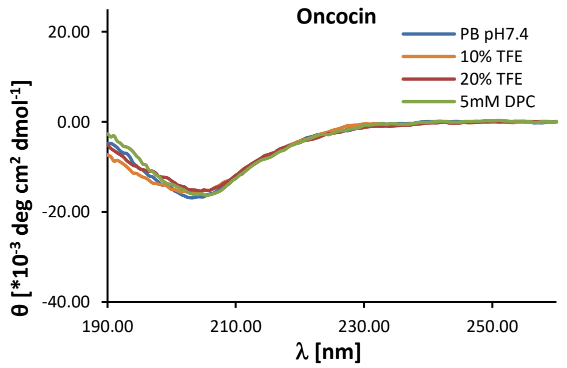

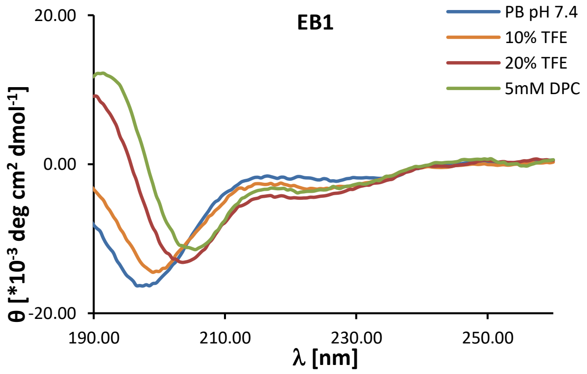

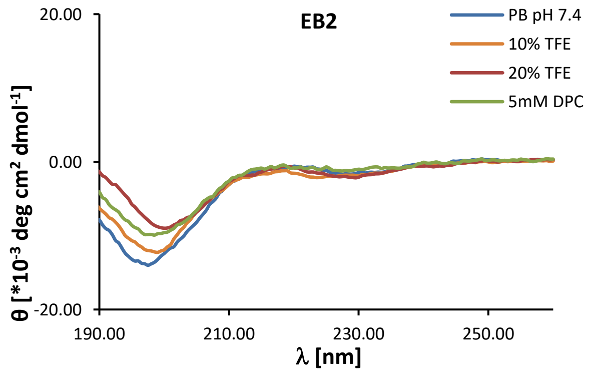

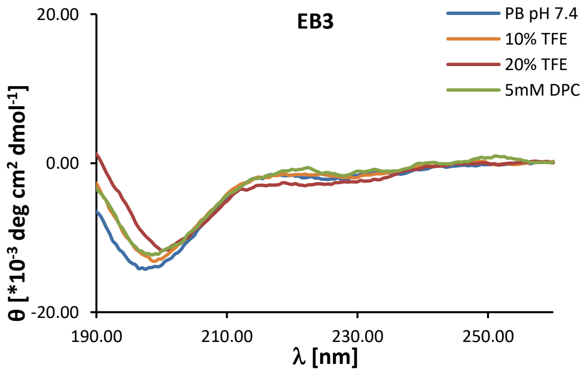

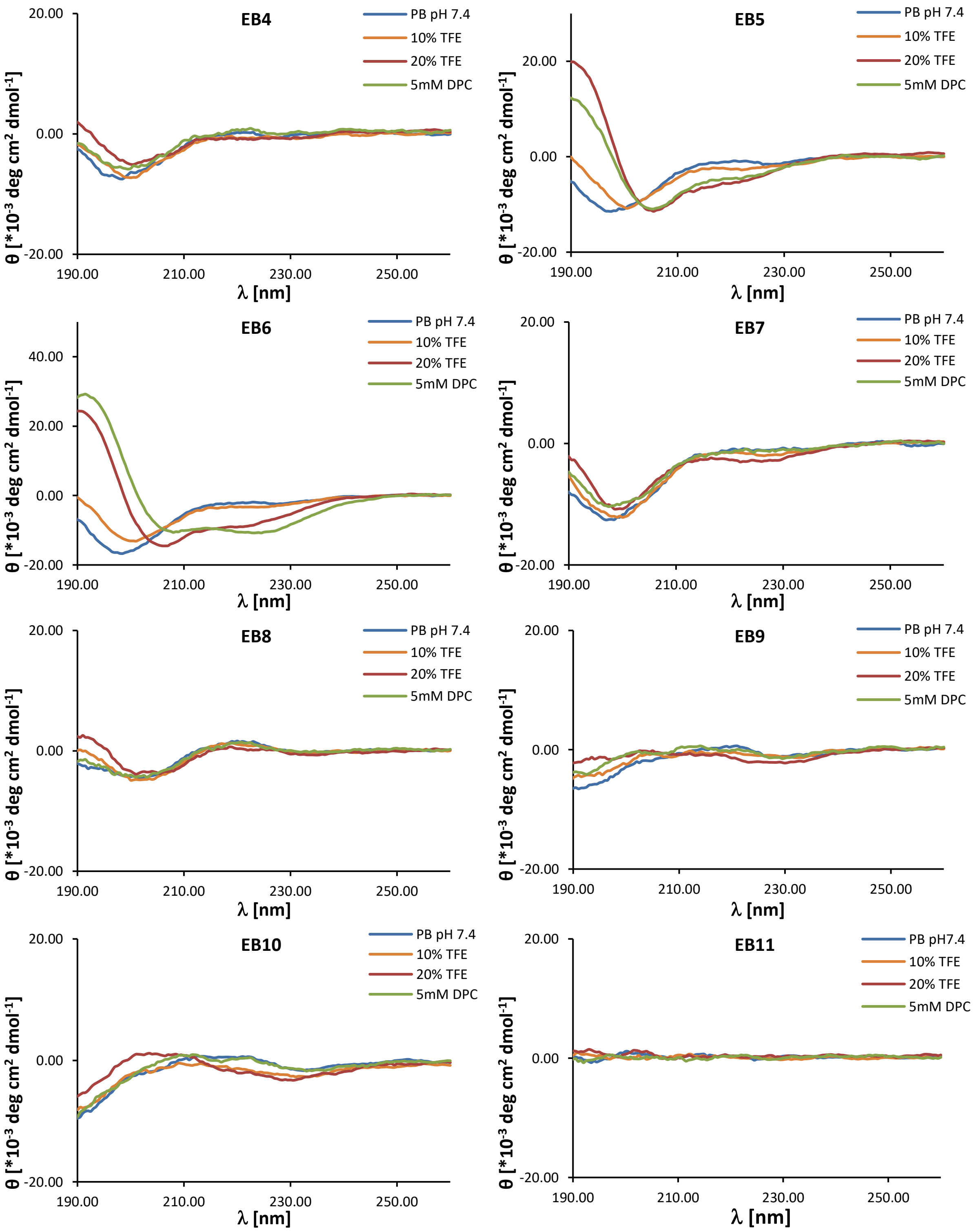

Figure S4: Circular dichroism spectra of the peptides at 100 µg/mL in 7 mM phosphate buffer pH 7.4 (blue line), in presence of different amounts of TFE (10 or 20%, orange and red line respectively) and 5 mM DPC (green line).

7. Cell Viability Assay

				IC_{50} (µM)				
**Cpd**	**In65**	**In69**	**EB1**	**EB2**	**EB5**	**EB6**	**EB9**	**EB11**
**HEK293**	27 ± 6	45 ± 9	110 ± 6	> 200	10 ± 3	54 ± 1	> 200	> 200
**A549**	20 ± 1	21 ± 3	69 ± 2	n.d.	56 ± 2	19 ± 1	n.d.	n.d.

Table S4: Cytotoxicity of selected compounds on HEK293 and A549 cells. All data represented as the IC_{50} value measured by Alamar blue assay after 24 h treatments with concentrations from 0 to 200 µM. The data are represented as the mean value ± SD, n = 3. "n.d." = not determined.

<u>**HEK293 cells:**</u>

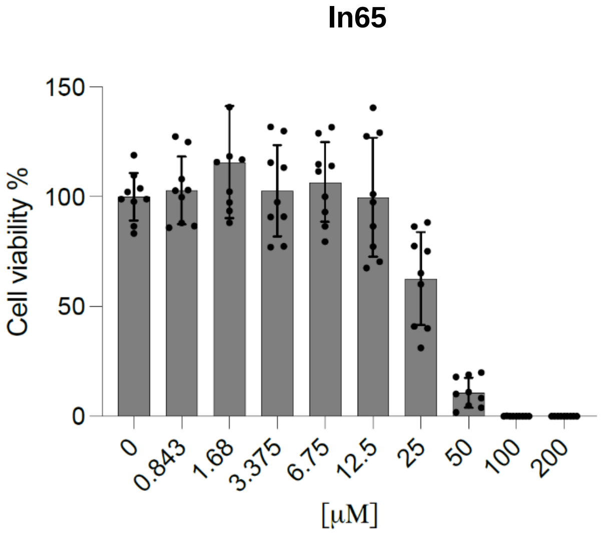

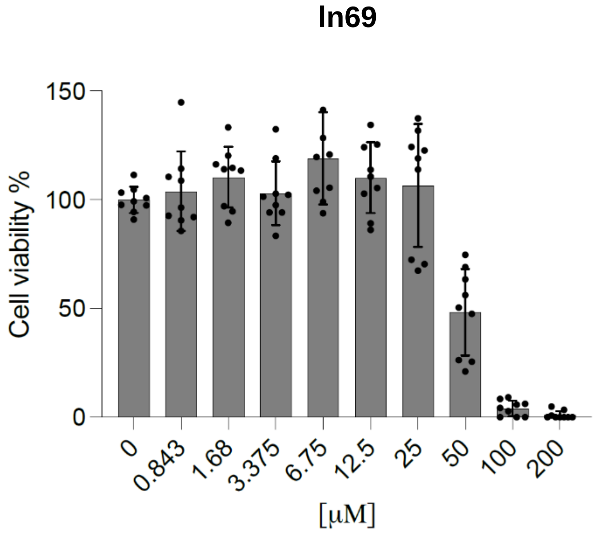

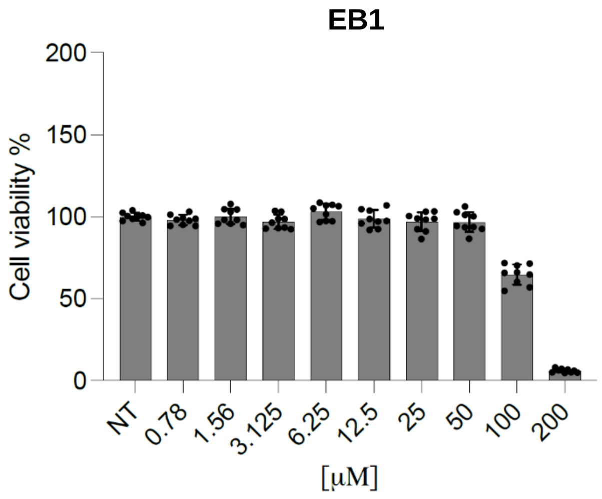

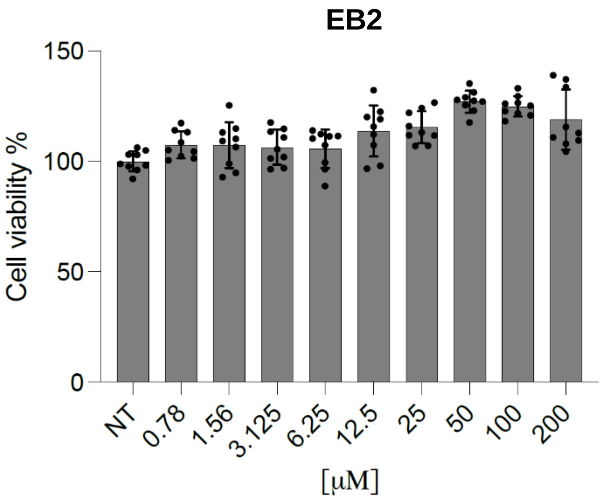

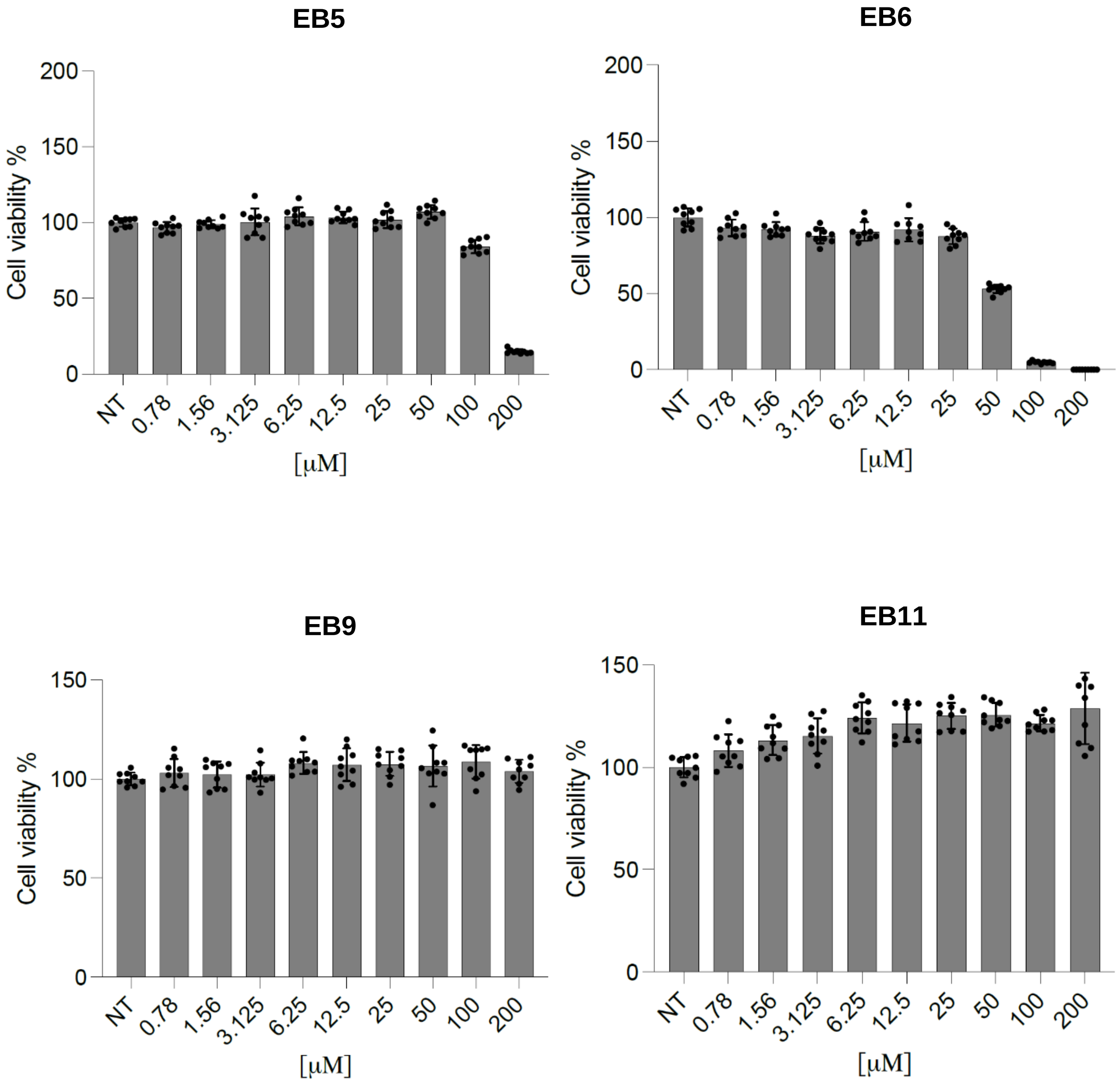

Figure S5: Cytotoxicity of selected compounds on HEK293 cells. The data of three experiments with three replicates per sample were pooled and represented as barplots. The cells were treated with the compounds for 24 hours and their viability was measured with an Alamar Blue assay.

A549 cells:

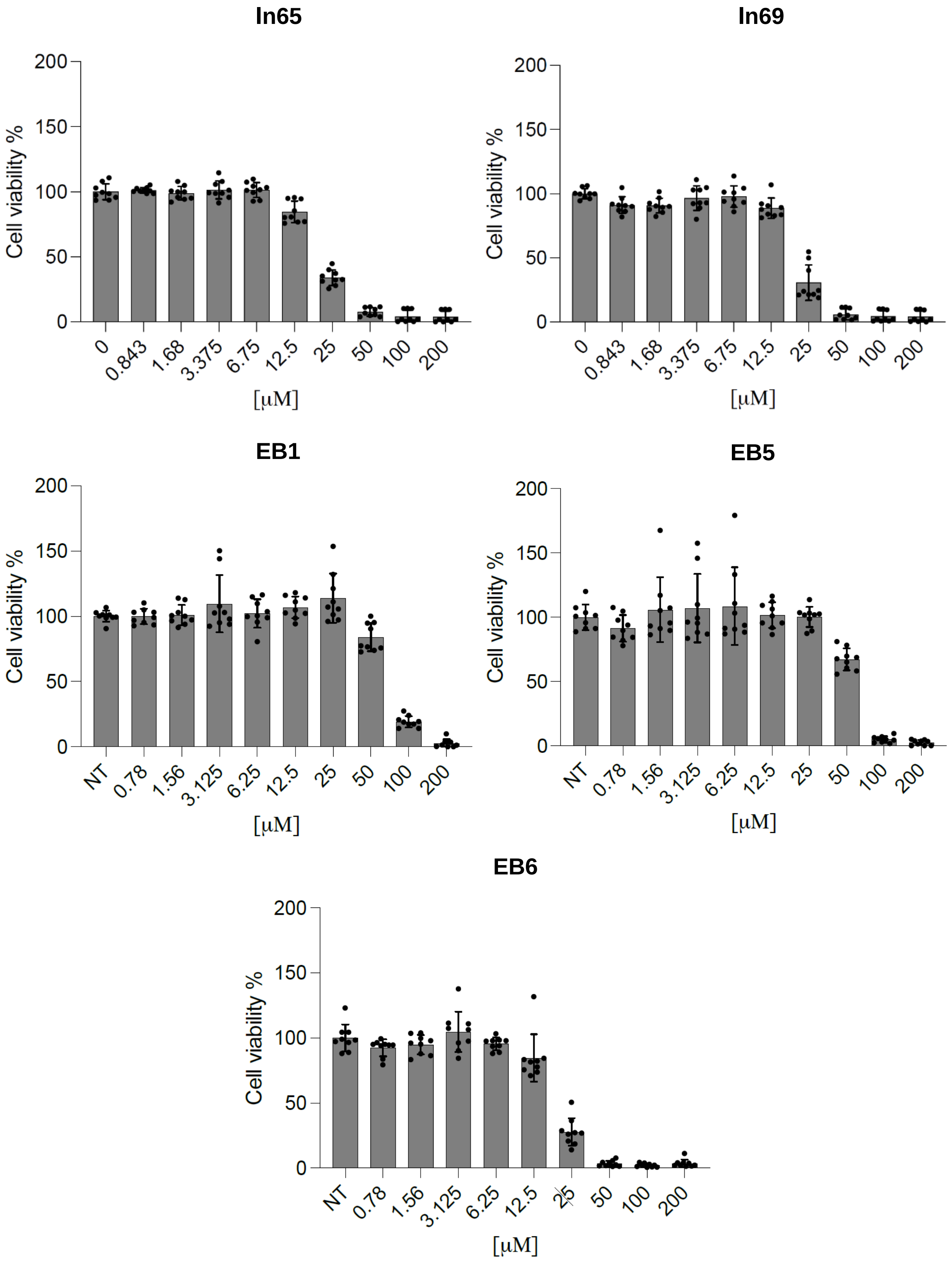

Figure S6: Cytotoxicity of **ln65**, **ln69**, **EB1**, **EB5** and **EB6** on A549 cells. The data of three experiments with three replicates per sample were pooled and represented as barplots. The cells were treated with the compounds for 24 hours and their viability was measured with an Alamar Blue assay.

8. Transmission electron microscopy (TEM)

Table S4: Bacterial counts, 2 hours after treatment, before TEM sample preparation. Data represents triplicates.

	Bacteria count after treatment at 10x MIC and 2 h of incubation at 37 °C (% of control)
**Control** (non-treated cells)	1×10^{8} CFU (100)
**PMB**	1×10^{6} CFU (1)
**Onc**	1×10^{4} CFU (0.01)
**In65**	1×10^{4} CFU (0.01)
**EB5**	1×10^{5} CFU (0.1)
**EB9**	1×10^{4} CFU (0.01)

9. HPLC and MS data

VDKPPYLPRPRPPRRIYNR (Oncocin) was obtained as a foamy white solid after preparative RP-HPLC (111.0 mg, 34.0 %). **Analytical RP-HPLC**: t_R = 2.36 min (A/D 100:0 to 0:100 in 7.0 min, λ = 214 nm). **MS** (ESI$^+$): $C_{109}H_{177}N_{37}O_{24}$ calc./found 2389.3767/2389.3857 Da $[M+H]^+$.

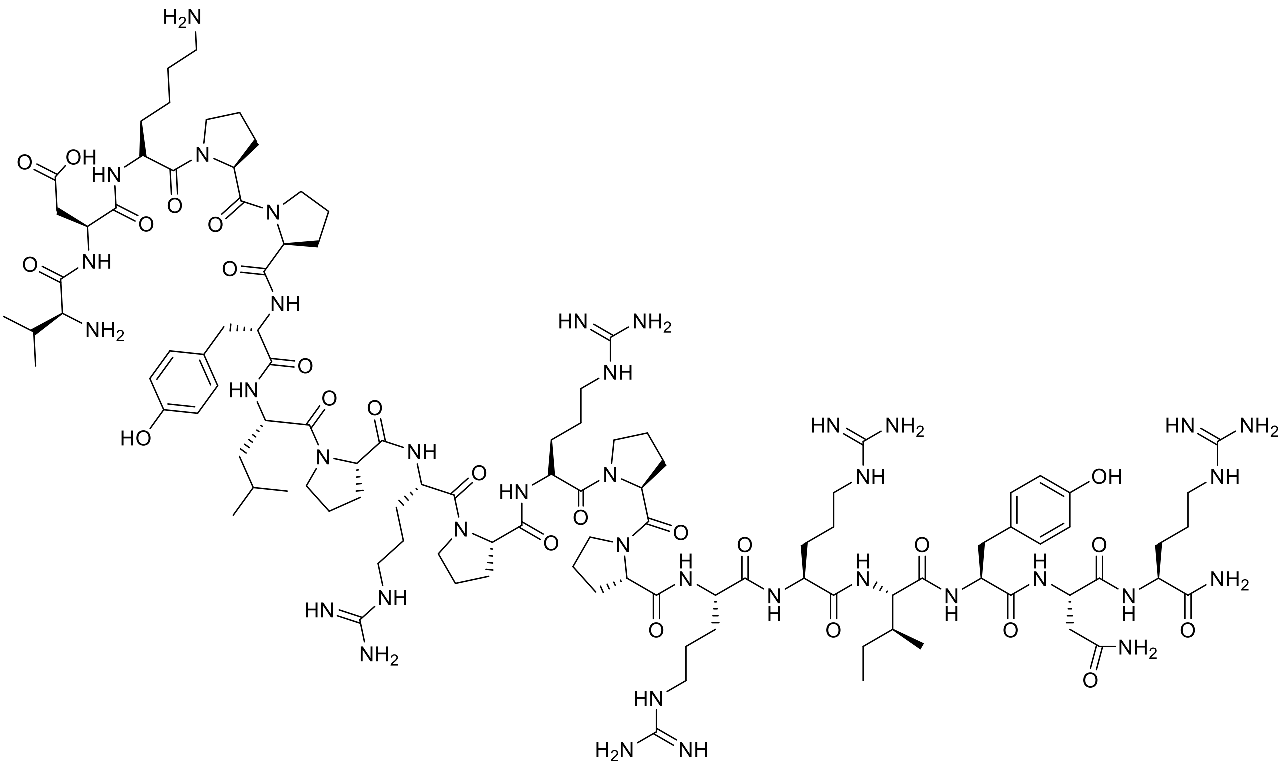

Chemical Formula: $C_{109}H_{177}N_{37}O_{24}$
Exact Mass: 2388.3767
Molecular Weight: 2389.8500

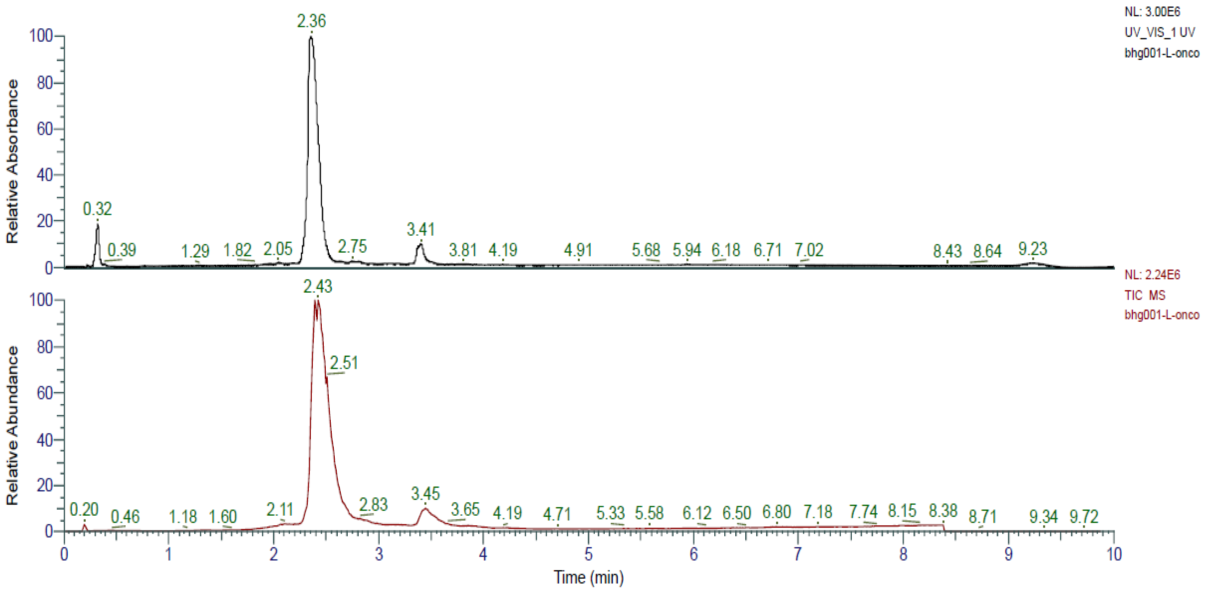

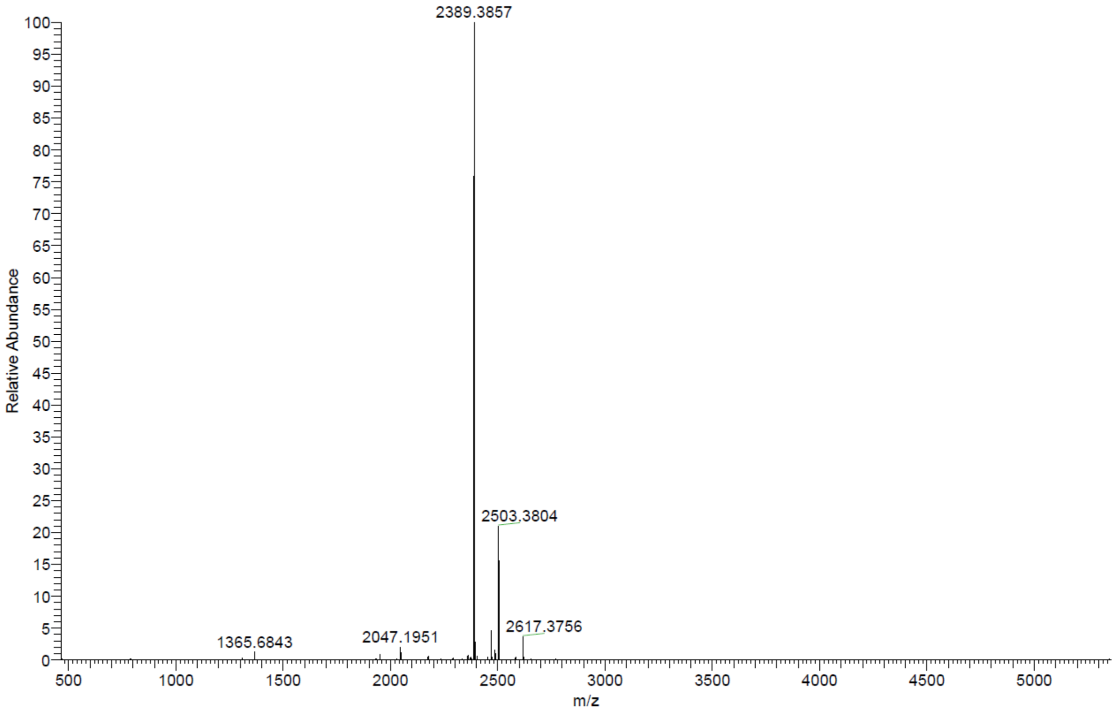
2389.3857
2503.3804
2617.3756
2047.1951
1365.6843
Relative Abundance
m/z

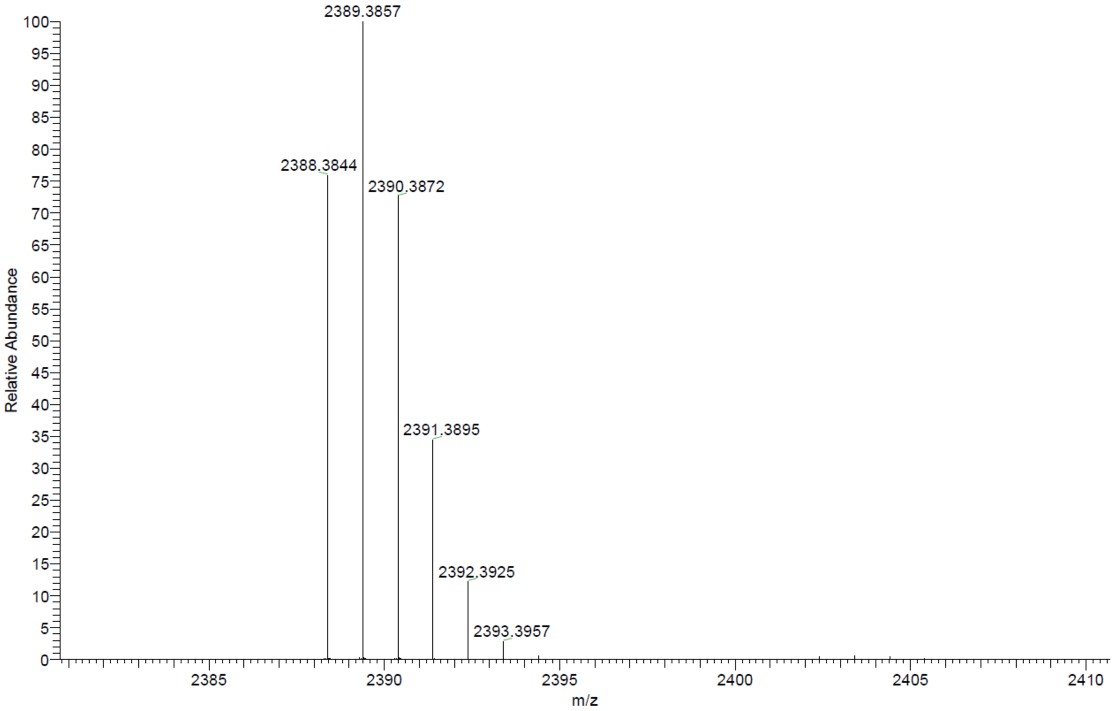
2389.3857
2388.3844
2390.3872
2391.3895
2392.3925
2393.3957
Relative Abundance
m/z

KKLLKLLKLLL (ln65) was obtained as a foamy white solid after preparative RP-HPLC (84.0 mg, 58.0 %). **Analytical RP-HPLC**: t_R = 3.63 min (A/D 100:0 to 0:100 in 7.0 min, λ = 214 nm). **MS** (ESI$^+$): $C_{66}H_{128}N_{16}O_{11}$ calc./obs. 1321.9948/1322.0021 Da $[M+H]^+$.

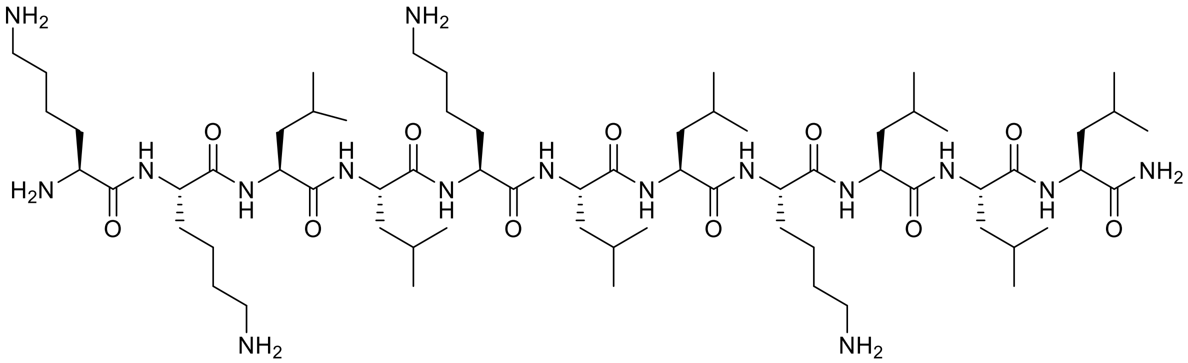

Chemical Formula: $C_{66}H_{128}N_{16}O_{11}$
Exact Mass: 1320.9948
Molecular Weight: 1321.8510

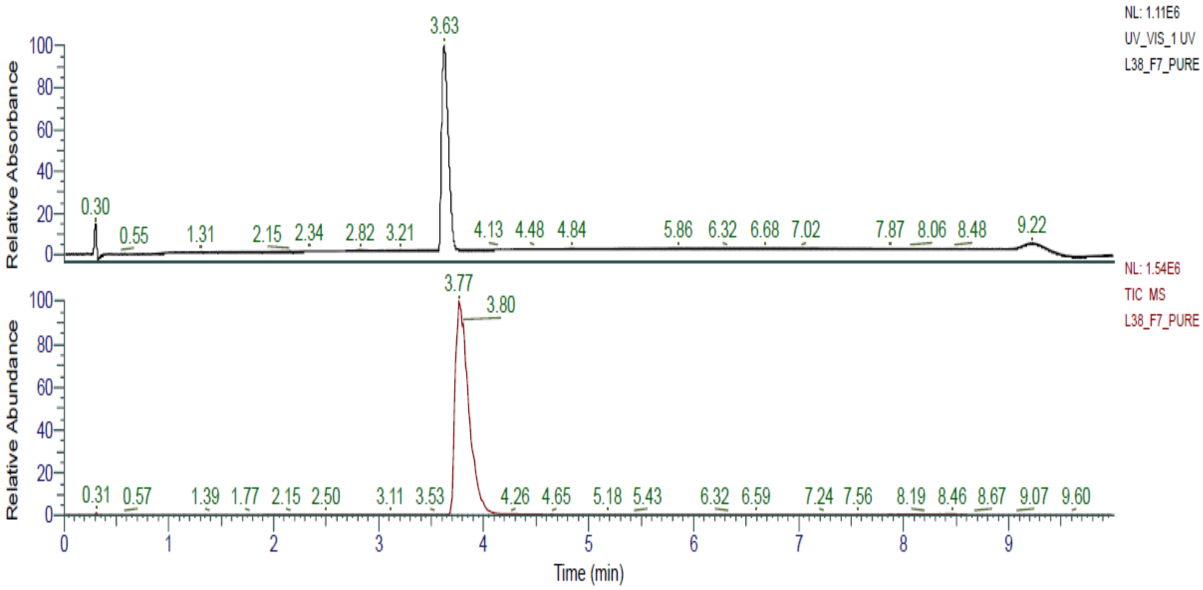

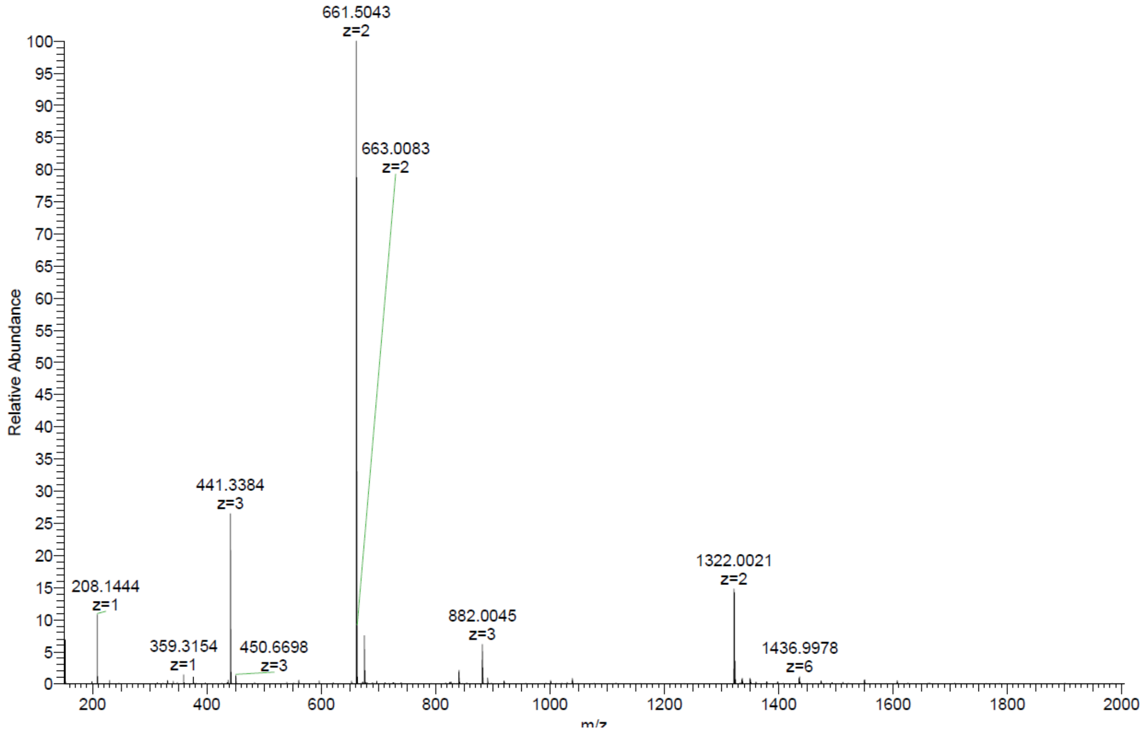

661.5043
z=2
663.0083
z=2
441.3384
z=3
208.1444
z=1
359.3154
z=1
450.6698
z=3
882.0045
z=3
1322.0021
z=2
1436.9978
z=6
Relative Abundance

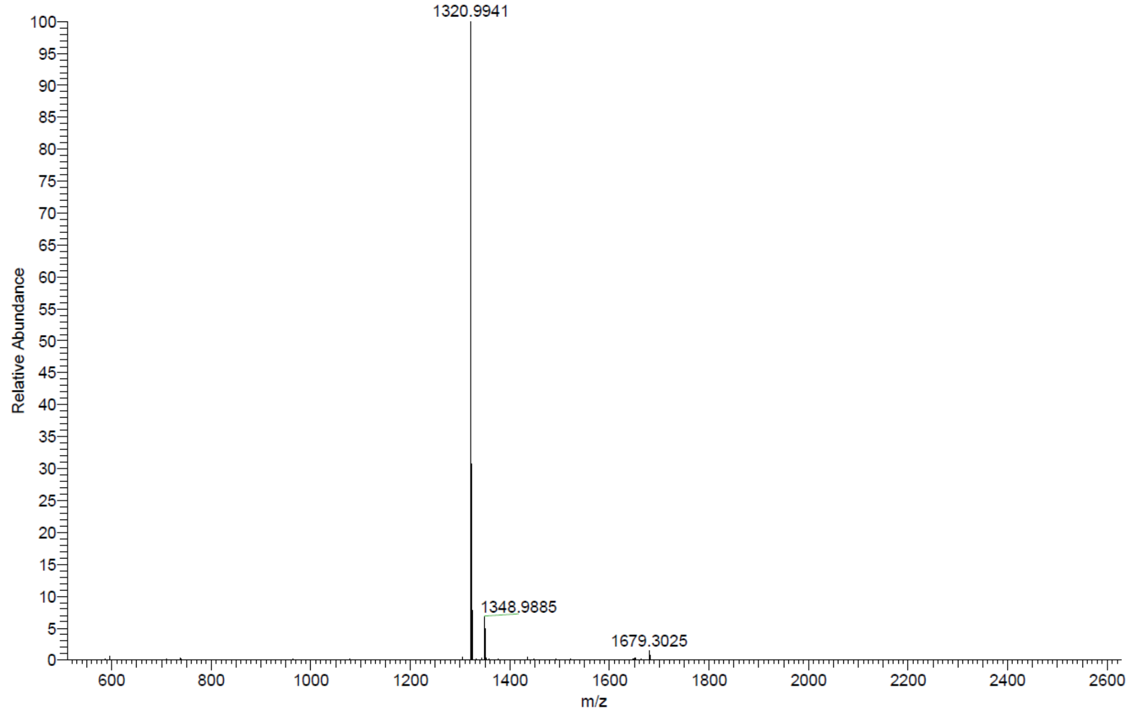

1320.9941
1348.9885
1679.3025
Relative Abundance
m/z

kkLLkLLkLLL (ln69) was obtained as a foamy white solid after preparative RP-HPLC (102.6 mg, 70.9 %). **Analytical RP-HPLC**: t_R = 3.23 min (A/D 100:0 to 0:100 in 7.0 min, λ = 214 nm). **MS** (ESI$^+$): $C_{66}H_{128}N_{16}O_{11}$ calc./obs. 1321.9948/1322.0027 Da $[M+H]^+$.

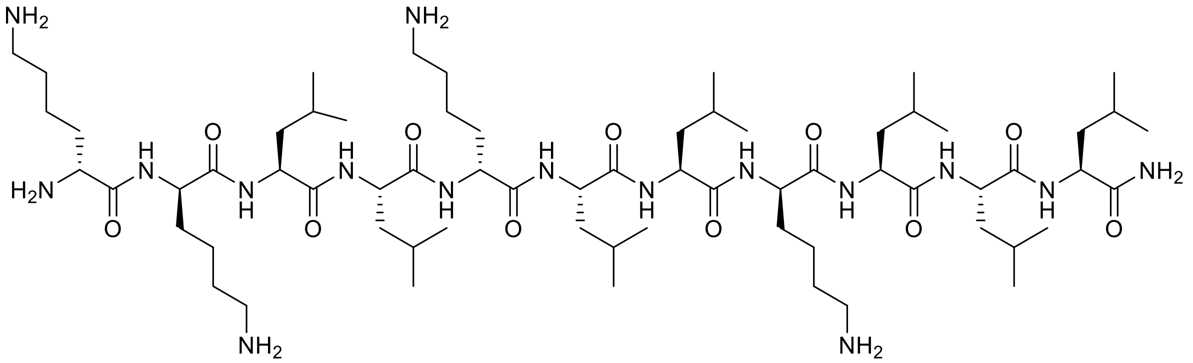

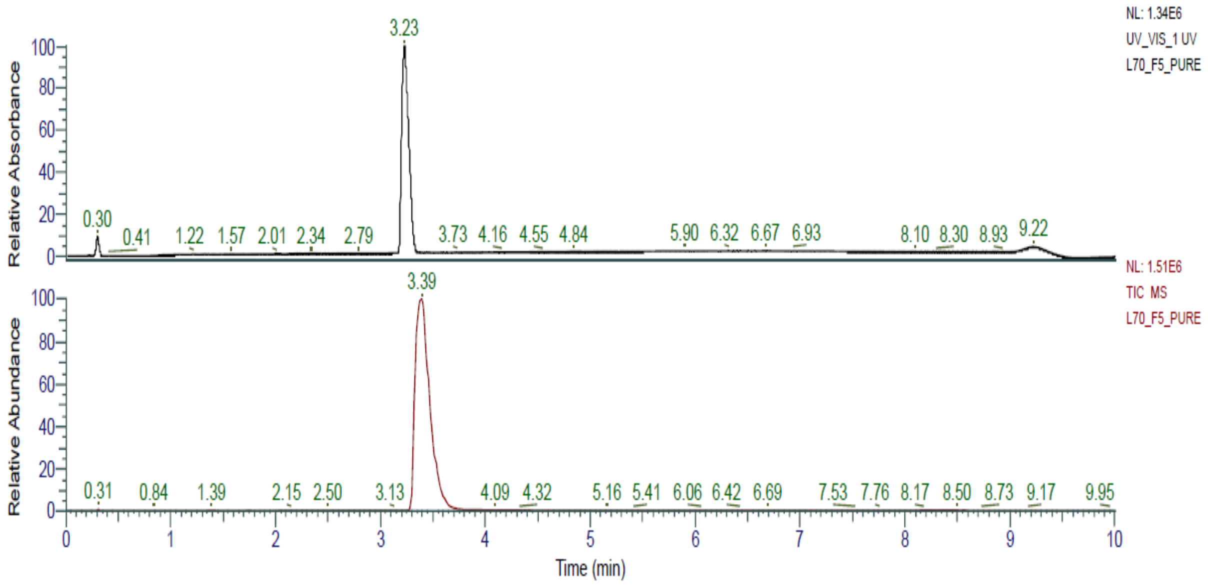

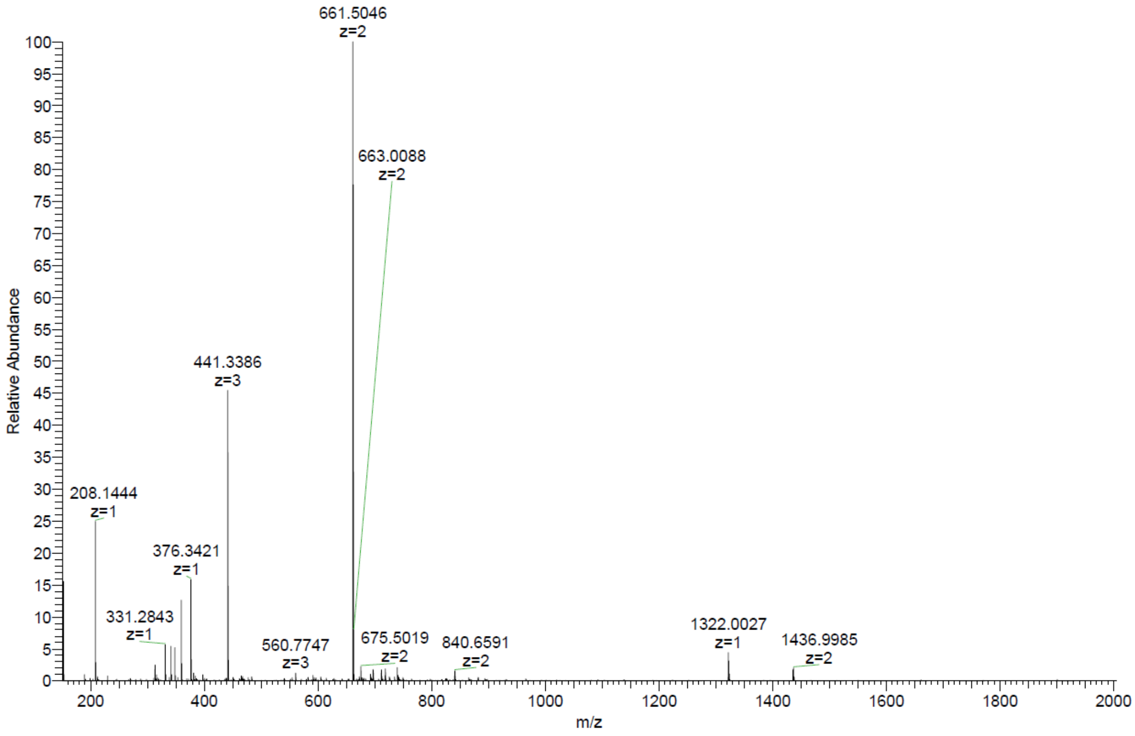
661.5046
z=2
663.0088
z=2
441.3386
z=3
208.1444
z=1
376.3421
z=1
331.2843
z=1
560.7747
z=3
675.5019
z=2
840.6591
z=2
1322.0027
z=1
1436.9985
z=2
Relative Abundance
m/z

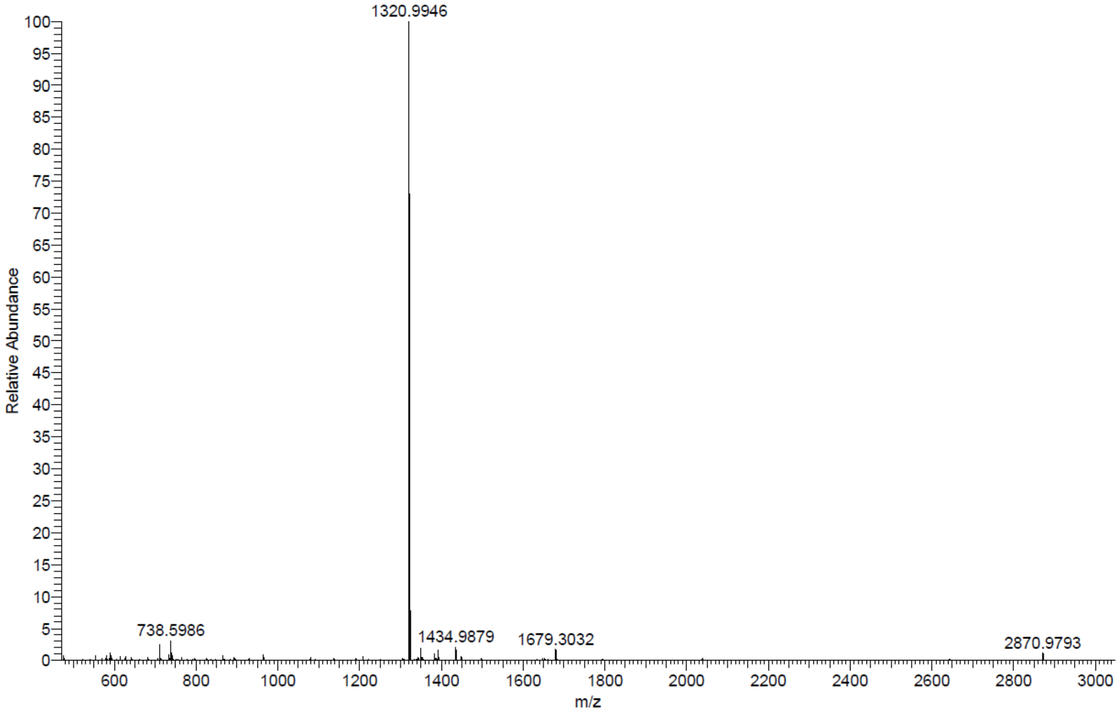
1320.9946
738.5986
1434.9879
1679.3032
2870.9793
Relative Abundance
m/z

***k*KLLKLLKLL*l* (EB1)** was obtained as a foamy white powder after preparative RP-HPLC (77.4 mg, 37.5 %). **Analytical RP-HPLC**: t_R = 3.50 min (A/D 100:0 to 0:100 in 7.0 min, λ = 214 nm). **MS** (ESI^+): $C_{66}H_{128}N_{16}O_{11}$ calc./obs. 1321.9948/1321.9937 Da ([M]).

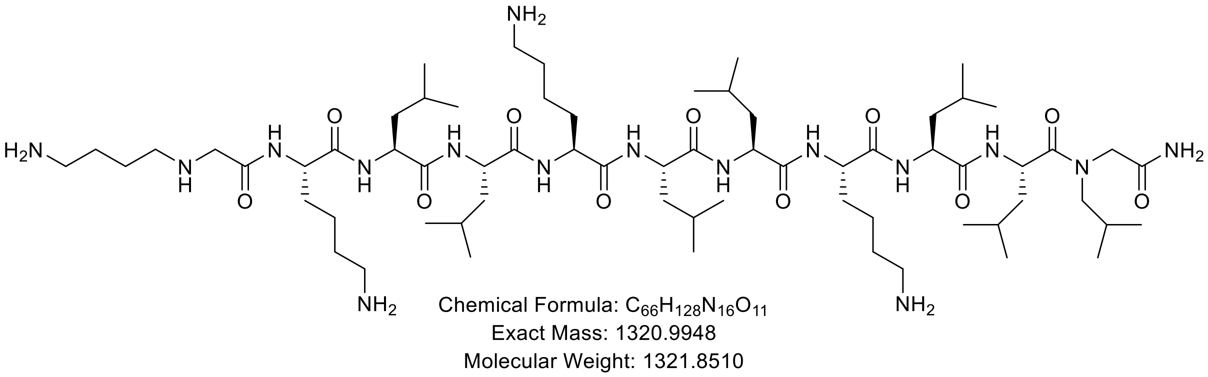

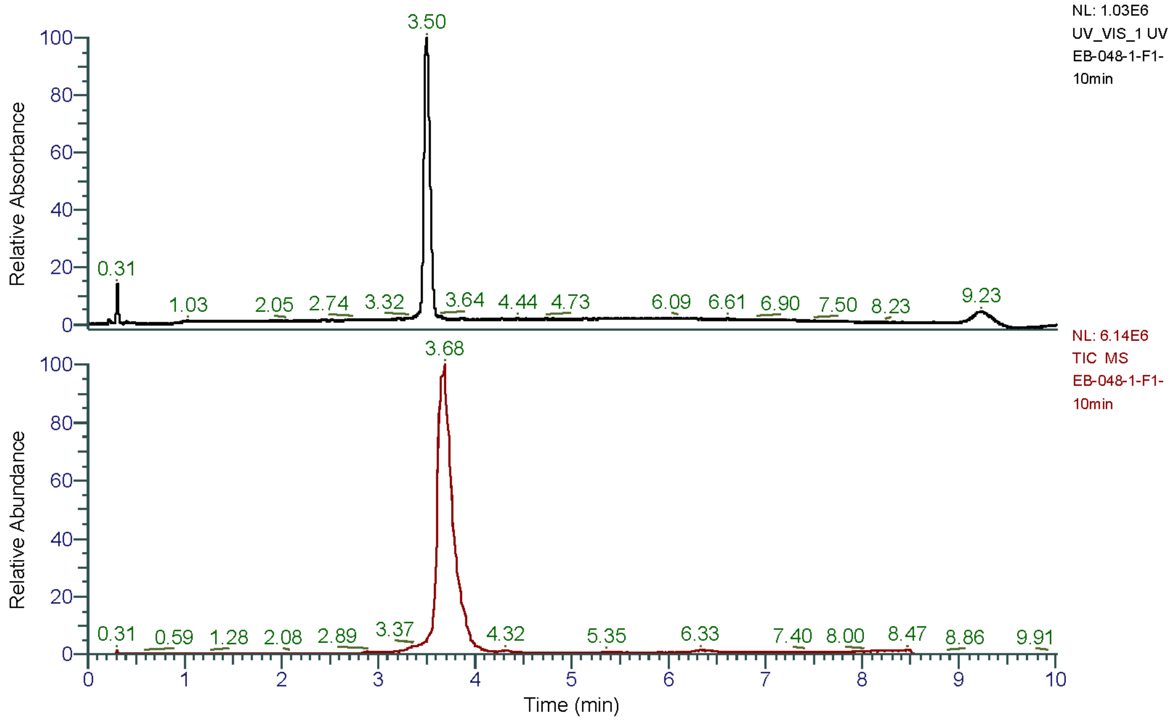

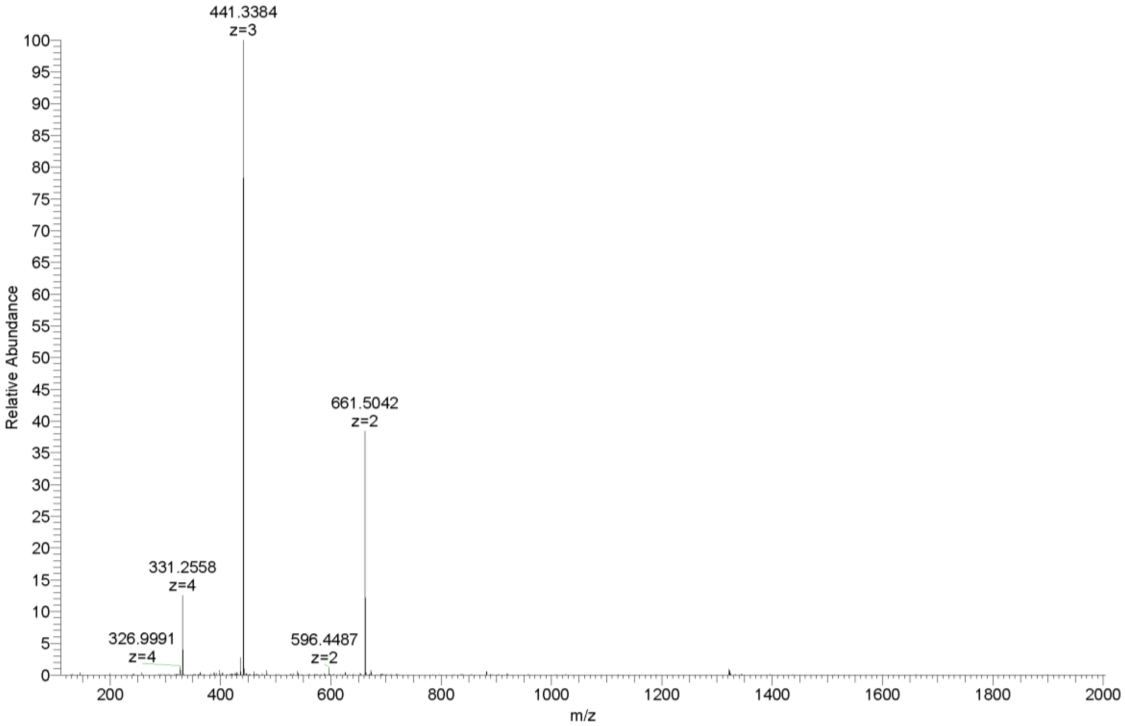
441.3384
z=3
661.5042
z=2
331.2558
z=4
326.9991
z=4
596.4487
z=2
Relative Abundance
m/z

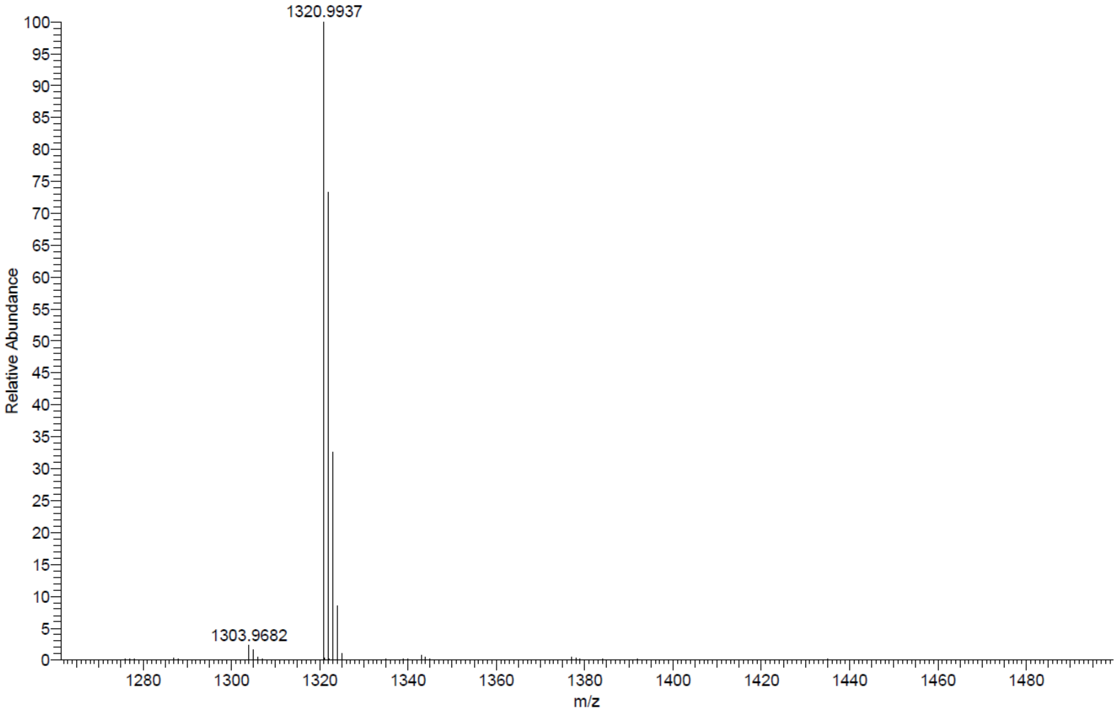
1320.9937
1303.9682
Relative Abundance
m/z

***k*KLLKLL*k*LL*l* (EB2)** was obtained as a foamy white powder after preparative RP-HPLC (69.0 mg, 33.5 %). **Analytical RP-HPLC**: t_R = 3.40 min (A/D 100:0 to 0:100 in 7.0 min, λ = 214 nm). **MS** (ESI^+): $C_{66}H_{128}N_{16}O_{11}$ calc./obs. 1321.9948/1321.9995 Da $[M+H]^+$.

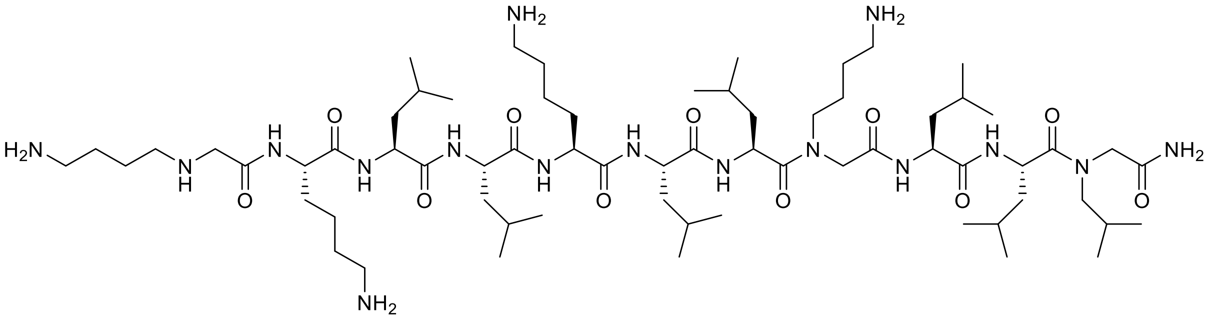

Chemical Formula: $C_{66}H_{128}N_{16}O_{11}$
Exact Mass: 1320.9948
Molecular Weight: 1321.8510

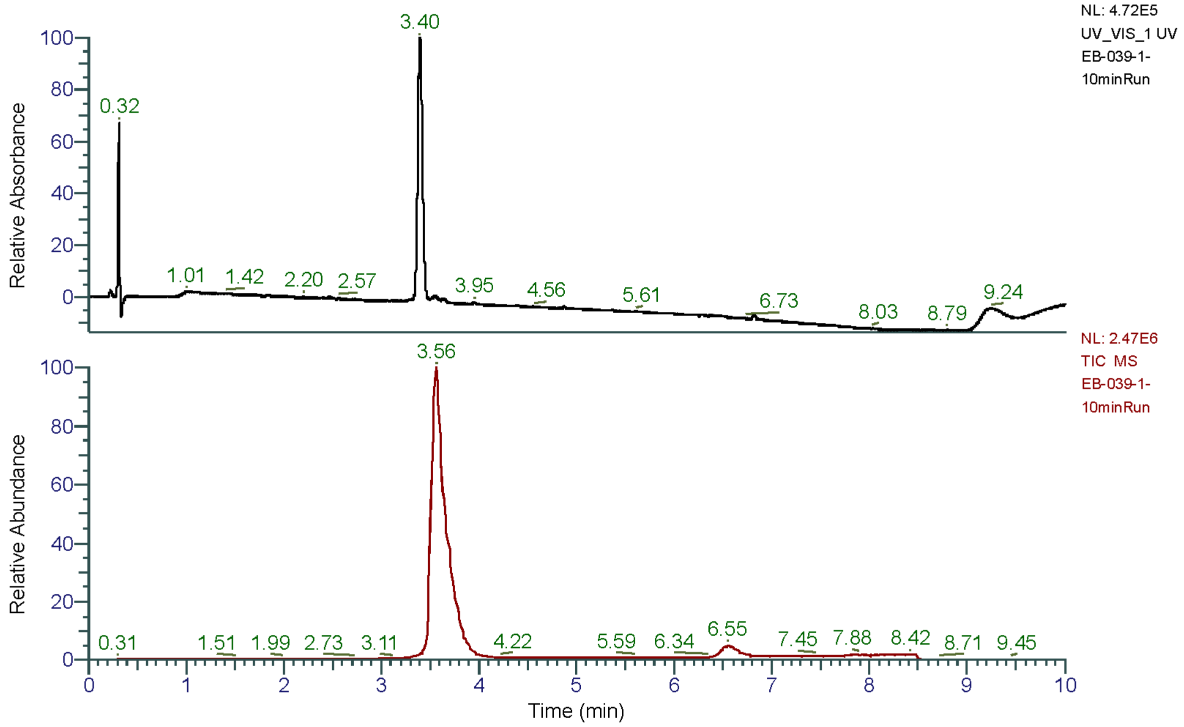

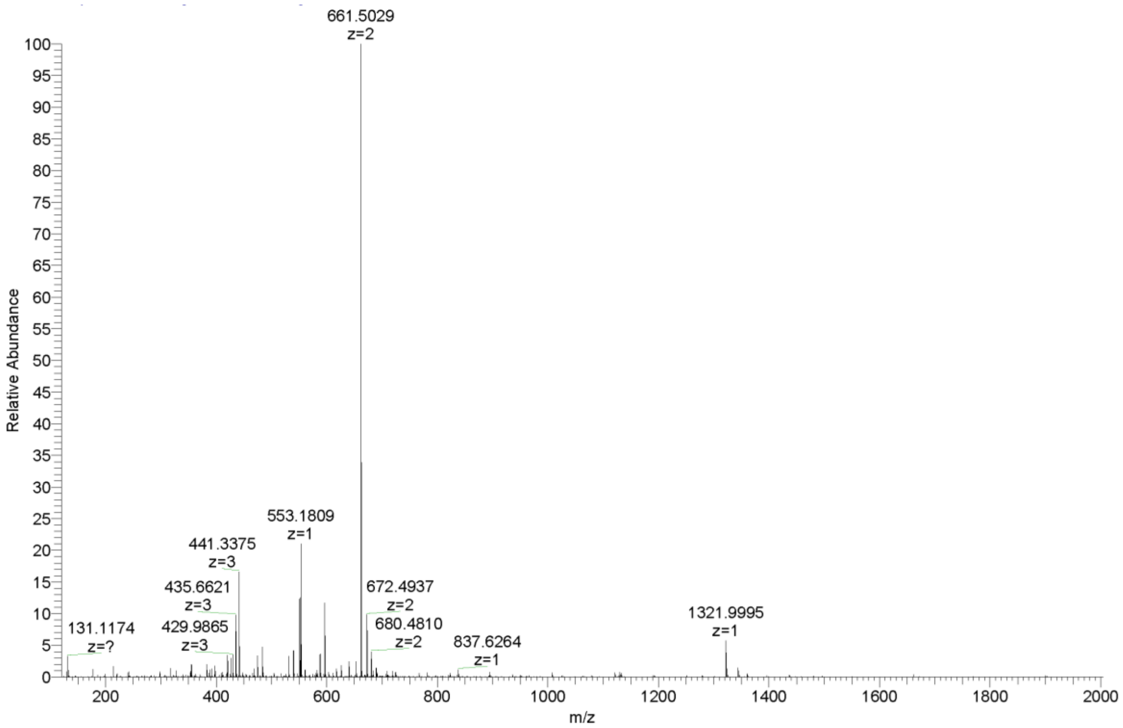
661.5029
z=2
553.1809
z=1
441.3375
z=3
435.6621
z=3
429.9865
z=3
131.1174
z=?
672.4937
z=2
680.4810
z=2
837.6264
z=1
1321.9995
z=1
Relative Abundance
m/z

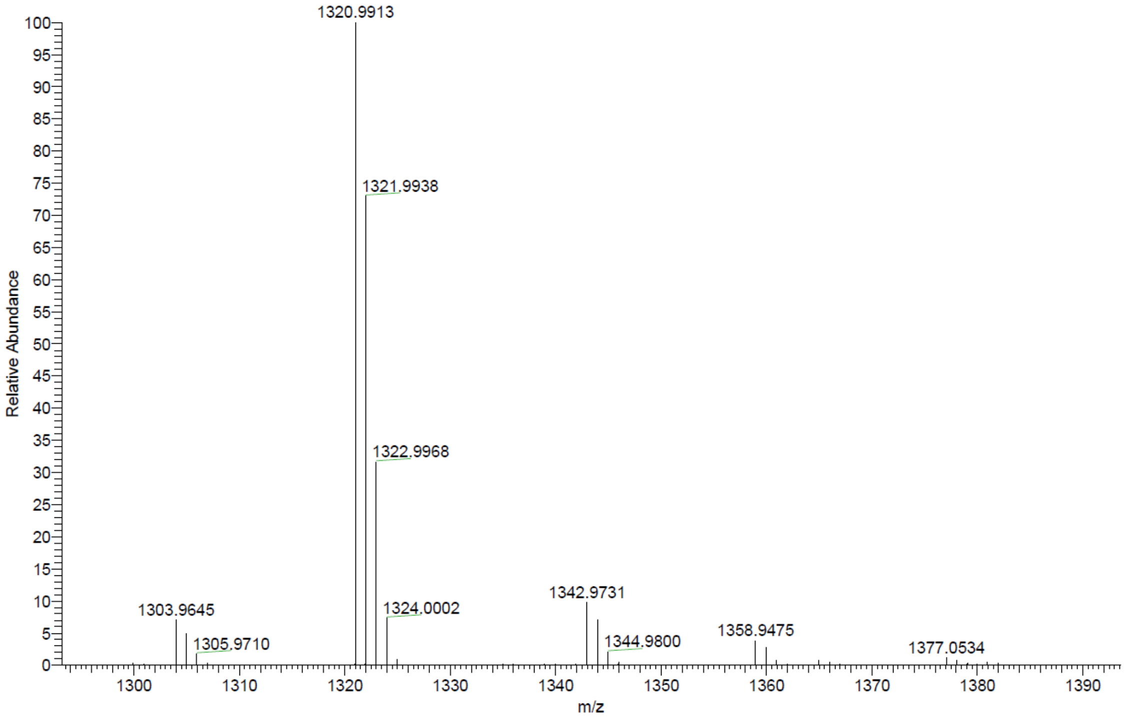
1320.9913
1321.9938
1322.9968
1324.0002
1303.9645
1305.9710
1342.9731
1344.9800
1358.9475
1377.0534
Relative Abundance
m/z

KKLLKLLK*lll* (EB3) was obtained as a foamy white powder after preparative RP-HPLC (54.1 mg, 26.2 %). **Analytical RP-HPLC**: t_R = 3.23 min (A/D 100:0 to 0:100 in 7.0 min, λ = 214 nm). **MS** (ESI$^+$): $C_{66}H_{128}N_{16}O_{11}$ calc./obs. 1321.9948/1322.0028 Da $[M+H]^+$.

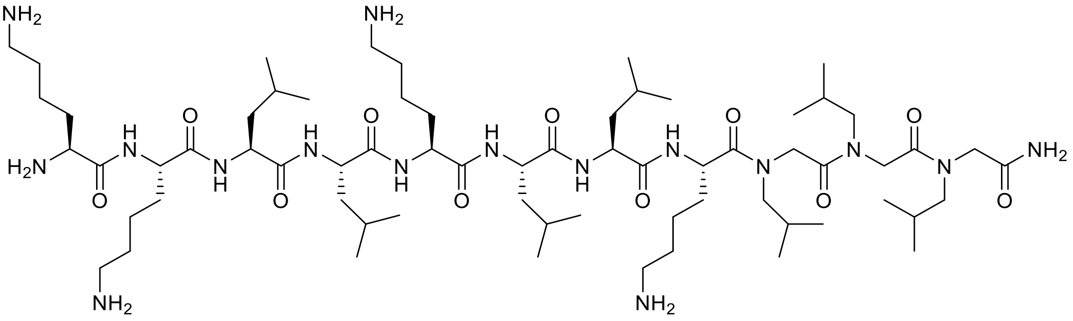

Chemical Formula: $C_{66}H_{128}N_{16}O_{11}$
Exact Mass: 1320.9948
Molecular Weight: 1321.8510

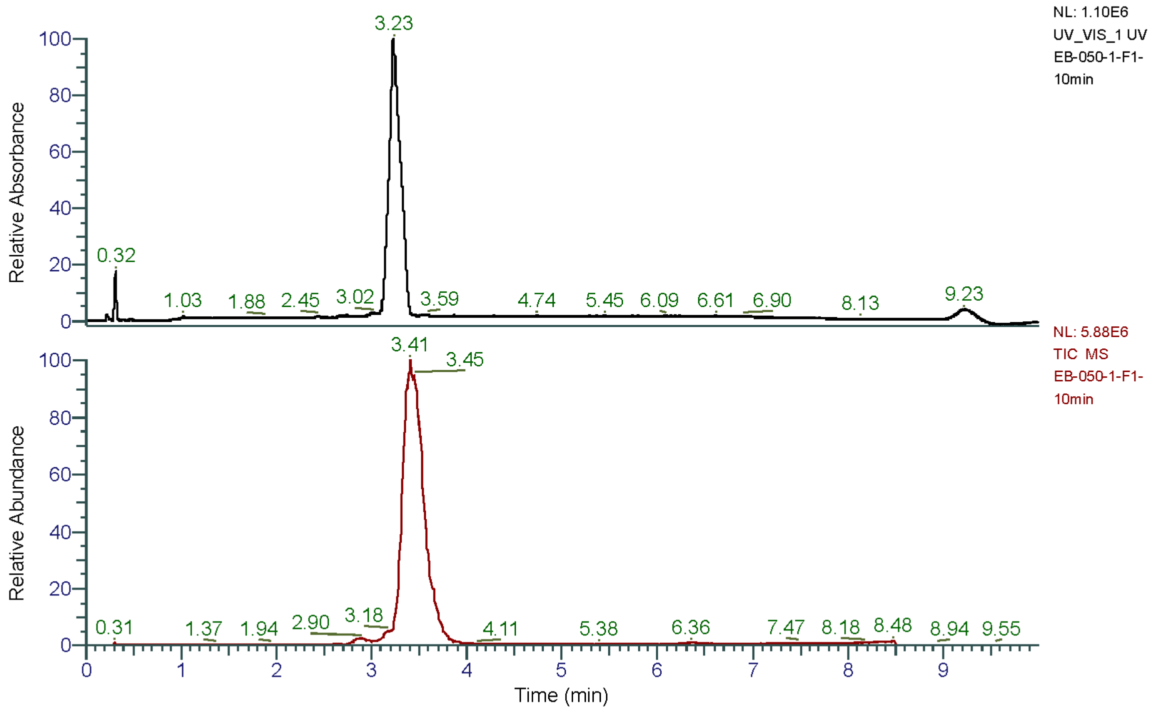

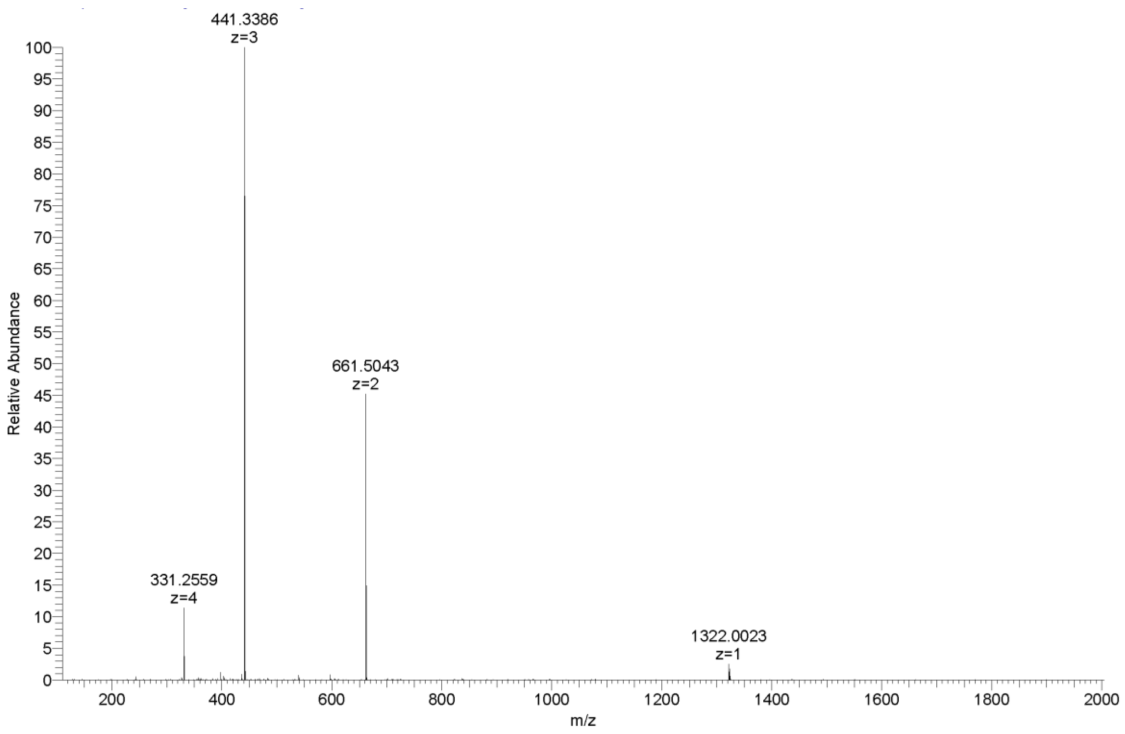
441.3386
z=3
661.5043
z=2
331.2559
z=4
1322.0023
z=1
Relative Abundance
m/z

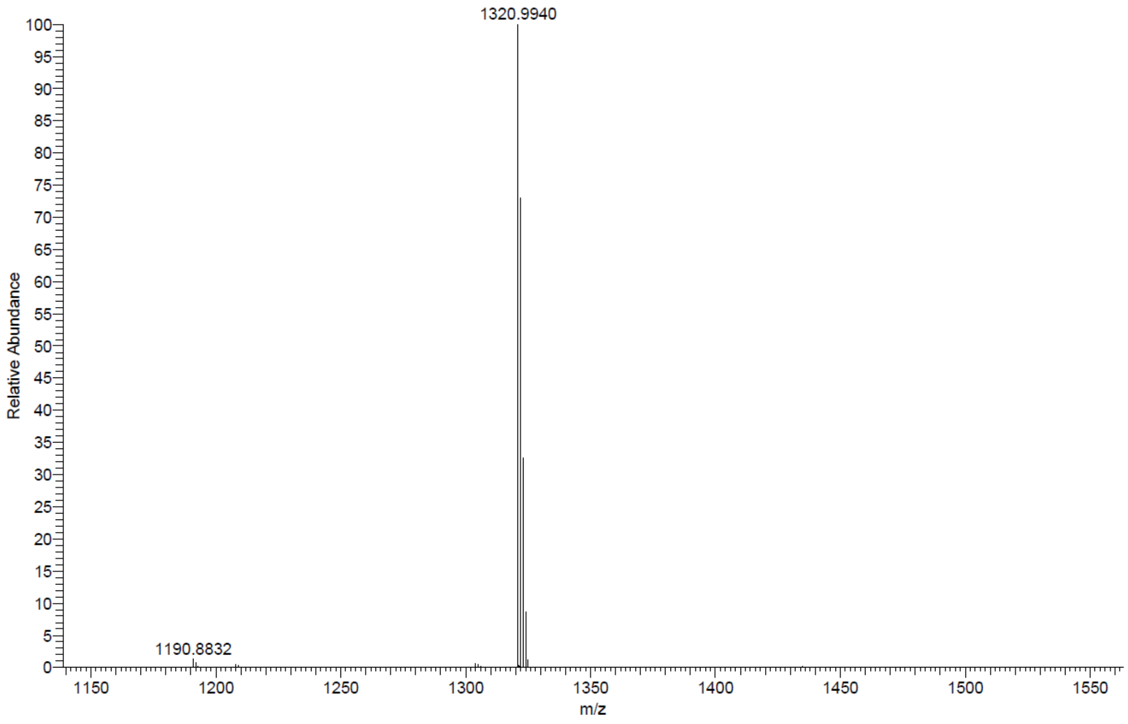
1320.9940
1190.8832
Relative Abundance
m/z

KKLLK*llklll* (EB4) was obtained as a colourless sticky solid after preparative RP-HPLC (38.0 mg, 18.4 %). **Analytical RP-HPLC**: t_R = 3.30 min (A/D 100:0 to 0:100 in 7.0 min, λ = 214 nm). **MS** (ESI$^+$): $C_{66}H_{128}N_{16}O_{11}$ calc./obs. 1321.9948/1322.0014 Da [M+H]$^+$.

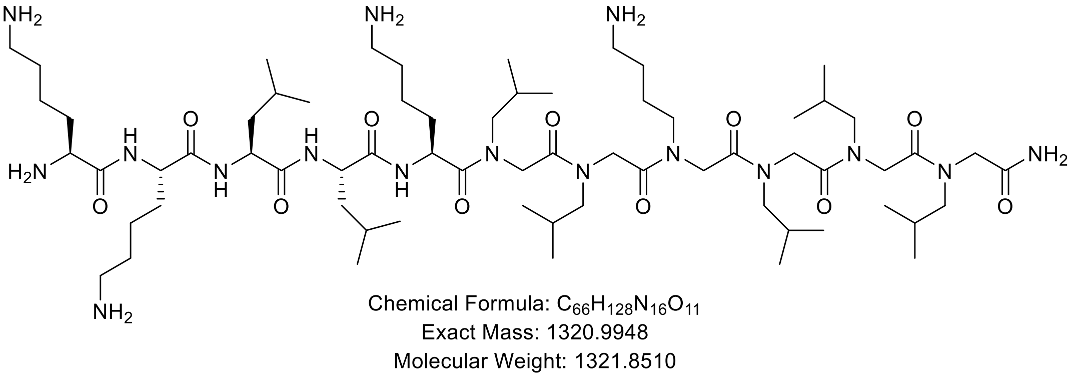

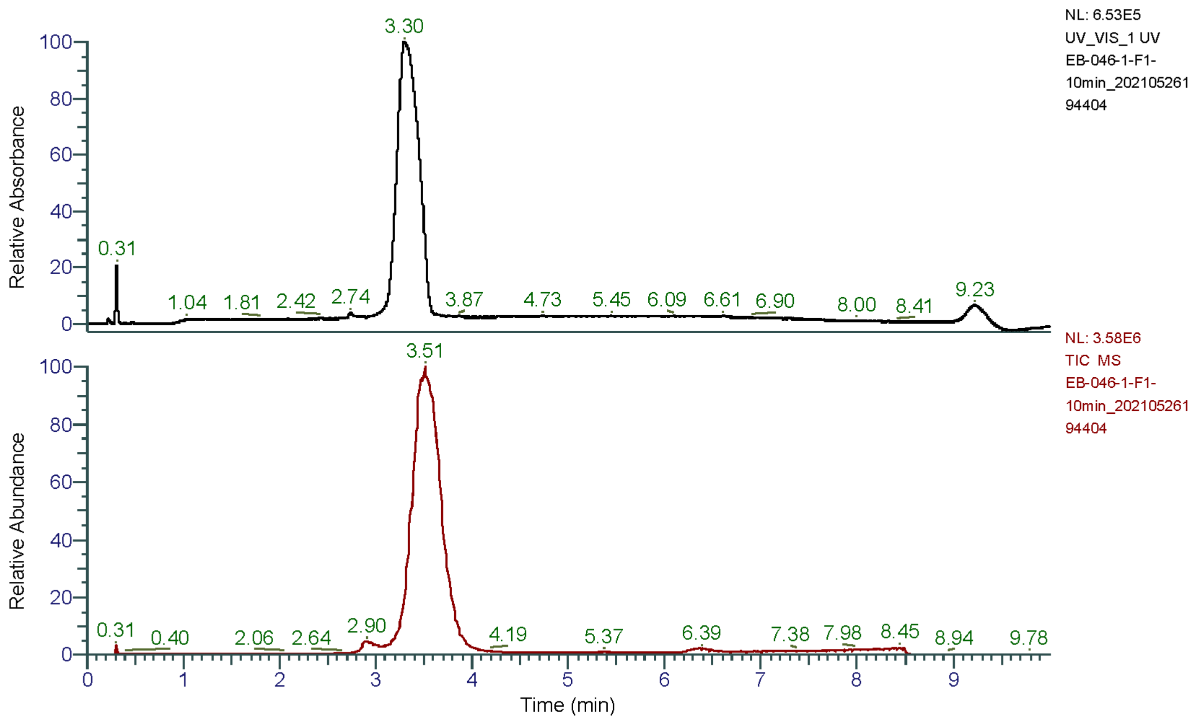

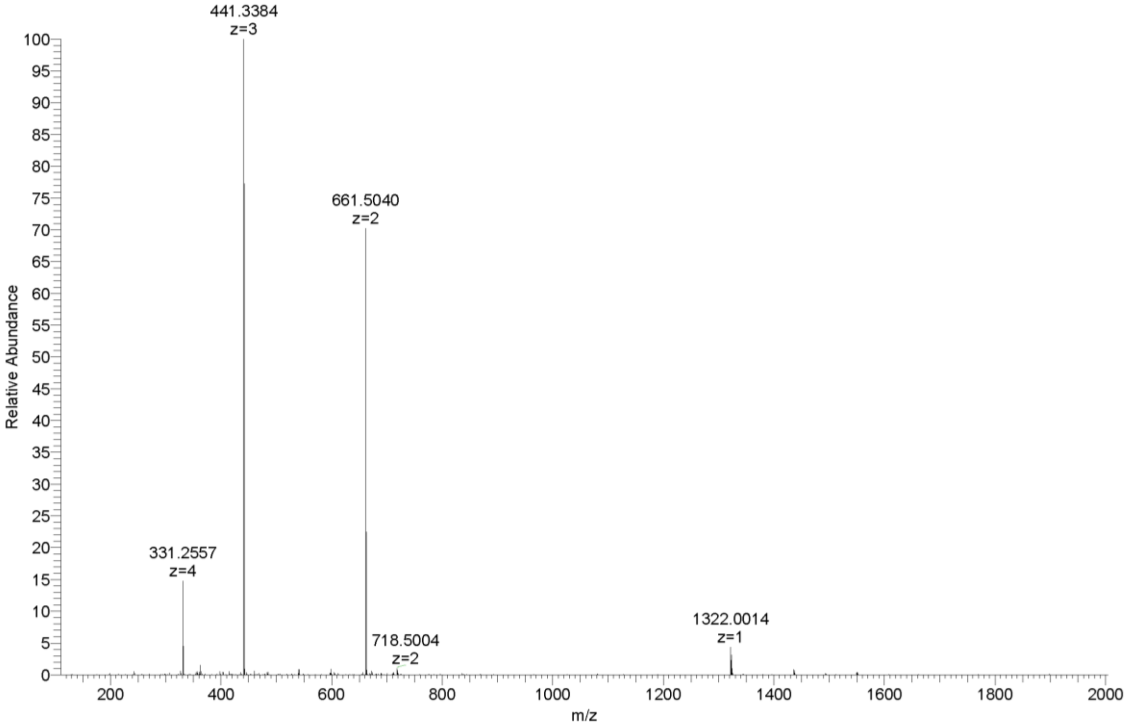
441.3384
z=3
661.5040
z=2
331.2557
z=4
718.5004
z=2
1322.0014
z=1
Relative Abundance
m/z

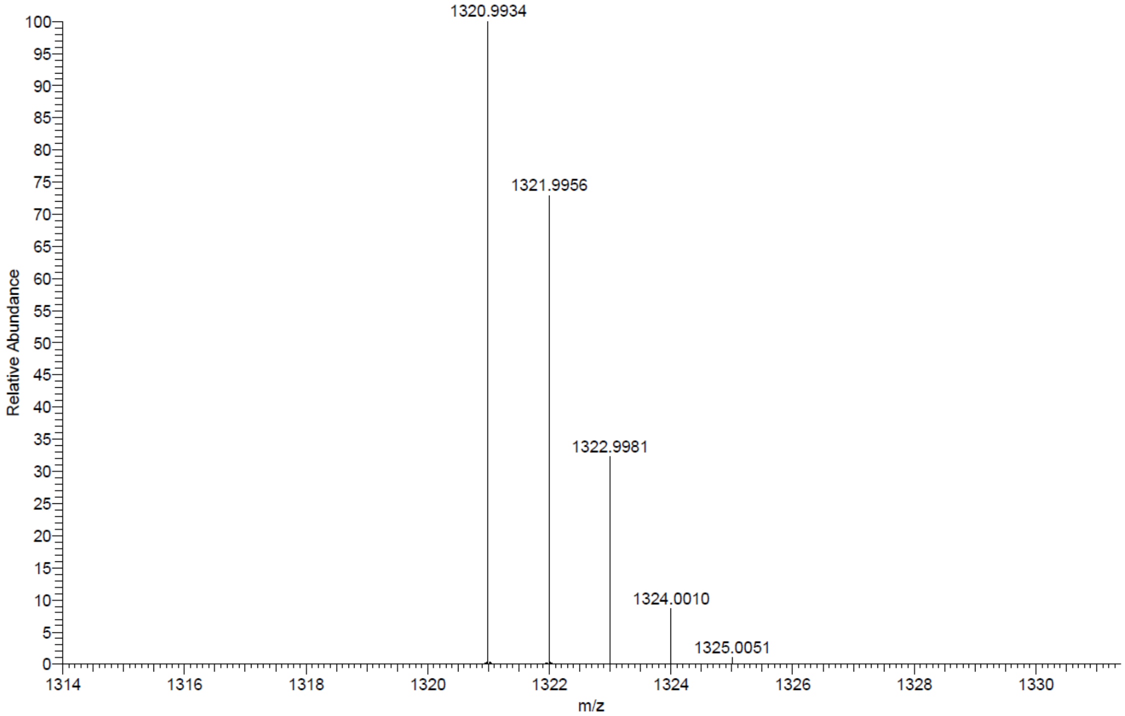
1320.9934
1321.9956
1322.9981
1324.0010
1325.0051
Relative Abundance
m/z

***kkllk*LLKLLL (EB5)** was obtained as a colourless sticky solid after preparative RP-HPLC (61.0 mg, 29.6 %). **Analytical RP-HPLC**: t_R = 3.50 min (A/D 100:0 to 0:100 in 7.0 min, λ = 214 nm). **MS** (ESI$^+$): $C_{66}H_{128}N_{16}O_{11}$ calc./obs. 1321.9948/1322.0019 Da $[M+H]^+$.

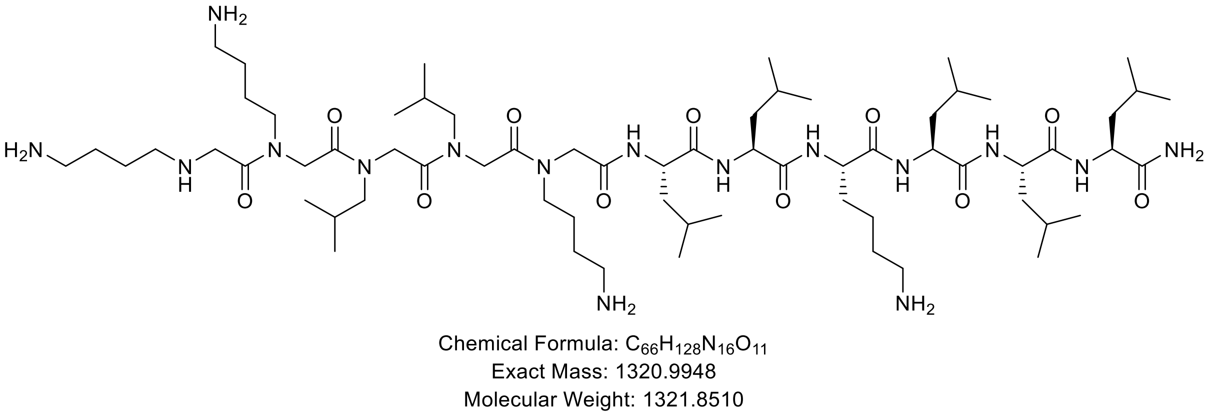

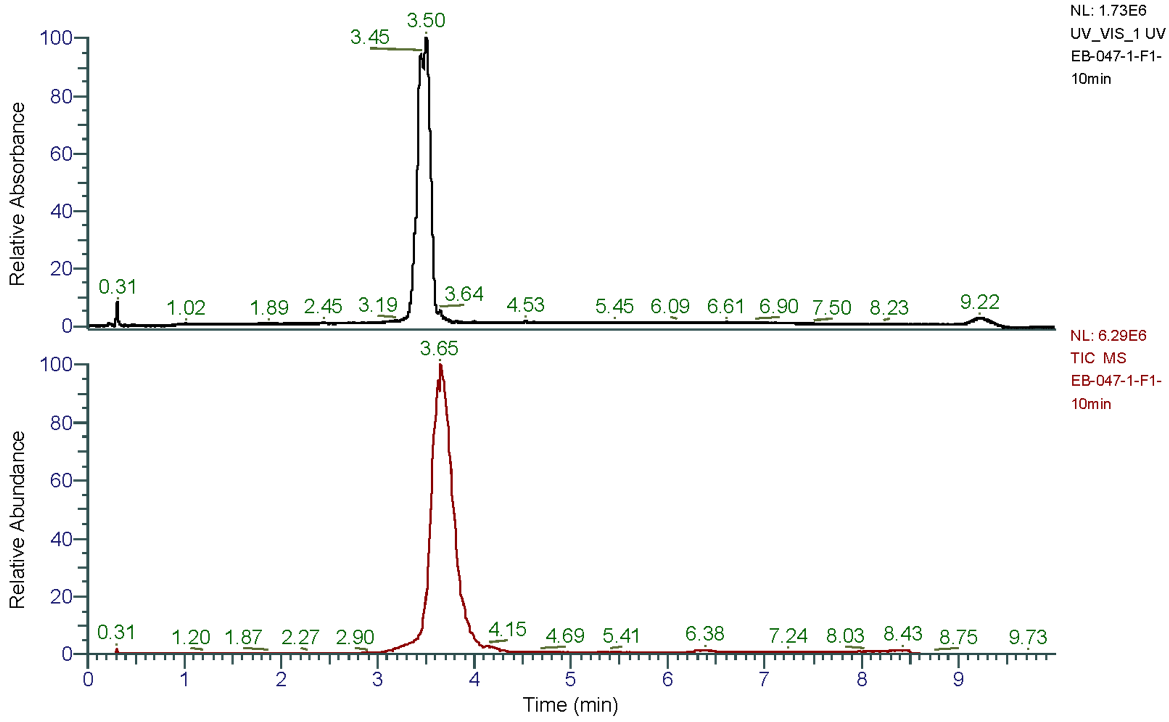

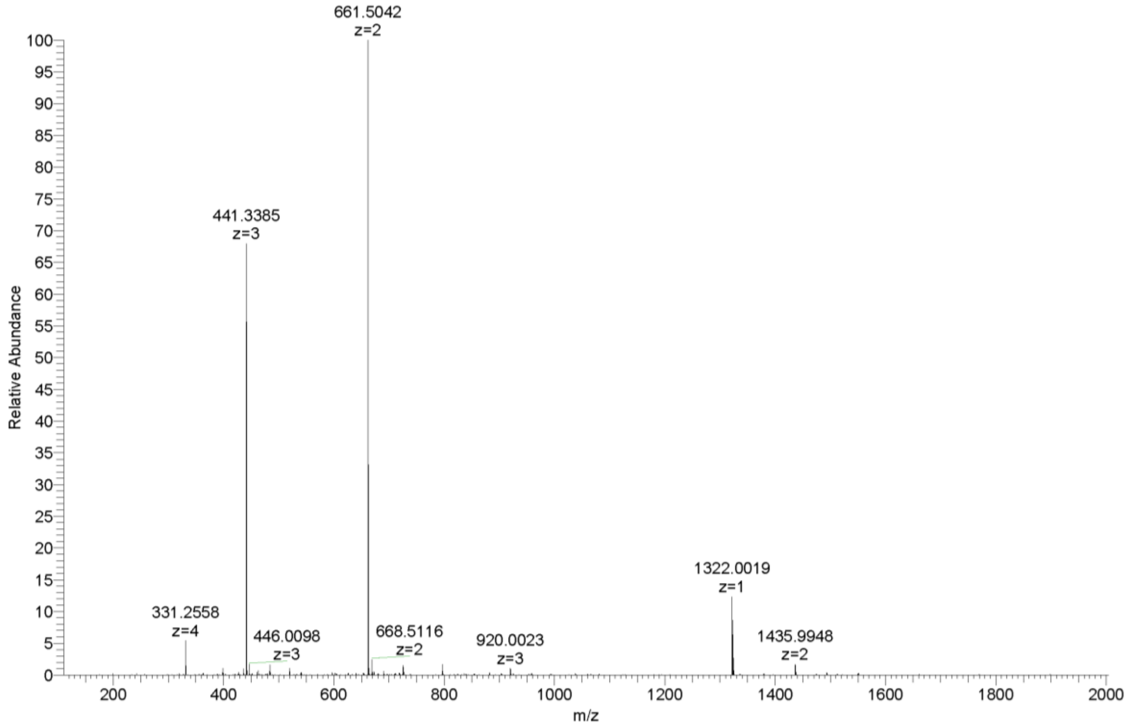
661.5042
z=2
441.3385
z=3
331.2558
z=4
446.0098
z=3
668.5116
z=2
920.0023
z=3
1322.0019
z=1
1435.9948
z=2
Relative Abundance
m/z

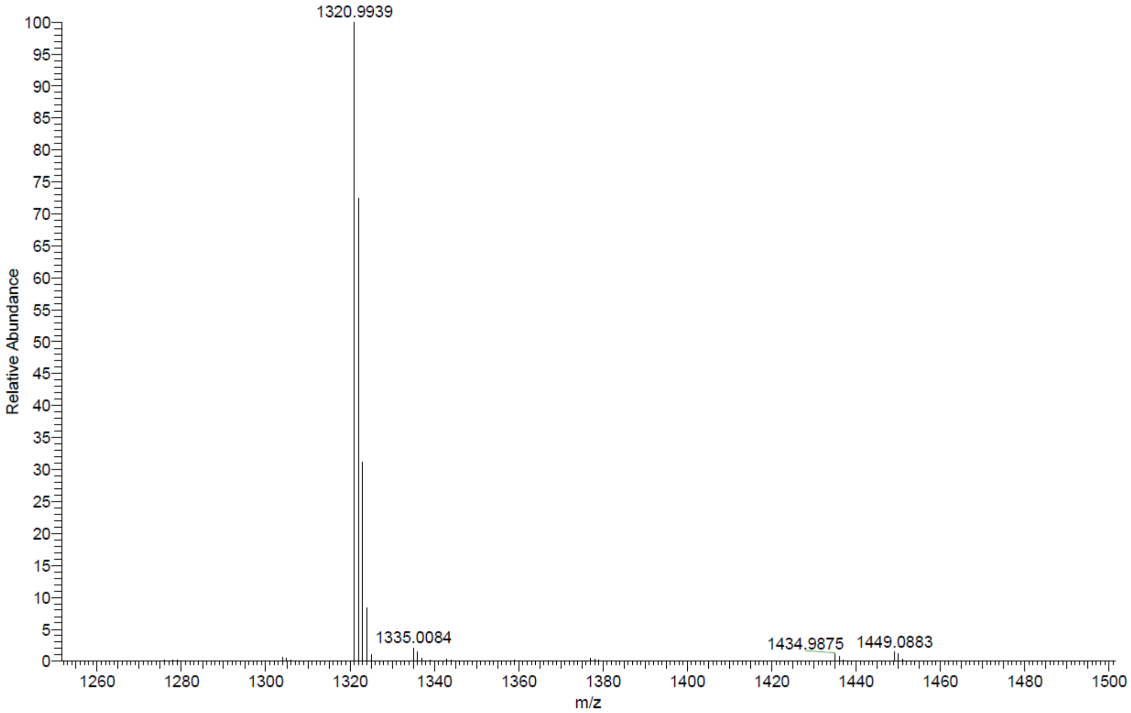
1320.9939
1335.0084
1434.9875
1449.0883
Relative Abundance
m/z

***kk*LL*k*LLKLLL (EB6)** was obtained as a foamy white powder after preparative RP-HPLC (77.5 mg, 37.6 %). **Analytical RP-HPLC**: t_R = 3.58 min (A/D 100:0 to 0:100 in 7.0 min, λ = 214 nm). **MS** (ESI$^+$): $C_{66}H_{128}N_{16}O_{11}$ calc./obs. 1321.9948/1321.9995 Da [M+H]$^+$.

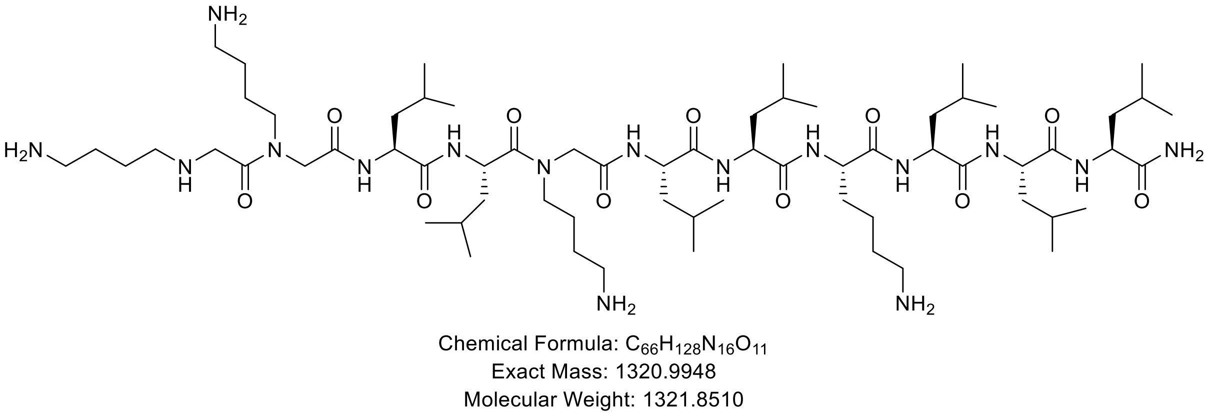

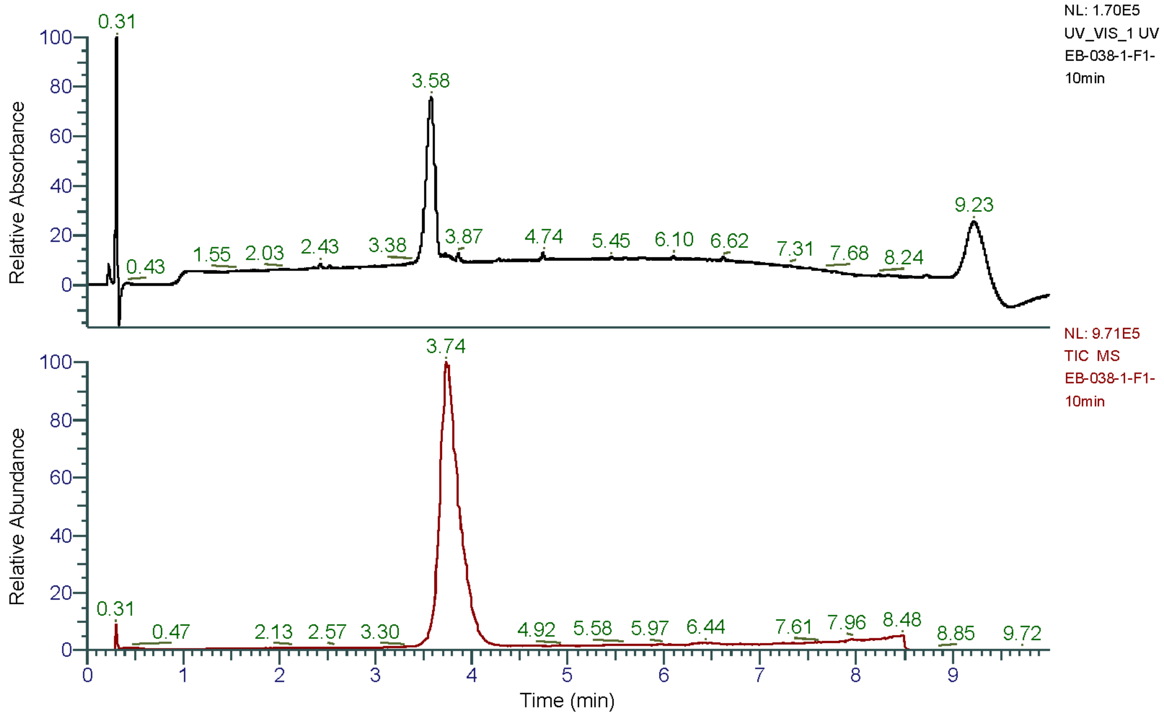

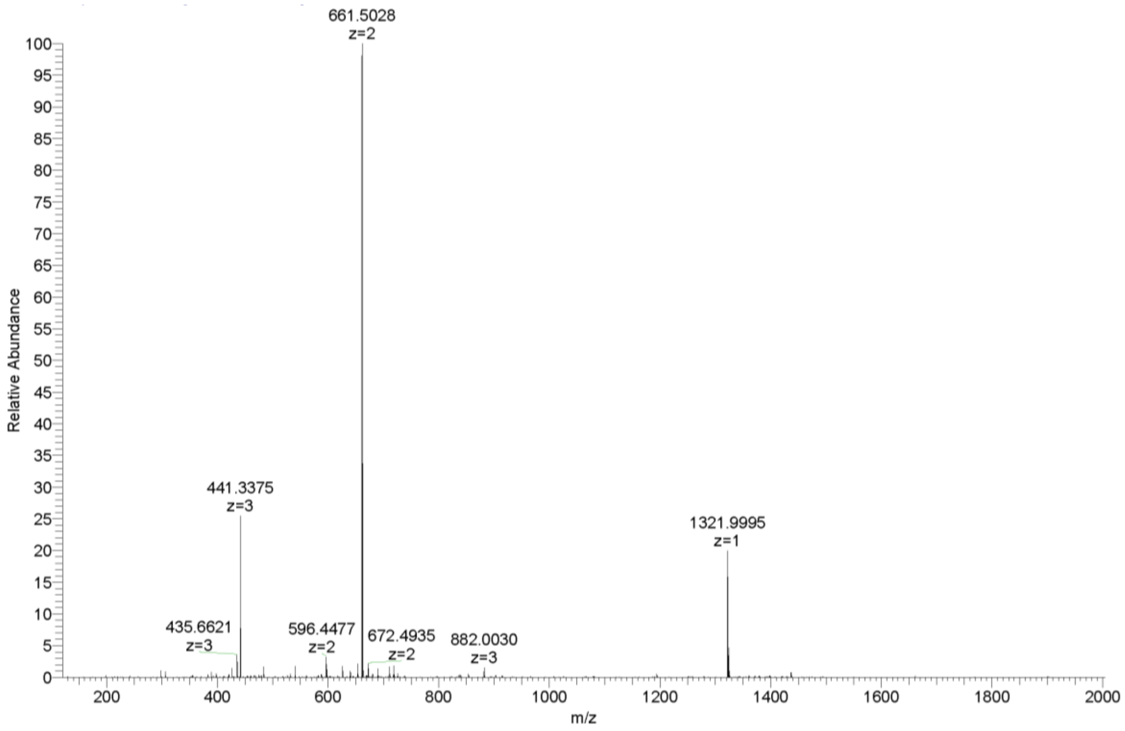

661.5028
z=2
441.3375
z=3
1321.9995
z=1
435.6621
z=3
596.4477
z=2
672.4935
z=2
882.0030
z=3
Relative Abundance
m/z

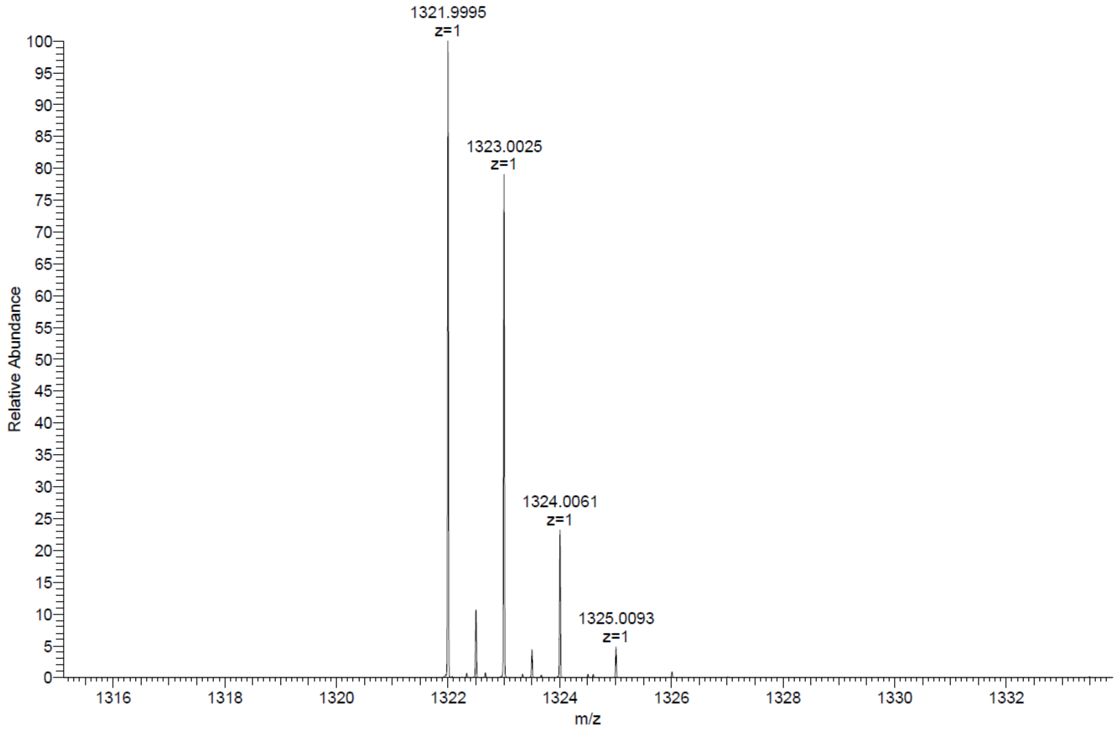

1321.9995
z=1
1323.0025
z=1
1324.0061
z=1
1325.0093
z=1
Relative Abundance
m/z

***kk*LL*k*LL*k*LLL (EB7)** was obtained as a foamy white powder after preparative RP-HPLC (18.4 mg, 8.9 %). **Analytical RP-HPLC**: t_R = 3.16 min (A/D 100:0 to 0:100 in 7.0 min, λ = 214 nm). **MS** (ESI$^+$): $C_{66}H_{128}N_{16}O_{11}$ calc./obs. 1321.9948/1322.0017 Da $[M+H]^+$.

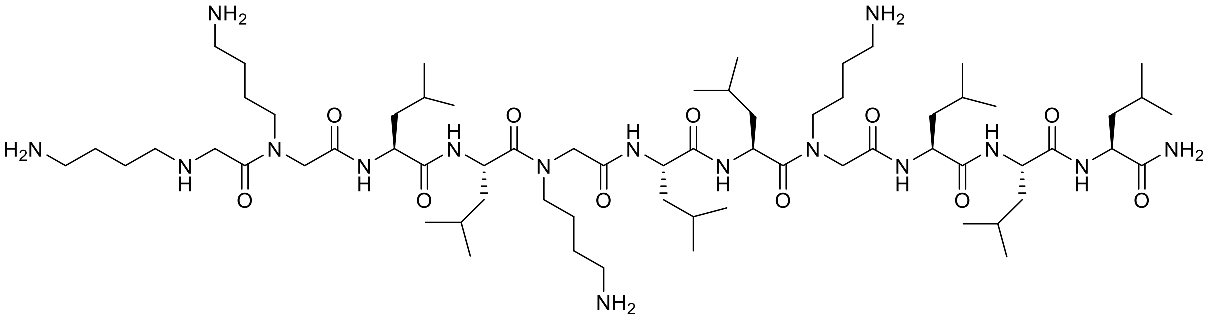

Chemical Formula: $C_{66}H_{128}N_{16}O_{11}$
Exact Mass: 1320.9948
Molecular Weight: 1321.8510

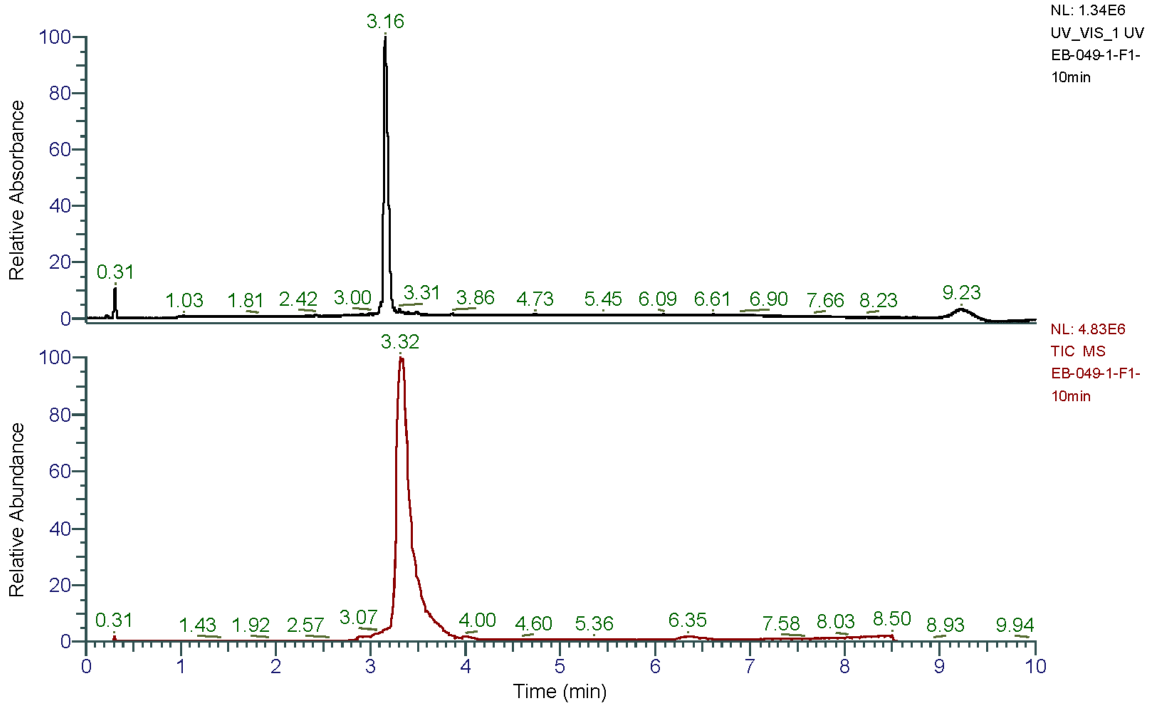

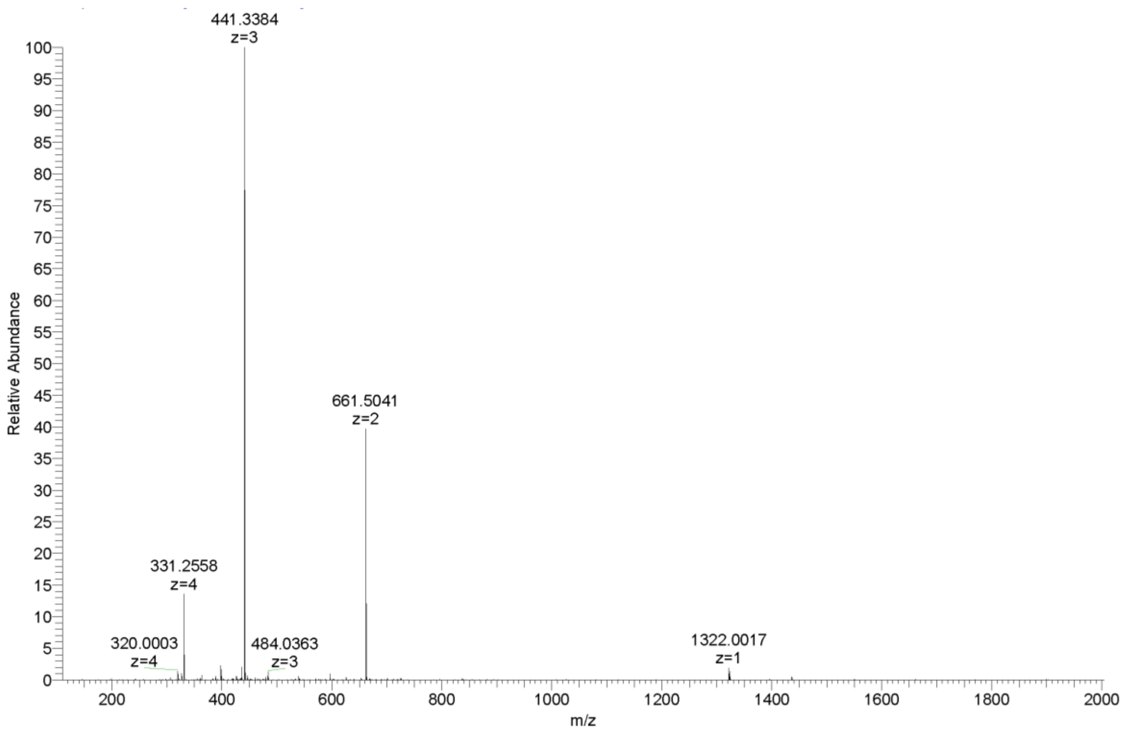
441.3384
z=3
661.5041
z=2
331.2558
z=4
320.0003
z=4
484.0363
z=3
1322.0017
z=1
Relative Abundance
m/z

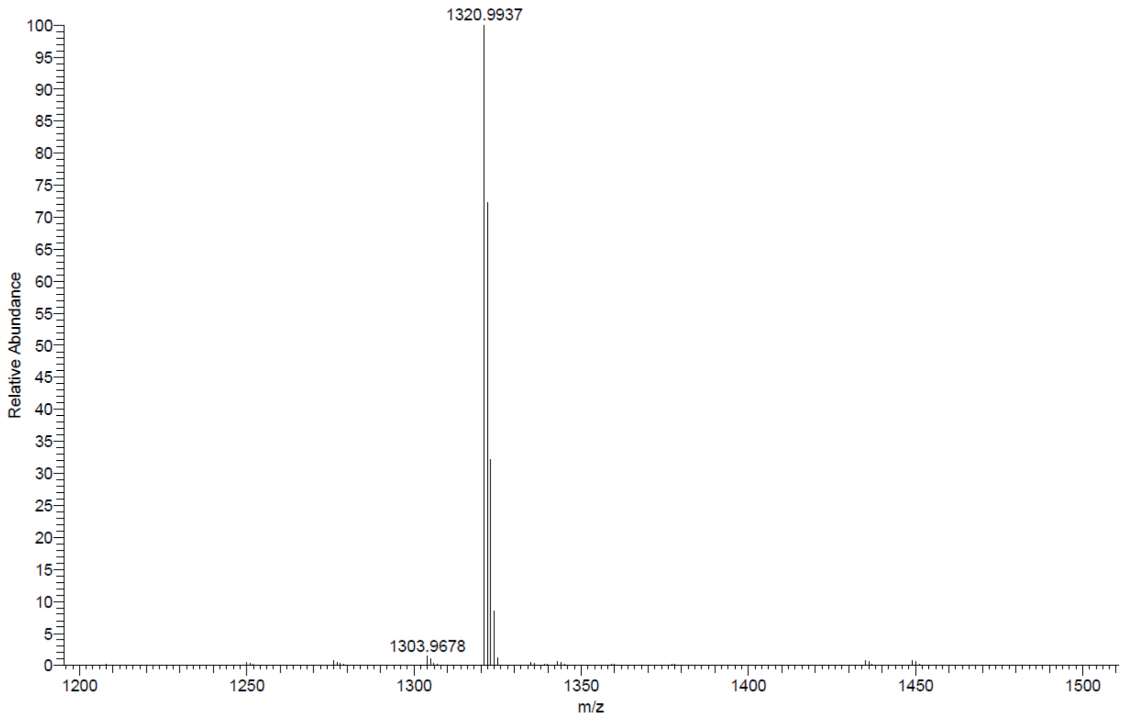
1320.9937
1303.9678
Relative Abundance
m/z

KK*ll*K*ll*K*lll* (EB8) was obtained as a white solid after preparative RP-HPLC (36.9 mg, 17.8 %). **Analytical RP-HPLC**: t_R = 3.44 min (A/D 100:0 to 0:100 in 7.0 min, λ = 214 nm). **MS** (ESI$^+$): $C_{66}H_{128}N_{16}O_{11}$ calc./obs. 1321.9948/1322.0028 Da [M+H]$^+$.

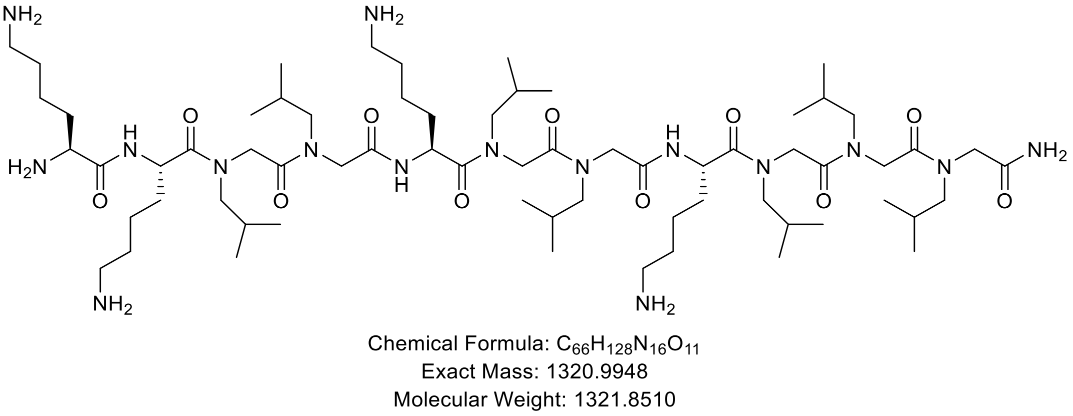

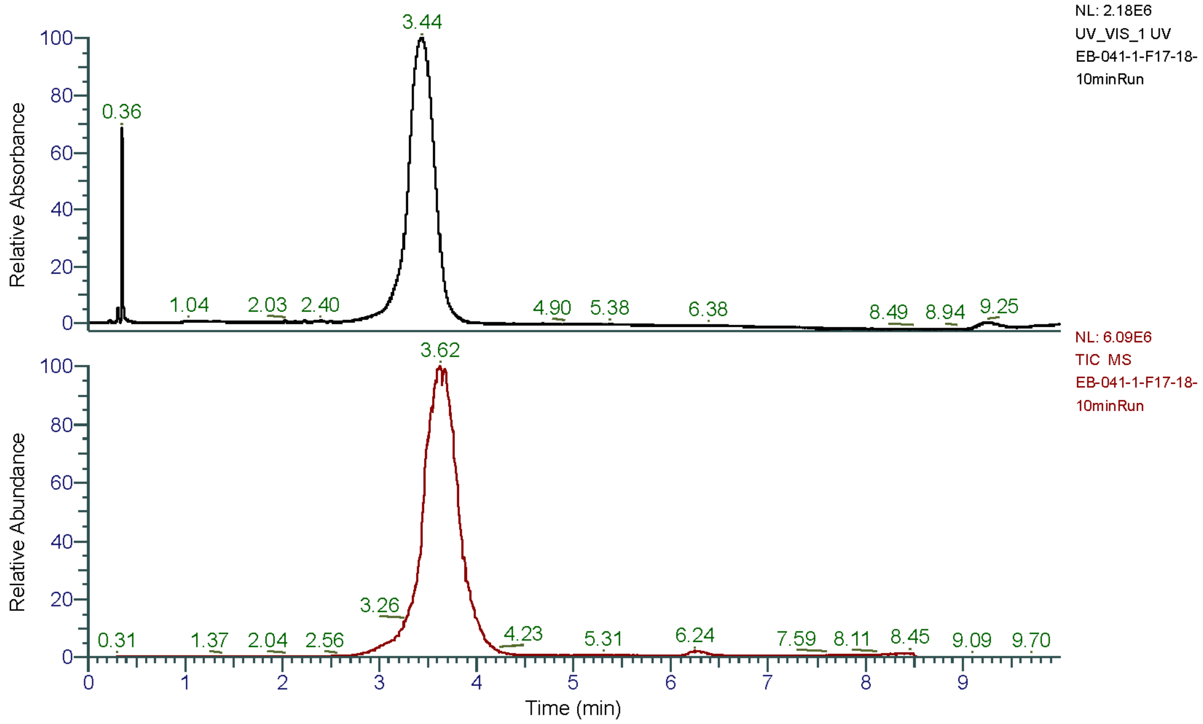

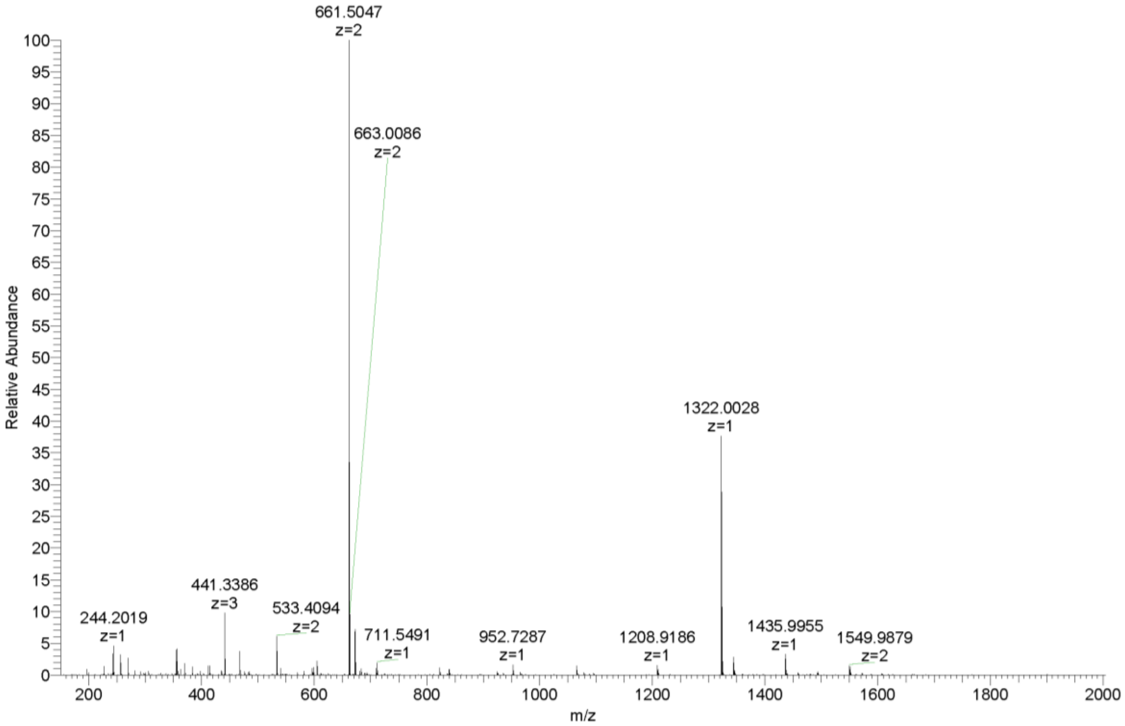
661.5047
z=2
663.0086
z=2
1322.0028
z=1
441.3386
z=3
244.2019
z=1
533.4094
z=2
711.5491
z=1
952.7287
z=1
1208.9186
z=1
1435.9955
z=1
1549.9879
z=2
Relative Abundance
m/z

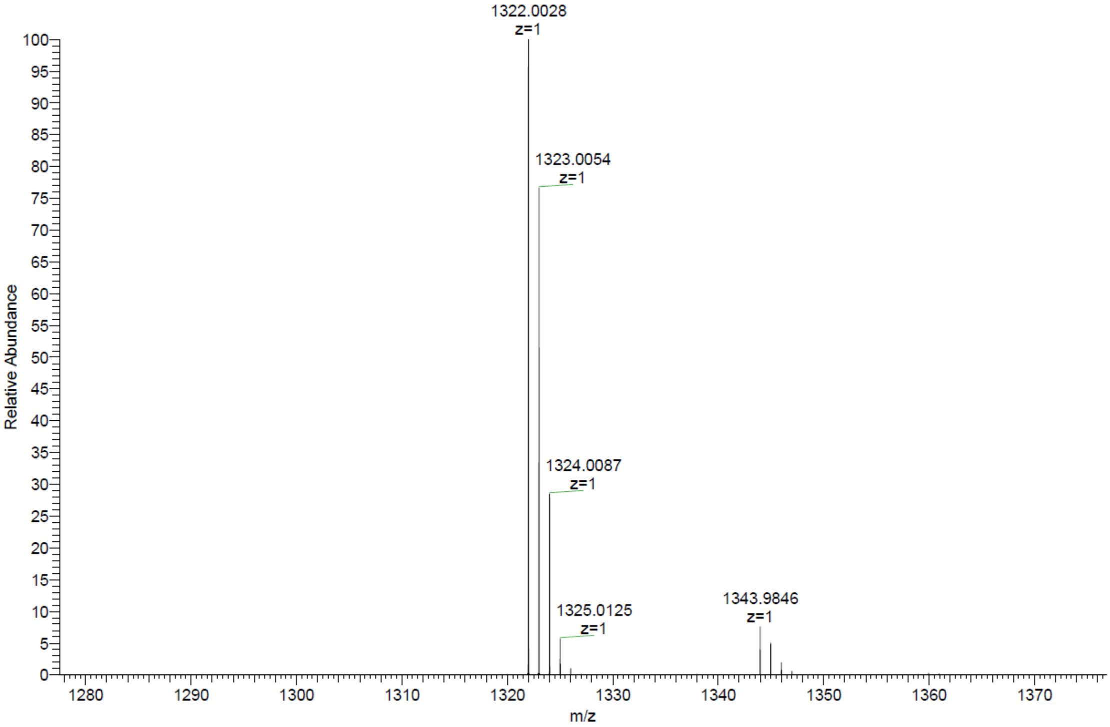
1322.0028
z=1
1323.0054
z=1
1324.0087
z=1
1325.0125
z=1
1343.9846
z=1
Relative Abundance
m/z

K*k*L*l*K*l*L*k*L*l*L (EB9) was obtained as a foamy white powder after preparative RP-HPLC (35.6 mg, 17.3 %). **Analytical RP-HPLC**: t_R = 3.31 min (A/D 100:0 to 0:100 in 7.0 min, λ = 214 nm). **MS** (ESI$^+$): $C_{66}H_{128}N_{16}O_{11}$ calc./obs. 1321.9948/1322.0020 Da $[M+H]^+$.

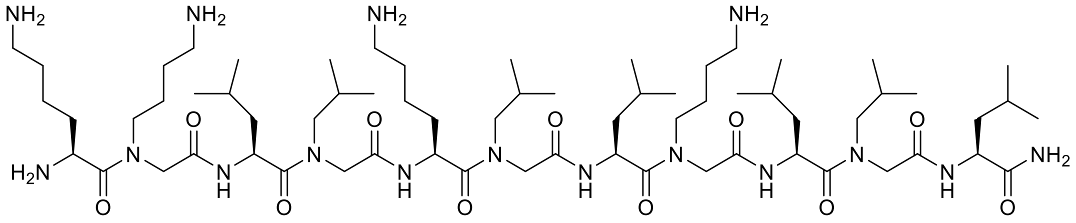

Chemical Formula: $C_{66}H_{128}N_{16}O_{11}$
Exact Mass: 1320.9948
Molecular Weight: 1321.8510

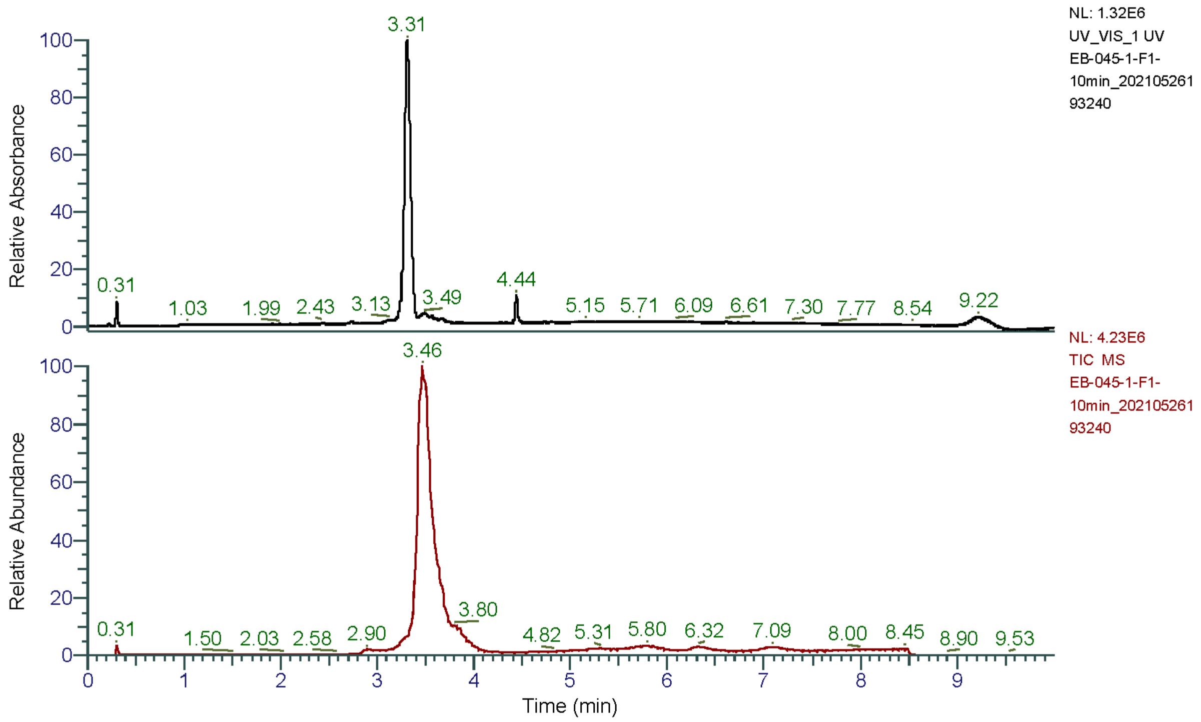

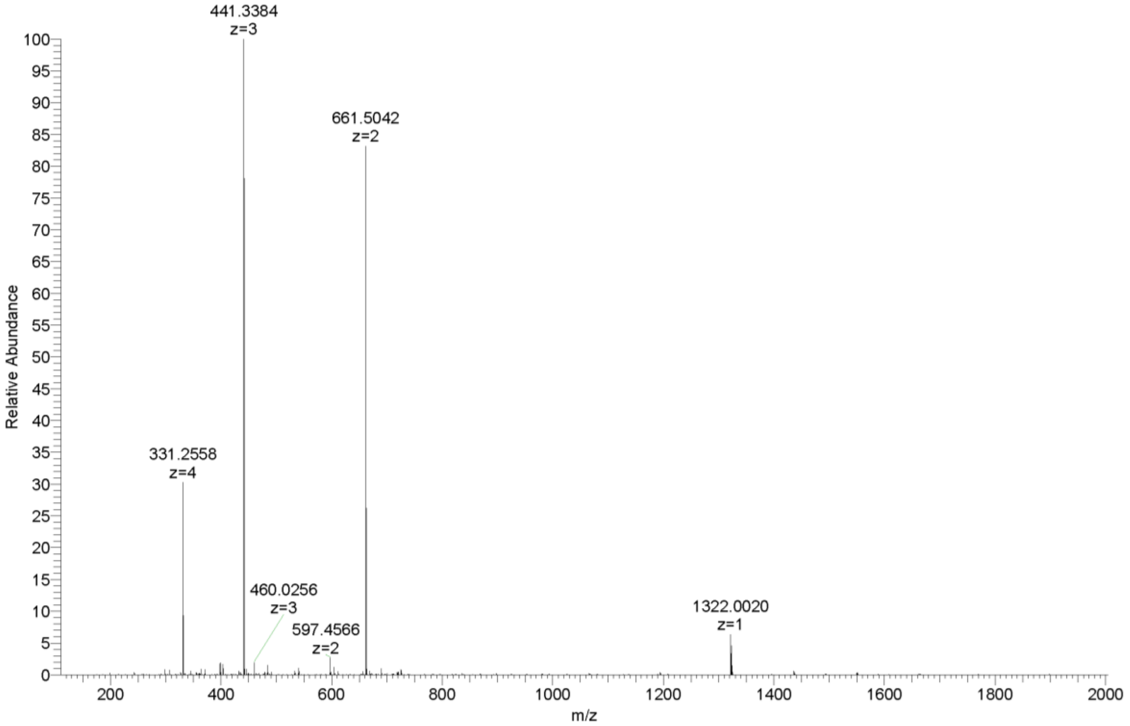

441.3384
z=3
661.5042
z=2
331.2558
z=4
460.0256
z=3
597.4566
z=2
1322.0020
z=1
Relative Abundance
m/z

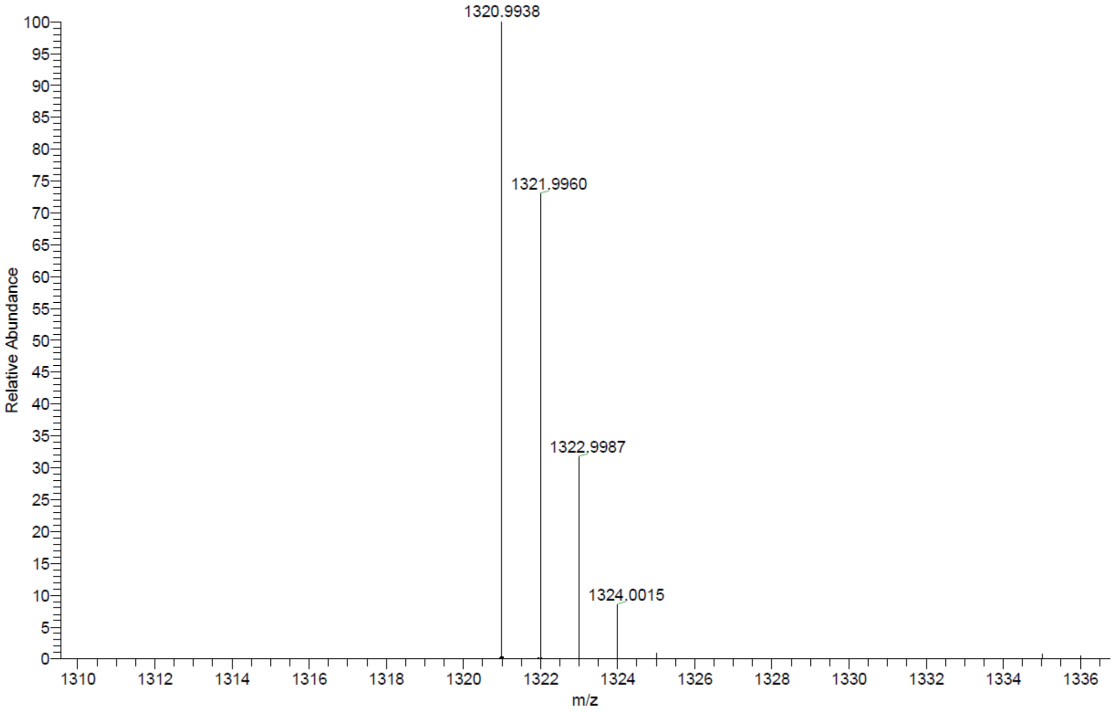

1320.9938
1321.9960
1322.9987
1324.0015
Relative Abundance
m/z

_k_K_l_L_k_L_l_K_l_L_l_ (EB10) was obtained as a foamy white powder after preparative RP-HPLC (55.6 mg, 27.0 %). **Analytical RP-HPLC**: t_R = 3.49 min (A/D 100:0 to 0:100 in 7.0 min, λ = 214 nm). **MS** (ESI^+): $C_{66}H_{128}N_{16}O_{11}$ calc./obs. 1320.9948/1320.9960 Da $[M+H]^+$.

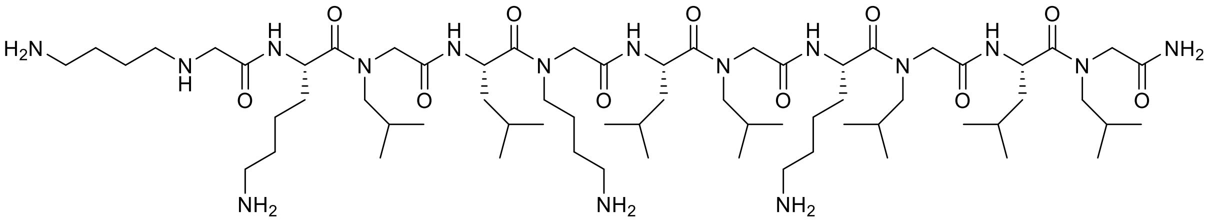

Chemical Formula: $C_{66}H_{128}N_{16}O_{11}$
Exact Mass: 1320.9948
Molecular Weight: 1321.8510

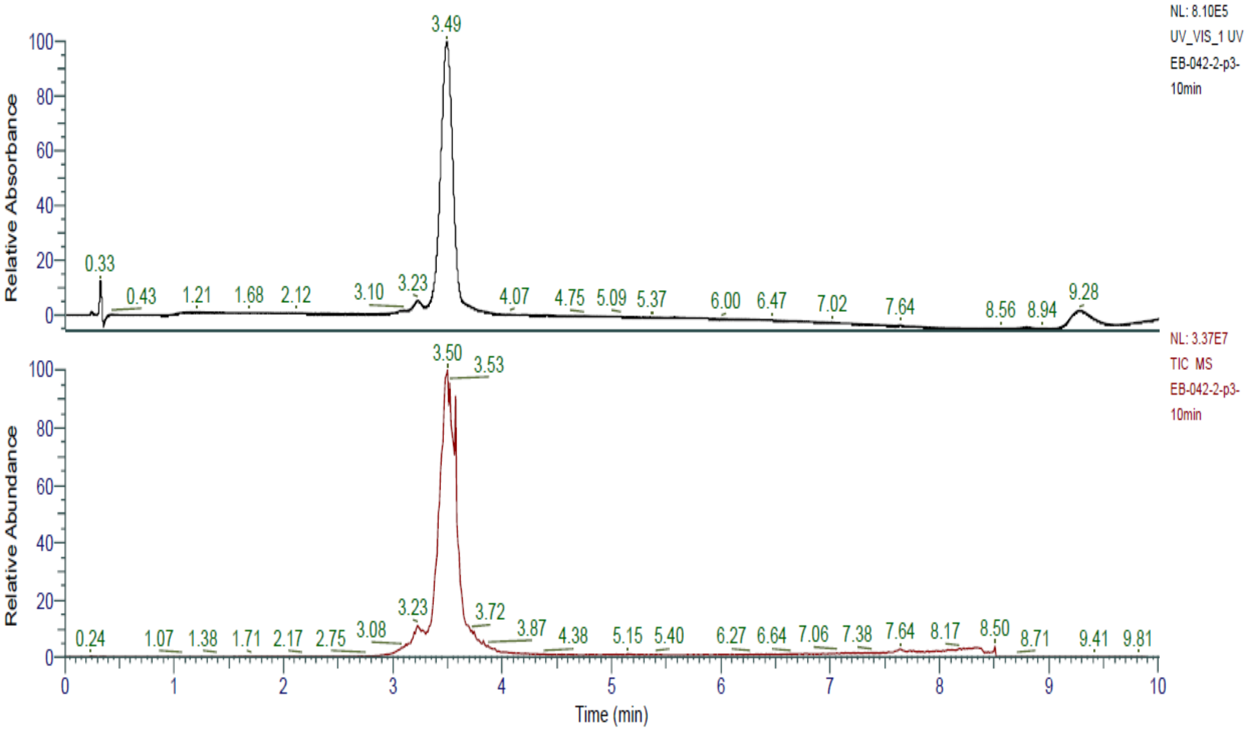

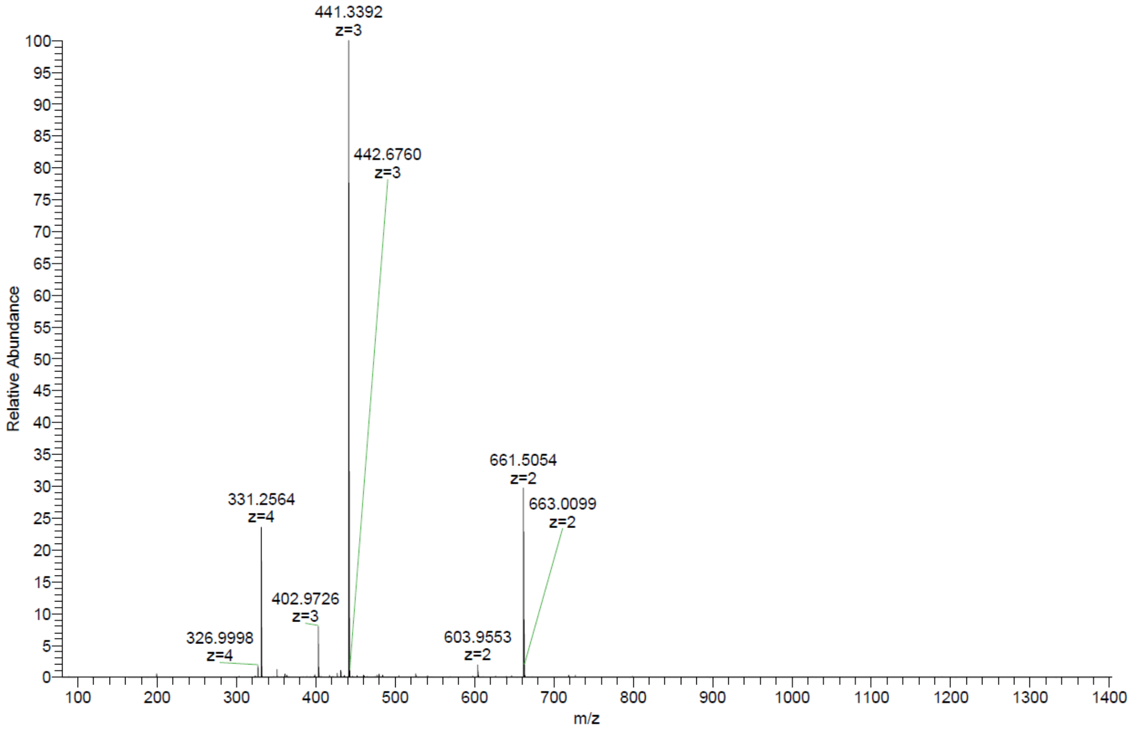
441.3392
z=3
442.6760
z=3
331.2564
z=4
326.9998
z=4
402.9726
z=3
661.5054
z=2
663.0099
z=2
603.9553
z=2
Relative Abundance
m/z

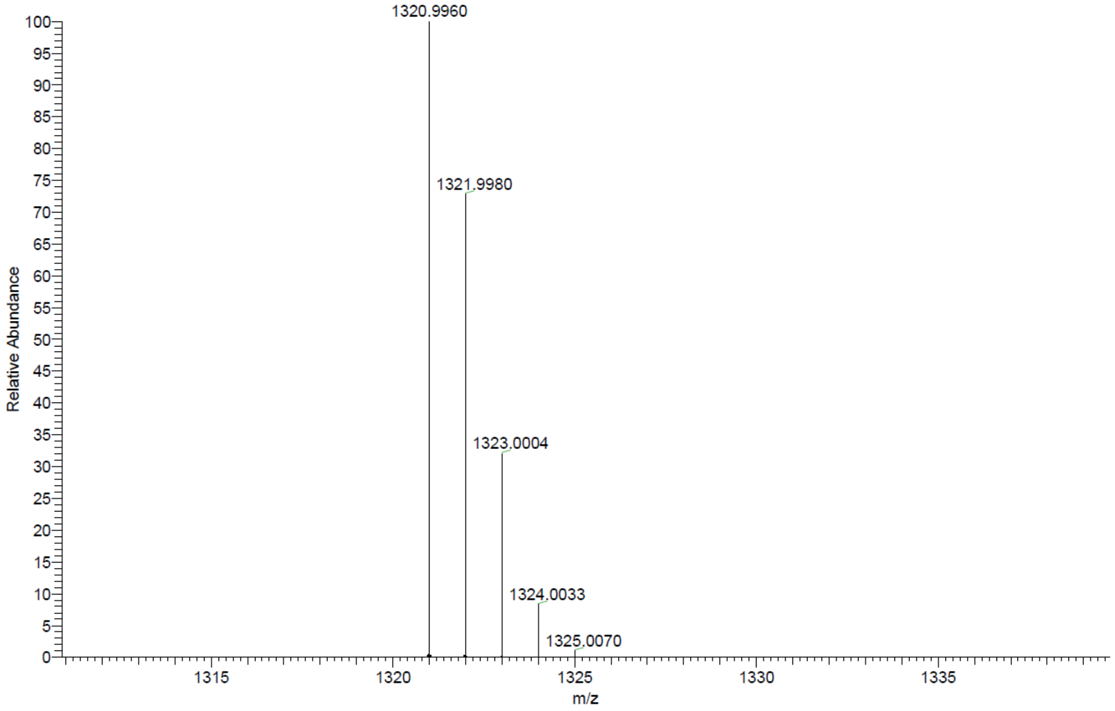
1320.9960
1321.9980
1323.0004
1324.0033
1325.0070
Relative Abundance
m/z

kkllkllklll **(EB11)** was obtained as a colorless solid after preparative RP-HPLC (22.8 mg, 11.0 %). **Analytical RP-HPLC**: t_R = 3.44 min (A/D 100:0 to 0:100 in 7.0 min, λ = 214 nm). **MS** (ESI$^+$): $C_{66}H_{128}N_{16}O_{11}$ calc./obs. 1321.9948/1322.0032 Da $[M+H]^+$.

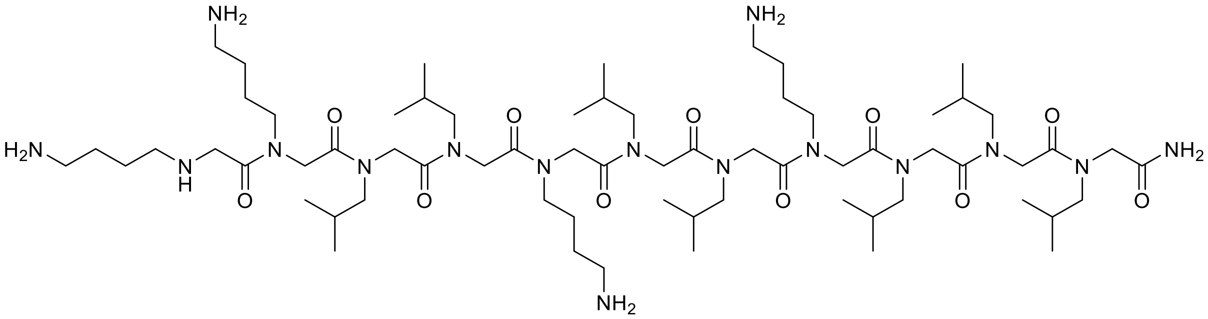

Chemical Formula: $C_{66}H_{128}N_{16}O_{11}$
Exact Mass: 1320.9948
Molecular Weight: 1321.8510

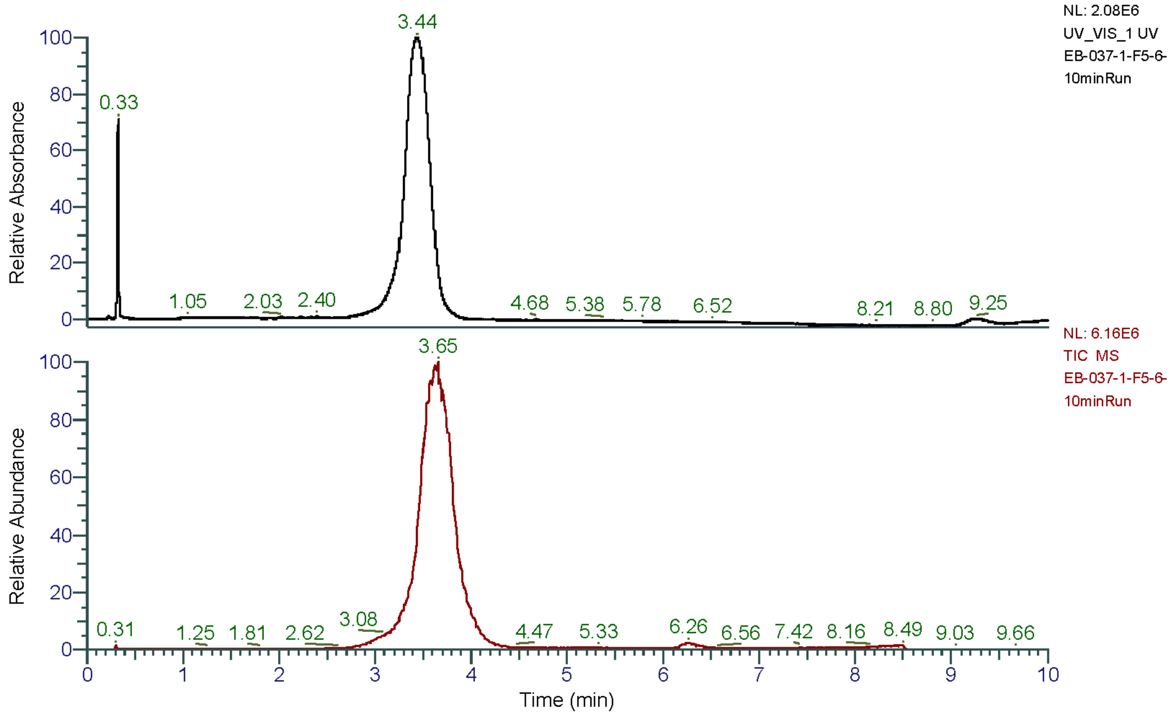

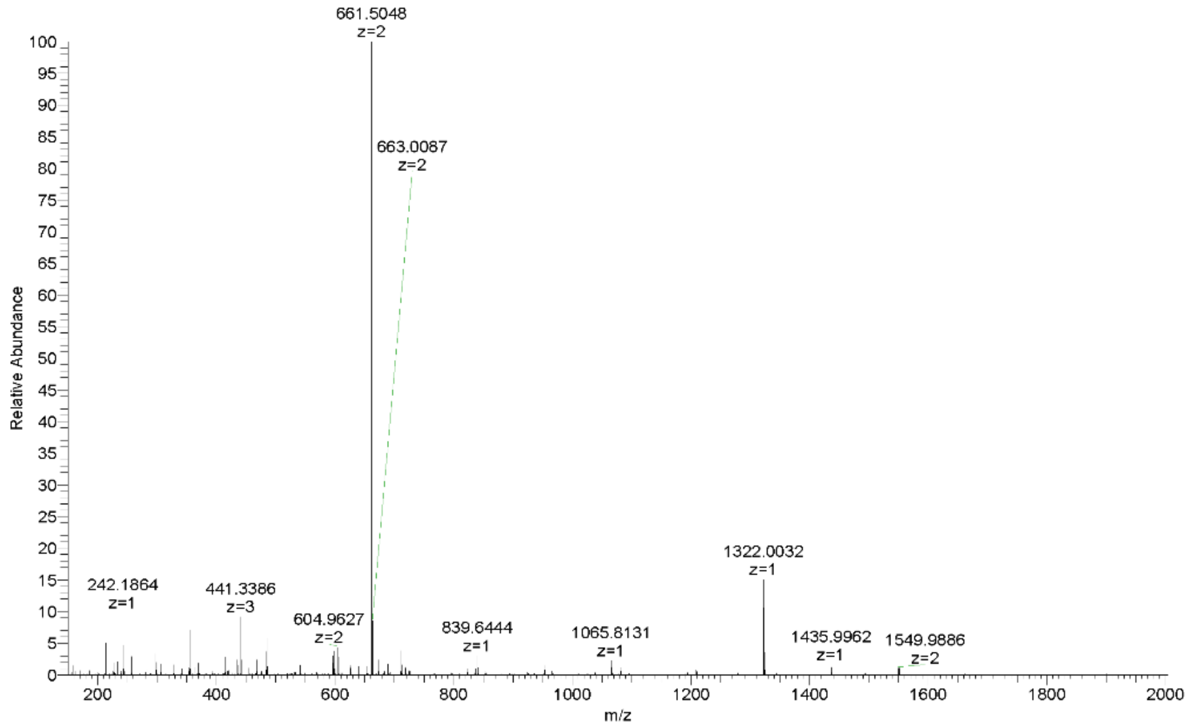
661.5048
z=2
663.0087
z=2
1322.0032
z=1
242.1864
z=1
441.3386
z=3
604.9627
z=2
839.6444
z=1
1065.8131
z=1
1435.9962
z=1
1549.9886
z=2
Relative Abundance
m/z

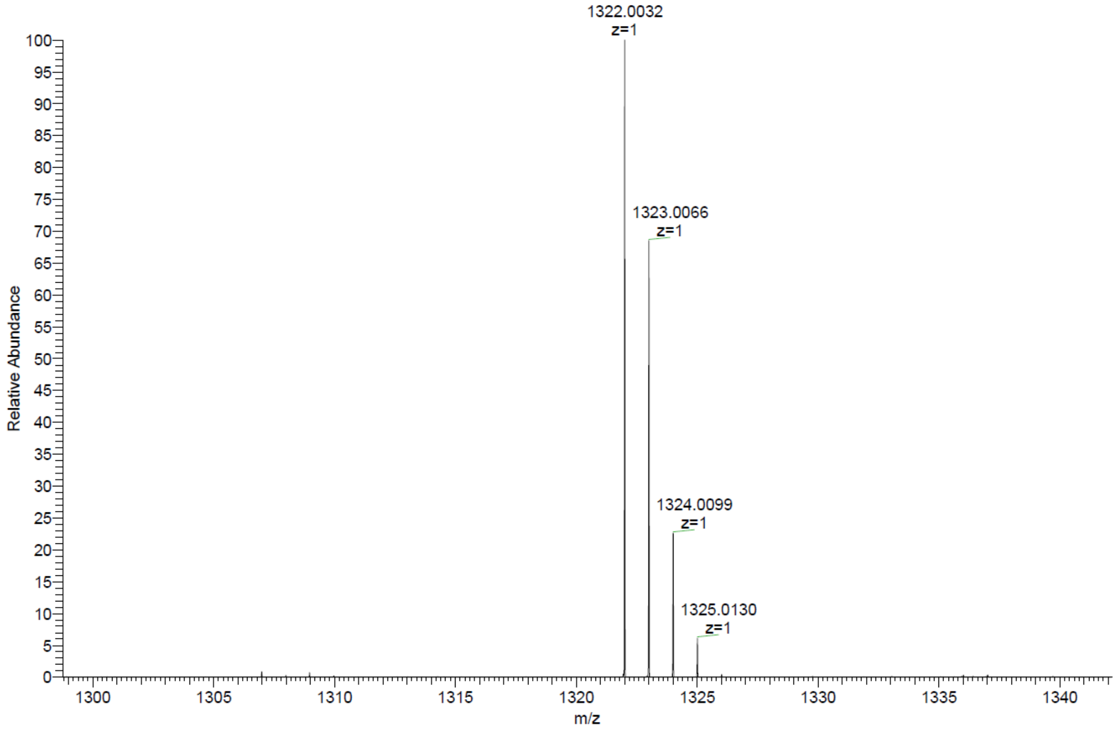
1322.0032
z=1
1323.0066
z=1
1324.0099
z=1
1325.0130
z=1
Relative Abundance
m/z
